# The latent structure of diversification from relatedness and network embeddings

**DOI:** 10.1371/journal.pone.0344958

**Published:** 2026-10-22

**Authors:** Niclas Frederic Sturm, Cristian Candia, Bruno Damásio, Flávio L. Pinheiro

**Affiliations:** 1 Nova IMS, Universidade Nova de Lisboa, Lisbon, Portugal; 2 Computational Research in Social Science Laboratory, School of Engineering and School of Government, Universidad del Desarrollo, Santiago, Chile; 3 Facultad de Ingeniería, Instituto de Data Science, Universidad del Desarrollo, Santiago, Chile; 4 Northwestern Institute on Complex Systems, Kellogg School of Management, Northwestern University, Evanston, United States of America; Lodz University of Technology: Politechnika Lodzka, POLAND

## Abstract

Economic diversification is commonly modeled using co-occurrence-based measures of relatedness that quantify pairwise overlap among activities in a capability network. Although such measures are derived from network structure, they operate at the level of first-order neighborhood relations and summarize proximity through local co-occurrence. They do not explicitly encode higher-order structural context, such as indirect connectivity patterns or global positional similarity, into a continuous representation of entities and activities. We develop an embedding-based framework that learns such representations from network topology, enabling the construction of interpretable geometric features, including capability alignment, displacement, and portfolio entropy, that extend standard measures used in economic complexity. We apply this framework to two systems: international trade (1998–2023) and public procurement firms (2009–2024). The resulting representations reveal different structural organization across scales: firms exhibit modular clustering patterns, whereas countries occupy broader, nested capability configurations. In predictive tasks for product and activity specialization, embedding-derived features provide additional explanatory power beyond relatedness in several settings, particularly when forecasting variation in the magnitude of comparative advantage. For country-level entry events, however, persistence remains the strongest predictor. Our results show that relatedness remains the dominant predictor of entry events. In contrast, embedding-derived features become more informative in specific settings, particularly for modeling variation in specialization magnitude and in low-relatedness regimes. More broadly, the findings indicate that diversification depends on structural signals that are both prediction-target-dependent and scale-dependent, with relatedness and embedding-derived features capturing different aspects of the capability space (local overlap vs. structural position).

## 1. Introduction

Understanding how entities diversify into new activities has been a central contribution of Economic Complexity research [1]. In the context of international trade, development is conceptualized as a gradual process of entry into increasingly complex activities, constrained by the structure of an underlying knowledge network, the product space [2,3]. This framework is formalized through the “principle of relatedness,” which predicts that diversification is more likely toward activities that share capabilities with those already present in an entity’s portfolio [4]. Empirical evidence for this principle extends beyond countries [5–7] to cities [8–10], regions [11–13], firms [14–16], technological domains [17,18], scientific collaboration [19], software ecosystems [20], research areas [21,22], and cultural production such as music [23]. Measures derived from these network structures have also been linked to outcomes including growth, emissions, and inequality [24–27].

Across these applications, relatedness is typically grounded in the concept of absorptive capacity [28], emphasizing how an entity’s existing portfolio conditions feasible future expansions. Operationally, most relatedness measures are constructed from co-occurrence statistics that quantify portfolio coherence [29] via pairwise overlap in observed activities. In geographic applications, these overlaps can often be estimated from industrial-sector linkages [8]; at the firm level, however, the measurement of relatedness remains shaped by trade-offs between theoretical coherence and data availability [30].

Despite its empirical reach, the principle of relatedness is operationalized through statistics derived from first-order co-occurrence, which summarize local proximity in the network. While this operationalization has proven effective for predicting entry patterns, it does not explicitly distinguish between diversification associated with immediate overlap and diversification associated with broader structural position in the capability network. This distinction matters because, without it, predictive signals cannot be clearly interpreted as reflecting local capability overlap versus broader capability organization. This raises a structural question: to what extent does diversification depend on direct overlap between activities, and to what extent is it associated with structural position within the capability network, including indirect connectivity and structural similarity?

Recent work has expanded diversification research by applying machine learning methods to prediction tasks across countries, firms, and technologies. These studies include supervised models trained on bipartite activity matrices (e.g., country-product or firm-technology incidence data), such as logistic regression, naive Bayes, tree-based methods, and neural networks [31–34]; matrix-completion approaches for reconstructing country-product structures [35]; feature-based approaches using similarities between entities or network-derived descriptors to predict technological development and firm performance [36,37]; and methods that infer relatedness from unstructured data sources [38]. More recently, diversification-related applications have also introduced embedding-based representations, including text-derived embeddings, embeddings from model outputs, and network embeddings derived from bipartite matrices [34,39]. Collectively, this literature expands the predictive toolkit for diversification analysis, but it also shifts attention toward how structural information is represented and interpreted in downstream models.

A common limitation across these approaches is that many predictive models are trained on incidence matrices or feature sets whose relation to the underlying capability structure is difficult to interpret. As a result, models can identify which observed inputs are predictive of diversification outcomes without clarifying whether the signal reflects direct overlap, higher-order structural position, or structural similarity consistent with shared capability requirements. This distinction is central in economic complexity because relatedness is used not only as a predictor, but also as a mechanism linking current portfolios to feasible expansion paths [4,28,29]. At the same time, increased model flexibility can further reduce interpretability, especially in policy-oriented settings where the usefulness of predictions depends on identifying actionable structural drivers rather than predictive accuracy alone [35,37,40]. These limitations motivate a representation framework that can encode higher-order network structure while remaining interpretable in terms of diversification mechanisms.

Because relatedness is defined on networks of proximity among activities, a relevant extension is to use network representations that encode structural information beyond pairwise overlap. In contrast to co-occurrence-based relatedness measures, node embeddings provide continuous representations learned from graph topology that can incorporate multi-step connectivity, neighborhood structure, and positional similarity [41]. This makes them a suitable representation class for studying diversification when the relevant signal may depend on structural position in capability networks beyond direct co-occurrence. Related embedding approaches have been used to model firm-level technological trajectories [42] and country-level proximity in domains such as scientific migration and geopolitical priorities [43,44]. However, these applications rely primarily on unstructured text or non-proximity-based relational data and therefore do not directly address how embeddings derived from capability-network topology can be integrated with theory-grounded constructs from economic complexity. Closer aligned with the methodology used in this article is the literature on detecting irregularities in economic or financial networks, where, in order to incorporate structural information, node representations are used for downstream classification tasks [45–47].

In this study, we construct temporally aligned node embeddings from proximity networks and derive interpretable geometric features, such as capability alignment, displacement, and portfolio entropy, that operationalize how entities are positioned and move within a capability space [48,49]. We compare the predictive contribution of these embedding-derived features with standard relatedness-based measures and evaluate their interpretive value in downstream models, treating them as complementary descriptions of diversification structure rather than as replacements. To preserve interpretability in downstream models, we combine theory-grounded feature design with SHAP-based feature attribution [50], enabling us to assess which aspects of local proximity and higher-order structural position are most informative for diversification outcomes. The contribution of this study is, therefore, to introduce a common predictive framework to separate when local overlap versus structural-position information is most informative for diversification. Specifically, we show that this distinction is both prediction-target dependent (entry vs. specialization magnitude) and scale-dependent (firms vs. countries), clarifying the complementary roles of canonical relatedness and embedding-derived representations in diversification analysis.

We evaluate this framework in two large-scale datasets spanning different levels of aggregation: a micro-level dataset of firm activities in public procurement (2009–2024) and a country-level dataset of international trade (1998–2023). This design enables comparison across systems with different structural organization and temporal dynamics. We first characterize the embedding spaces using clustering and correlation analyses, and then assess downstream predictive performance by modeling product and activity specialization as both a classification and a regression task. This distinction separates the prediction of activity entry from the prediction of variation in the magnitude of comparative advantage. In addition to relatedness and embedding-derived features, we include graph-level and entity-level covariates. This allows us to compare the relative contribution of each feature class across tasks and levels of aggregation.

## 2. Materials and methods

### 2.1. Data

We use two datasets at different levels of aggregation: (i) international trade data (country level) and (ii) public procurement data (firm level). Because the same methodological pipeline is applied to both countries and firms, we use the term *entity* to refer to both throughout the paper. To improve computational feasibility and reduce noise, we apply domain-specific filtering procedures before constructing the proximity networks, embeddings, and downstream prediction models. In Fig 1, we illustrate the process from data curation to the modeling stage.

**Fig 1:**
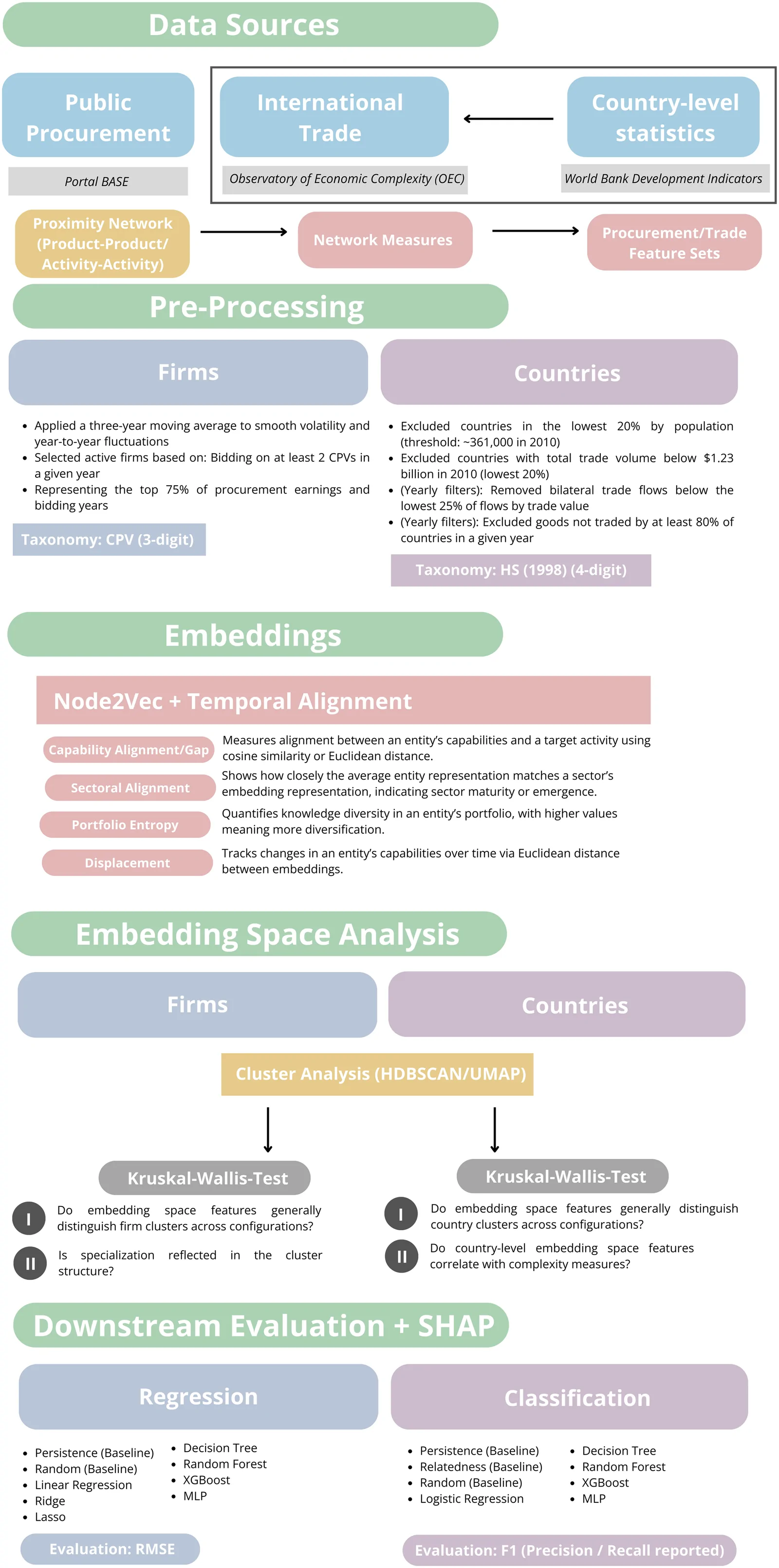
Pipeline for public procurement and international trade: Data ingestion and processing, generation of embeddings, embedding space analysis, and predictive modeling.

We extract international trade data from the Observatory of Economic Complexity, covering bilateral trade relationships from 1998 to 2023 [51], using the HS (Rev. 1998) classification reported at the HS6 level. For the analyses in this study, we aggregate products to the 4-digit level (HS4). We apply a two-stage filtering procedure to reduce noise and computational cost. First, we exclude countries in the bottom 20% of the population distribution (approximately 361,000 inhabitants in 2010) that also have total trade volume below $1.23 billion in 2010 (the midpoint year of the sample period). Second, we apply year-by-year filters, which exclude the lowest 20% of trade flows by trade value and remove products with low geographic prevalence, that is, products absent from at least 80% of countries in a given year.

The final trade dataset contains approximately 4.0 million observations and 1,231 distinct products at the HS4 level. We further supplement the trade data with country-level indicators from the World Development Indicators (WDI) database published by the World Bank [52]. Features for this domain are summarized in Table 2.

In the procurement domain, we use data from Portal BASE, an open data portal provided by the Portuguese government [53], covering the years 2009–2024. The contract-level data include bidders’ and contracting authorities’ tax identification numbers (TINs), a contract activity code based on the Common Procurement Vocabulary (CPV) classification, and contract dates. Table 1 describes these features.

**Table 1:** Feature table for public procurement data. For each feature, the Q1-Q3 range is reported. For embedding space features, a standard deviation is provided for each generation parameter set. “HHI” refers here to the Herfindahl-Hirschman Index (HHI).

Feature Type	Range (Q1, Q3)	Features
Network Measures	[0.2852, 0.6441]	Degree Centrality
	[0.0319, 0.043]	Clustering Coefficient
	[2824, 12420]	Triangle Count
	[0.0001, 0.0029]	Betweenness Centrality
	[0.0124, 0.0667]	Eigenvector Centrality
Embedding Space	0.1695 ± 0.041	Capability Alignment
	4.7403 ± 0.6485	Capability Gap
	0.959 ± 0.1948	Displacement
	0.4752 ± 0.2084	Portfolio Entropy
	0.346 ± 0.0103	Sectoral Alignment
Firm-level	[0.1605, 0.4876]	Geographic HHI
	[0.0454, 0.1524]	Public Entity HHI
	[0.1765, 0.4566]	Activity HHI
	[18,70]	N° Contracts Won
	[5.7469, 6.6522]	Total Awards (Log10)
	[0.1062, 0.5]	Tender Winning Rate
	[4.3002, 4.7819]	Mean Contract Value per CPV (Log10)
Economic Complexity	[0.01, 0.065]	Relatedness
	[6,15]	Diversity
	[12,78]	Ubiquity

The raw dataset contains ca. 94,000 firms and 1,555,000 contracts. We then retain a subset of active firms based on activity and scale criteria to reduce noise in firm-level activity portfolios. To facilitate sectoral activity attribution, we include only contracts with a single winner. Then, at the firm level, we filter for firms that are active in at least 2 distinct CPV groups in any given year and are in the 75th percentile of procurement earnings (corresponding to ca. 37,000€) over their entire activity span. Finally, we retain firms that have at least 3 years of activity, with a maximum of two years in between years with activity. Taken together, these filters lead to a 56% reduction in the number of contracts (679,000) and cover the 2.5% (2,401) most active firms. The resulting market is of a size comparable to the active core observed in other studies for similar datasets [54]. Because procurement activity exhibits stronger year-to-year volatility than trade, we smooth firm activity profiles using a three-year moving average. The final public procurement dataset then comprises 3.5 million firm/activity/year pairs for 312 distinct CPV groups.

For notational simplicity, the measures introduced in the following sections are written without explicit time indices. In the trade application, they are computed separately for each year; in the public procurement application, they are computed on the corresponding three-year moving-average windows described above.

### 2.2. Relatedness

Relatedness between activities is typically constructed from the specialization profile of those entities in whose portfolios they appear, which requires a suitable measure of specialization. At the country level, the specialization measure most commonly used is the *Revealed Comparative Advantage* (RCA), also known as the Balassa index [55], where a country’s export share of a product relative to the world share is interpreted as a comparative advantage. This interpretation is specific to countries, but the same ratio can be computed for any unit. Because we apply it purely as a measure of specialization intensity at both the country and firm level, we will use the term *revealed specialization* (RS) throughout.

Let $X_{u,a}$ denote the intensity of entity *u* in activity *a* (e.g., trade value or number of bids in procurement). The continuous RS is defined as


$$\begin{array}{c} R_{u,a}=\frac{X_{u,a}}{\sum_{a^{\prime }}X_{u,a^{\prime }}}/\frac{\sum_{u^{\prime }}X_{u^{\prime },a}}{\sum_{u^{\prime },a^{\prime }}X_{u^{\prime },a^{\prime }}} \end{array}$$ (1)

In the international trade domain, *u* is a country and *a* is an HS4 product code, while in the public procurement domain, *u* is a firm and *a* is a 3-digit CPV group. Concretely, CPV groups include codes such as 452 (Construction work), 336 (Medical equipment/pharmaceutical products), and 722 (Computer programming services), while analogous HS4 classification codes include 7308 (structures and parts of structures, of iron or steel), 3004 (pharmaceutical products), and 8471 (automatic data-processing machines). Importantly, the CPV taxonomy also includes services, whereas HS4 typically only refers to tradable goods.

Eq. 1 is algebraically a location quotient, and its interpretation of an activity *a* being over-represented in entity *u*’s portfolio relative to the reference system as a whole is well defined for any unit. At the firm level, the RS therefore identifies those activities that a firm is relatively specialized in compared to the overall market. Beyond this interpretation, it is a scale-invariant measure, normalized both over an entity’s portfolio and over its reference system. The same measure, sometimes under different names, has been used to quantify firm export specialization [32,56], firm technological specialization [17], and technological specialization at the regional [57,58] and city level [59].

To derive the relatedness measure in its canonical form, we construct a binary specialization matrix $M_{u,a}$ by thresholding the RS at 1:


$$\begin{array}{c} M_{u,a}=\left\{ \begin{array}{cc} 1, & \text{if }R_{u,a}\geq 1,\\ 0, & \text{otherwise.} \end{array}\right. \end{array}$$ (2)

The matrix $M_{u,a}$ is used to define two standard descriptors: entity diversity, $k_{u}=\sum_{a}M_{u,a}$, i.e., the number of RS of an entity, and activity ubiquity, $k_{a}=\sum_{u}M_{u,a}$.

Proximity between activities is computed from co-occurrence under the binarized specialization matrix. Using the standard symmetric normalization, proximity between activities *a* and $a^{\prime }$ is defined as


$$\begin{array}{c} \phi_{a,a^{\prime }}=\frac{\sum_{u}M_{u,a}M_{u,a^{\prime }}}{\max(k_{a},k_{a^{\prime }})}, \end{array}$$ (3)

which is equivalent to the minimum of the two conditional co-occurrence probabilities. The resulting proximity matrix defines the activity network (the “product space” in the trade setting).

The relatedness construct now connects entity-activity specialization and proximity between activities. Given an entity’s portfolio, relatedness is a weighted ratio of proximities and measures how closely a target activity aligns with the activities in which the entity is already specialized. We define relatedness as


$$\begin{array}{c} \omega_{u,a}=\frac{\sum_{a^{\prime }\neq a}M_{u,a^{\prime }}\phi_{a,a^{\prime }}}{\sum_{a^{\prime }\neq a}\phi_{a,a^{\prime }}}, \end{array}$$ (4)

where $\omega_{u,a}$ is the relatedness between an entity *u* and an activity *a*. The target activity is excluded from the summation to avoid mechanical inflation when the entity is already specialized in *a*. Activities with higher relatedness are more closely aligned with an entity’s current capabilities, while lower relatedness indicates more distant opportunities.

### 2.3. Embedding space

To understand diversification processes, economic complexity has developed several constructs, such as relatedness, diversity, and ubiquity, and, in turn, measures of complexity. All of these measures are mathematically dependent on a formulation of the RS. It is well established that the structure of networks conditions the flow of knowledge within them [22,60,61]. This is particularly pertinent in the case of diversification processes. However, beyond the constructs explicitly derived above, our understanding of how the knowledge network topology can complement our understanding of capabilities is limited.

It is for this reason that we introduce here features derived from an embedding space representation of goods/services and economic activities. The common root between the embedding space and the canonical measures is the underlying proximity network. In this embedding space, these can be represented as high-dimensional vectors learned from the underlying knowledge networks.

The representation of entities within the latent space is constructed through a two-step hierarchical process. We distinguish between first-order embeddings, which represent specific activities (i.e., the nodes in the network – CPV groups (3-digit) for public procurement and HS (1998) product codes (4-digit) for international trade), and second-order embeddings, which represent an entity’s (firms’ and countries’) aggregate capability profile.

In particular in the domain of economic complexity, relatedness is increasingly seen as a dynamic and time-variant measure whose changes over time are in need of explanation [62]. This dynamic element of the measures characterizing diversification processes, similar for the related measures ubiquity and diversity, as well as the complexity indices, is tied to the underlying activity network representation, which is fixed due to its taxonomic nature. The dynamics are mainly to be found in the reweighting of edges (the proximity) and occupancy of nodes, i.e., the RS of the entity in that activity. This contrasts with macro-changes that occur more at the level of the related network structures, such as the specialization matrix (Eq. 2), where special dynamics are at play [63]. At that level, the network is directly subject to processes that could be approached by node emergence models [64,65] or the formation of new edges in the economic network of firms/activities [15,32,66] or countries/goods, the original domain of Economic Complexity [2,3]. Due to the relatively stable nature of the network properties at the level of activity-activity network, we therefore opted for an approach that generates snapshots of this network at regular intervals.

Before the embedding process, a snapshot graph $G_{t}=({𝒱}_{t},E_{t})$ is constructed for each period, using only the information available up to period *t*. The nodes ${𝒱}_{t}$ represent activities or produc*t*s (by taxonomy code) at time *t*, and the edges $E_{t}$ are derived from the proximity matrix, with edge weights given by the proximi*t*y $\phi_{a,a^{\prime }}$ (Eq. 3). Since the trade proximity networks are considerably denser than those of the procurement domain, we apply a sparsification step in the trade domain, retaining a Maximum Spanning Tree and reintroducing edges with proximity of at least 0.5; details are provided in S1 File Section S1.

We apply *Node2Vec* [49] to each snapshot graph $G_{t}$, learning a first-order activity embedding for every node. Node2Vec then maps each activity $a\in {𝒱}_{t}$ to a dense vector ${\vec{e}}_{a}\in {\mathbb{R}}^{d}$, where *d* is the embedding dimensionality, such that the proximity, as encoded by the edge weight in $G_{t}$, is reflected as similarity in the embedding space. Because these embeddings are trained independently per period, and the training objective is invariant to orthogonal transformations, consecutive latent spaces are structurally equivalent but arbitrarily rotated relative to one another. Following the logic of tNodeEmbed [48] and approaches to studying semantic change [67], we resolve this through backward-looking orthogonal Procrustes alignment [68], estimating from the overlapping node set between consecutive periods the orientation necessary to map the embedding space of period *t* onto the orientation of the space at t−1. The alignment is thus backward-looking, avoiding temporal leakage in the embedding generation process. We denote the aligned activity embeddings by ${\tilde{\vec{e}}}_{a}$. The measures in*t*roduced below are based on these aligned embeddings. The complete alignment procedure is described in S1 File Section S1.

Higher-order entity embeddings are then constructed by aggregating the aligned activity embeddings. Given the set of activities ${𝒜}_{u}$ of entity *u* with non-zero $R_{u,a}$, the entity embedding ${\tilde{\vec{e}}}_{u}$ is defined as the RS-weighted average:


$$\begin{array}{c} {\tilde{\vec{e}}}_{u}=\frac{1}{\sum_{a\in {𝒜}_{u}}R_{u,a}}\sum_{a\in {𝒜}_{u}}R_{u,a}\cdot {\tilde{\vec{e}}}_{a} \end{array}$$ (5)

In geometrical terms, ${\tilde{\vec{e}}}_{u}$ represents the structural centroid of an entity’s portfolio, where an entity’s embedding is pulled more strongly toward activities in which it holds a higher comparative advantage. Consequently, the distance between two entity embeddings reflects the similarity of their underlying activity distributions, rather than just the overlap of their active sets. This contrasts with approaches such as the Continuous Projection Space (CPS) [34], where the authors re-parameterize canonical density relatedness (Eq. 4) by substituting the proximity values of the co-occurrence network with the output of a predictive model. In our case, both the entity and activity embeddings are unsupervised representations of the proximity network that also underlies relatedness. That is, we extend relatedness into embedding space by treating an activity’s first-order embedding as the target and an entity’s portfolio, expressed as its second-order embedding, as the origin. This now allows us to use these first-order and second-order representations to translate Economic Complexity measures into the shared embedding space. Each of these measures quantifies an entity’s or activity’s position in and trajectory through the embedding space. The measures are the fit between an entity and a target activity (capability alignment and gap), the concentration of active entities around an activity (sector alignment), the spread of an entity’s portfolio across the embedding space (portfolio entropy), and the trajectory of an entity between periods (displacement). We will introduce each of them in turn.

**Capability Alignment and Gap:** Relatedness as defined in Eq. 4 represents a measure of how close a target activity is to the set of activities an entity is already specialized in. Using first- and second-order embeddings, we can express the same notion geometrically by comparing an entity’s aggregate position with an activity. We measure orientation (cosine similarity) and distance (Euclidean distance) in embedding space. Similar measures have been constructed in the economic literature to compare textual representations of firms using cosine similarity [69] as well as the alignment of a firm representation with an industry prototype [70].

Taking the entity embedding ${\tilde{\vec{e}}}_{u}$ as the origin and the target activity’s embedding ${\tilde{\vec{e}}}_{a}$ as the prototype, we compute their orientational alignment as the cosine similarity:


$$\begin{array}{c} \text{sim}(u,a)=\cos({\tilde{\vec{e}}}_{u},{\tilde{\vec{e}}}_{a})=\frac{{\tilde{\vec{e}}}_{u}\cdot {\tilde{\vec{e}}}_{a}}{\left\|{\tilde{\vec{e}}}_{u}\|\;\|{\tilde{\vec{e}}}_{a}\right\|} \end{array}$$ (6)

where ${\tilde{\vec{e}}}_{u}$ corresponds to an entity embedding and ${\tilde{\vec{e}}}_{a}$ to the activity embedding. Since cosine similarity captures only the angle between an entity’s embedding and the embedding of a target activity, an entity might be aligned with a target activity, while remaining far away from it. We therefore use Euclidean distance to quantify how far apart the two embeddings are. This measure has been used at the firm-level [71,72] to compare firm embeddings. A lower value of $d_{u,a}$ implies that entity *u* is structurally closer to target activity *a*.


$$\begin{array}{c} d_{u,a}=\|{\tilde{\vec{e}}}_{u}-{\tilde{\vec{e}}}_{a}{\|}_{2} \end{array}$$ (7)

**Sector Alignment:** The average alignment of entity embeddings with the embedding of a specific sector can be understood as an analog of *ubiquity*, in that closer average similarities with the activity embedding indicate a denser concentration of knowledge regarding a specific sector. The idea we borrow here is that of *intra-similarity* [73,74] from information retrieval, where a set of items is compared against a focal item. At the firm, level this has been done for patents awarded to firms with a forward- and backward-looking set [75] as well as, closely related to our approach, representations of firms in a specific sector [76]. In that sense, we compute the cosine similarity between all entities active in activity *a* and the focal embedding ${\tilde{\vec{e}}}_{a}$ of that activity.


$$\begin{array}{c} s_{a}=\frac{1}{\left|{𝒰}_{a}\right|}\sum_{u\in \;{𝒰}_{a}}\cos({\tilde{\vec{e}}}_{u},{\tilde{\vec{e}}}_{a}) \end{array}$$ (8)

where $\left|{𝒰}_{a}\right|$ is the cardinality of the set of entities ${𝒰}_{a}$ currently active in activity *a*, i.e., entities with $R_{u,a}>0$. High density, i.e., strong alignment between active entities and the sector’s embedding, may reflect differences in the stability or evolution of knowledge bases across entities.

**Portfolio Entropy** The concept of *diversity* is commonly used in economic complexity to characterize an entity’s portfolio of activities. In diversification research, entropy measures are extensively used to characterize firm [77,78] as well as country [79,80] and region capability profiles [81–83] over taxonomies. However, these measures are computed over the discrete taxonomies, which means that structurally related but taxonomically independent categories are treated as distinct. An entity with activities in structurally related activities may thus appear diversified, where the capabilities are in fact concentrated. To capture diversity given this structure, we partition the activity embeddings into clusters to address the partial redundancy of the taxonomic structure. More specifically, we use the K-Means algorithm to partition activity embeddings into *K* clusters, where *K* is chosen by the silhouette score. Let $\left\{{𝐜}_{1},{𝐜}_{2},...,{𝐜}_{K}\right\}$ represent the set of cluster centroids derived from K-Means. For a given entity *u*, the projection of its embedding ${\tilde{\vec{e}}}_{u}$ onto each centroid ${𝐜}_{k}$ is calculated as:


$$\begin{array}{c} {\vec{q}}_{u,k}=\left(\frac{{\tilde{\vec{e}}}_{u}\cdot {𝐜}_{k}}{\|{𝐜}_{k}{\|}^{2}}\right){𝐜}_{k},\;k=1,...,K \end{array}$$ (9)

where ${\tilde{\vec{e}}}_{u}\cdot {𝐜}_{k}$ is the dot product of the entity embedding and the *k*-th centroid, and $\left\|{𝐜}_{k}\right\|$ is the Euclidean norm of the centroid. The norm of each projection, $\left\|{\vec{q}}_{u,k}\right\|$, is the relative contribution of the *k*-th cluster to the embedding of entity *u*.


$$\begin{array}{c} p_{u,k}=\frac{\left\|{\vec{q}}_{u,k}\right\|}{\sum_{j=1}^{K}\|{\vec{q}}_{u,j}\|} \end{array}$$ (10)

The entropy $H_{u}$ of the portfolio of entity *u* is defined as:


$$\begin{array}{c} H_{u}=-\sum_{k=1}^{K}p_{u,k}\log p_{u,k} \end{array}$$ (11)

where $p_{u,k}$ is the normalized contribution of the *k*-th cluster to the embedding of entity *u*. This measure captures the diversity of the entity’s activities, with higher entropy indicating a more evenly distributed portfolio across clusters.

**Displacement:** While the embedding space measures mentioned above serve as supplementary constructs that are modeled on prototypes from Economic Complexity, the temporal component is indispensable for our understanding of diversification processes. Studying diachronic changes is a common approach in computational linguistics [67], only that here, we use Euclidean distance instead of cosine similarity as the metric of choice, since it registers the repositioning of entities in the activity space, even if the overall sectoral direction remains similar. To this end, we compute the Euclidean distance between two entity embeddings in successive time periods.


$$\begin{array}{c} \Delta_{u}^{\left(t\right)}=\|{\tilde{\vec{e}}}_{u}^{\left(t\right)}-{\tilde{\vec{e}}}_{u}^{\left(t-1\right)}{\|}_{2} \end{array}$$ (12)

As entities evolve over time, a higher level of displacement indicates a reconfiguration of specializations, whereas lower levels would correspond to a more stable set of activities.

### 2.4. Latent space analysis

In the previous section, we introduced embedding space features constructed on top of the proximity network. While traditional measures of economic complexity, such as relatedness, ubiquity, and diversity, may capture lower-order structural information, embeddings may preserve higher-order structural information about the network. Analyzing this latent space helps us understand how it complements traditional measures.

For each dataset, we generate 10 distinct embedding spaces, capping the dimensionality at 150 for procurement and 600 for international trade. This corresponds to roughly half the average number of nodes in each network over time (300 for procurement and 1,200 for trade). This ensures that the embeddings are dense enough to capture meaningful latent relationships, while still achieving a significant dimensionality reduction of the underlying proximity network. In Tables S6 Table and S7 Table of S1 File, we provide an overview of the generation parameters used for each of the embedding spaces.

After constructing entity embeddings and computing their latent features, we proceed to an analysis of the clustering behavior of firms and countries. We use the HDBSCAN [84] and UMAP [85] algorithms for clustering and visualization. HDBSCAN is particularly suited for this task as it can identify clusters of varying shapes and densities. By characterizing these clusters, we can demonstrate that the clusters firms occupy in the embedding space are modular and specialized, whereas countries aggregate around broader, productive knowledge. The hyperparameter grid for the clustering step is provided in S1 Table and the accompanying information in S1 File Section S2. We investigate cluster formation using the Kruskal-Wallis test [86] to identify the distinguishing features of each cluster, using the Benjamini-Hochberg FDR correction [87]. Additionally, we report the effect size ($\eta^{2}$) from the Kruskal-Wallis test. A higher $\eta^{2}$ value indicates that the feature is a more significant separator of clusters. For both firm-level and country-level clusters, we apply these tests to relatedness, embedding space features, capability alignment, gap, portfolio entropy, and displacement. For country-level data, we expand this using the ECI. Since sector alignment is a taxonomy-level feature, we exclude it from this analysis.

We complement this with a feature-level correlation analysis between embedding-space features and measures derived from economic complexity. To understand the relationship between the capability alignment and gap measures and the “principle of relatedness,” we subject relatedness and the two embedding space measures to a quadrant analysis with the aim of quantifying how high capability alignment/low capability gap contributes to diversification outcomes by comparing odds ratios for successful diversification outcomes in the presence of low/high (by means of a median-split) relatedness.

### 2.5. Predictive models

Beyond the theoretical implications of using latent representations of firms and countries, we benchmark all features on a predictive task. The target variable of the prediction task is the RS value of an entity *u* in activity *a* at time t+δ.


$$\begin{array}{c} R_{u,a,t+\delta }=g(x_{t};\theta ) \end{array}$$ (13)

where $R_{u,a,t+\delta }$ represents the predicted specialization value for entity *u* in activity *a* at time t+δ. ${𝐱}_{t}$ denotes the feature vector in time *t*, which incorporates both entity-specific and network-derived attributes (a full list of features is provided in Tables 1 and Table 2). Data dic*t*ionaries for these two feature sets can be consulted in Tables S14 Table, S15 Table and S16 Table of S1 File.

**Table 2:** Feature table for international trade data. For each feature, the Q1-Q3 range is reported. For embedding space features, a standard deviation is provided for each generation parameter set. “HHI” refers here to the Herfindahl-Hirschman Index.

Feature Type	Range (Q1, Q3)	Features
Network Measures	[0.8479, 0.9471]	Degree Centrality
	[0.6045, 0.7295]	Clustering Coefficient
	[5.7156, 5.7864]	Triangle Count (Log10)
	[0.0, 0.0001]	Betweenness Centrality
	[0.0281, 0.0317]	Eigenvector Centrality
Embedding Space	0.3992 ± 0.0841	Capability Alignment
	2.3022 ± 1.3083	Capability Gap
	0.4347 ± 0.1344	Displacement
	0.3669 ± 0.0906	Portfolio Entropy
	0.2466 ± 0.0404	Sectoral Alignment
Country-level	[0.0317, 0.2005]	Product HHI
	[0.0715, 0.1761]	Trading Partner HHI
	[30.8, 41.9]	Inequality (Gini Index)
	[1.6194, 7.142]	Inflation Rate (%)
	[9.614, 11.0617]	GDP (current US$) (Log10)
	[5.8507, 7.3172]	Population (total) (Log10)
	[3.0371, 5.3614]	Education Spending (% of GDP)
	[-0.7634, 0.6731]	Government Quality
	[11.447, 49.9144]	Medium/High-Tech Exports (% of manufactured exports)
	[21.11, 2750.26]	Scientific Articles (count)
	[5.6318, 66.5]	Internet Usage (% of population)
	[6.7235, 16.0498]	Manufacturing Value Added (% of GDP)
Economic Complexity	[0.0568, 0.1977]	Relatedness
	[68, 235]	Diversity
	[14,27]	Ubiquity
	[-0.7591, 0.6777]	Economic Complexity Index (ECI)
	[-0.7187, 0.7732]	Product Complexity Index (PCI)

For public procurement, we set δ=3; for international trade, we set δ=5. *g* is a predictive model with hyperparameters θ that are adjusted using temporal cross-validation. For the hyperparameter selection process, we used a randomized search that included both hyperparameters per ML model and key hyperparameters of the Node2Vec model (dim, walk_len, walk_num, p, q, context). For each of the 10 embedding space configurations (Tables S6 and S7 in S1 File), we evaluated 25 hyperparameter combinations for the re*g*ression models due to their higher computational costs and 50 for the classification models. The combinations with the best validation-fold RMSE (regression) or F1 (classification) were chosen to refit the respective model on the full training set.

Since the datasets in question have a panel-like structure, we evaluated ML models using a moving time-series cross-validation approach. For public procurement data, the years 2009–2018 form the training/validation set, and the years 2019–2024 form the test set. In the case of the international trade data set, the training/validation data stretches from 1998 to 2014, and the test data from 2015 to 2023. Since we use the World Bank dataset as auxiliary variables, and some of these variables have missing values, we perform iterative imputation [88] within each cross-validation fold to avoid temporal leakage. Based on the number of observations, this corresponds to an approximate 75%/25%-split in both cases.

To determine whether specialization prediction is best modeled as regression or classification, we train models for both tasks. In the latter case, we binarize the RS by setting it equal to 1 if $R_{u,a}\geq 1$. The RMSE (Root Mean Squared Error) was chosen as the evaluation metric for the regression case, and the F1-score for the classification case. To compare the performance of the regression and classification models, we transform the regression scores, only considering those entities that have not achieved an RS at *t*.

We employ a model suite with ML models of varying complexity, including simpler models such as linear and regularized regression, as well as more complex tree-based models such as decision trees [89], random forests [90], and XGBoost [91]. Finally, we also included a neural model, the Multilayer Perceptron (MLP), as available in the scikit-learn library [92].

## 3. Results

We organize the results around a single comparative question: when does diversification prediction depend primarily on local overlap (captured by relatedness), and when does broader structural-position information (captured by embedding-derived features) add signal? The central empirical result is that this depends on both the prediction target (entry/non-entry vs specialization magnitude) and the level of aggregation (countries vs firms). We first characterize the relation between embedding-derived and canonical measures, then evaluate predictive performance and boundary conditions across tasks and domains, and finally use feature attribution to compare the contribution of local and structural-position predictors.

### 3.1. Structural information captured by embedding spaces

#### 3.1.1. Correlation structure and complementarity with canonical measures.

We first assess whether embedding-derived features encode information that is redundant with, or complementary to, canonical measures from economic complexity. To this end, we compute correlations between embedding-space features and traditional descriptors, including ubiquity, diversity (number of RS), and relatedness in both domains, as well as ECI and PCI in the trade domain (summarized in S3 Fig. and S4 Fig. of S1 File).

In general, the correlation structure indicates partial overlap rather than equivalence between the two feature classes. Measures associated with relatedness – capacity alignment and capability gap – show only weak associations with the traditional relatedness measure (approximately −0.1 to −0.2 for capability gap, and ≈0.15 to 0.25 for capability alignment across configurations). This is consistent with their intended role as embedding-based proximity descriptors related to, but not direct reparameterizations of, the canonical relatedness construct.

Portfolio entropy is weakly positively associated with diversity (approximately ≈0.25), while displacement is moderately negatively associated with diversity (approximately ≈−0.25) in the trade domain. Diversity (number of RS) also exhibits stronger associations with embedding-derived features in the international trade domain than in the procurement domain (approximately ≈−0.1). These patterns suggest that embedding-based descriptors capture aspects of portfolio structure and movement that cannot be reduced to portfolio size alone.

Associations with ubiquity differ across levels of aggregation. In the procurement domain, sector alignment and ubiquity show stronger negative correlations (approximately ≈−0.6 to −0.5), whereas in the trade domain, the association is weaker and can be positive (approximately ≈0.2). This indicates that the relation between embedding-based alignment and activity prevalence is domain-dependent and likely reflects differences in the structural organization of firm-level versus country-level capability systems.

In the trade domain, correlations between embedding-derived features and complexity indices are generally limited or unstable across configurations and time. The association between ECI and capability alignment is initially strong (declining from approximately ≈0.7 to ≈0.4 over time), while the capability gap shows a more stable negative association. Displacement is also moderately negatively correlated with ECI (approximately ≈−0.4). Taken together, these results indicate that embedding-derived features are neither orthogonal to nor interchangeable with canonical complexity measures; instead, they encode partially overlapping structural information, motivating an explicit test of their incremental contribution in downstream diversification prediction tasks.

#### 3.1.2. Embedding-space organization differs across firms and countries.

We next examine whether entity embeddings recover distinct structural organization across levels of aggregation, and whether these patterns are consistent with known differences between firm-level and country-level capability systems. Previous work has shown that country–activity networks tend to exhibit nested organization, whereas firm–activity networks are more modular [56]. We therefore analyze clustering structure in the entity embedding spaces and compare the variables that most strongly separate clusters across domains and time.

Table 3 summarizes the first five embedding configurations for each domain, including silhouette scores, number of clusters, and the embedding-space variables with the largest Kruskal–Wallis effect sizes ($\eta^{2}$). A full listing of effect sizes and additional configurations is provided in S8 Table to S13 Table in S1 File. For the trade domain, we also report Kruskal–Wallis tests assessing whether ECI differs across embedding-based clusters. We do not report an analogous complexity index for firms, as there is no standard counterpart at that level of aggregation.

**Table 3:** Cluster Analysis by dataset (Configs 1–5). Reported per dataset are the silhouette scores and the number of clusters. International trade metrics compare 1999/2023 periods while procurement metrics compare 2012/2024 periods. The values in parentheses for top separators represent $\eta^{2}$ effect sizes from Kruskal-Wallis tests for embedding space features. Kruskal *H* represents the test results for whether country ECI distinguishes between clusters.

Config ID	Metrics	Embedding Space Separators
**International Trade**		
1	Silhouette: 0.8131/0.8065	Alignment (0.2961)
	Kruskal *H*: 40.8222*** / 20.5570**	Portfolio Entropy (0.3734)
	N° clusters: 7 / 6	
2	Silhouette: 0.7738/0.7536	Capability Gap (0.2789)
	Kruskal *H*: 21.3124*** / 19.0510*	Portfolio Entropy (0.3182)
	N° clusters: 5 / 10	
3	Silhouette: 0.7733/0.7861	Alignment (0.3682)
	Kruskal *H*: 40.6244*** / 29.4145*	Alignment (0.3588)
	N° clusters: 7 / 14	
4	Silhouette: 0.7847/0.8022	Capability Gap (0.3696)
	Kruskal *H*: 35.0359*** / 5.5355	Portfolio Entropy (0.1130)
	N° clusters: 7 / 3	
5	Silhouette: 0.7723/0.8727	Capability Gap (0.1372)
	Kruskal *H*: 25.0309*** / 5.7756*	Portfolio Entropy (0.1327)
	N° clusters: 5 / 2	
**Procurement**		
1	Silhouette: 0.6065/0.6269	Portfolio Entropy (0.1700)
	N° clusters: 28/35	Portfolio Entropy (0.4468)
2	Silhouette: 0.6280/0.7018	Portfolio Entropy (0.0839)
	N° clusters: 42/58	Portfolio Entropy (0.5060)
3	Silhouette: 0.5905/0.6573	Portfolio Entropy (0.0691)
	N° clusters: 34/69	Portfolio Entropy (0.5113)
4	Silhouette: 0.6095/0.6639	Portfolio Entropy (0.1409)
	N° clusters: 42/49	Portfolio Entropy (0.5355)
5	Silhouette: 0.6082/0.6793	Portfolio Entropy (0.1355)
	N° clusters: 31/48	Portfolio Entropy (0.5792)

**Significance Levels (Benjamini-Hochberg):** * p < 0.05; ** p < 0.01; *** p < 0.001.

Across configurations, the clustering results differ systematically between domains. Firm embeddings in the procurement domain form a larger number of clusters with lower-to-moderate silhouette scores relative to the trade domain (Table 3), consistent with a more segmented and modular organization of firm capability portfolios. By contrast, country embeddings in the trade domain form fewer clusters with higher silhouette scores, consistent with broader organization in capability space. These differences are also reflected in the variables that separate clusters: in procurement, portfolio entropy is the dominant separator across configurations and time, whereas in trade, cluster separation is more often associated with capability alignment/gap and, in several configurations, portfolio entropy.

The temporal evolution of cluster separation also differs across domains. In procurement, the effect size of portfolio entropy as a cluster separator increases markedly between 2012 and 2024 across the reported configurations (e.g., from $\eta^{2}\approx 0.07$–0.17 in 2012 to $\eta^{2}\approx 0.45$–0.58 in 2024; Table 3), indicating stronger segmentation of firms by embedding-based portfolio composition over time. In the trade domain, ECI and average relatedness become less discriminative of embedding-based cluster membership between 1999 and 2023 (see S9 Table and S10 Table in S1 File), with declines in both statistical significance and effect size across several configurations. This pattern indicates a weakening correspondence between traditional complexity/relatedness indicators and the cluster structure induced by the embedding space over time.

Fig 2 illustrates these differences using representative embedding configurations with approximately balanced Node2Vec walk parameters (p/q≃1). In the procurement domain (Fig 2A–B), entity embeddings reorganize into increasingly segmented clusters between 2012 and 2024 (e.g., 34 vs. 69 clusters in the illustrated configuration), and the largest clusters correspond to sectorally distinct capability profiles with limited overlap (e.g., construction, medical supplies, IT services). In the trade domain (Fig 2C–D), embedding-based country clusters remain interpretable, but the visual separation by ECI becomes weaker in the later period, consistent with the declining discriminative role of ECI reported above.

**Fig 2:**
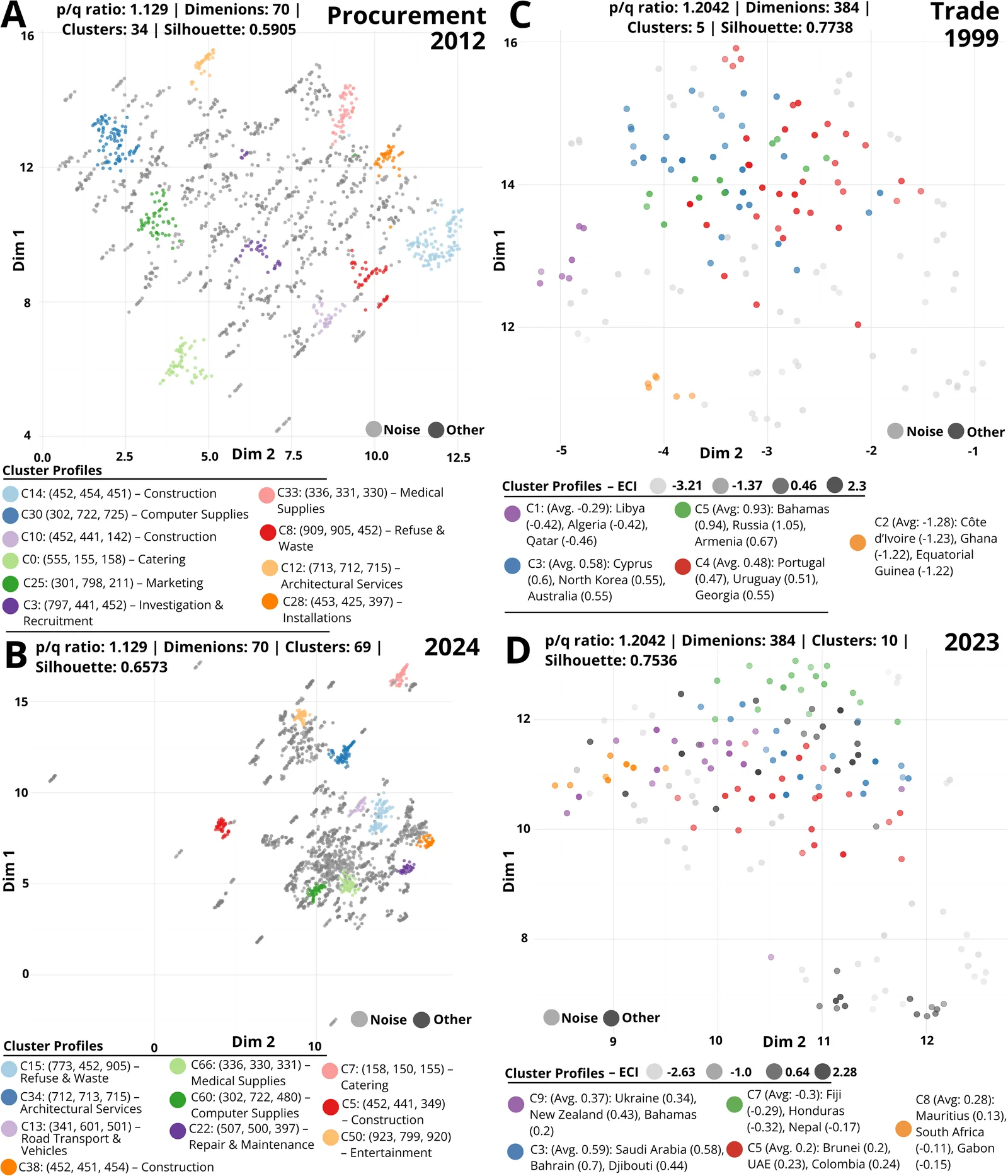
Clustering of Entities (Firms/Countries) in Embedding Space (p/q≃1). Per panel, the silhouette score, the *p*/*q* ratio, and the embedding dimensionality are reported. The chart shows the clusters for embeddings that had a balanced (p/q≃1) ratio. Panels A–B represent the embedding space for the public procurement domain (2012/2024) and panels C–D the international trade domain (1999–2023). In panels C–D, point opacity corresponds to each country’s ECI. For public procurement, the 10 largest clusters are highlighted in color, for international trade, the largest 5.

These results show that entity embeddings recover domain-specific structural organization that transcends a single canonical measure. In particular, the stronger modular segmentation of firm embeddings and the broader, more weakly ECI-stratified organization of country embeddings provide a structural basis for expecting differences in the predictive contribution of embedding-derived features across scales.

### 3.2. Predictive evaluation of diversification: entry events versus specialization magnitude

We next evaluate whether the structural information captured by embedding-derived features translates into improved prediction of diversification outcomes, and whether this depends on the prediction target and level of aggregation. We distinguish between two tasks: classification, which captures entry events, and regression, which captures variation in the magnitude of specialization. This distinction is central because the two tasks need not be driven by the same signals, and because persistence can dominate entry prediction in some settings, limiting the scope for gains from richer representations.

#### 3.2.1. Baseline comparisons and task asymmetry across scales.

Table 4 reports RMSE scores for the regression task and the ROC-AUC and F1, precision, recall and Average Precision metrics for the classification task, in both the procurement and trade domains. We include two baselines for comparison: persistence and a random predictor.

**Table 4:** Comparison of baseline and advanced models for regression and classification tasks across Trade and Procurement domains. Models are ordered from simpler to more complex. Baseline models are marked in italics. The best model for RMSE and F1 is highlighted, and the best for Precision/Recall/ROC-AUC and Average Precision (AP) is underlined. For ROC-AUC and AP the random baselines are the by-construction rates, i.e., 0.5 for ROC-AUC and prevalence for AP.

Domain	Test RMSE	Test F1	Test Precision	Test Recall	Test ROC-AUC	Test AP	Model
**International Trade**							
**Regression**	**20.7366**	**0.2790**	0.2006	0.4580	0.7870	0.1924	*Persistence (Baseline)*
	39.2799	0.0418	0.0385	0.0457	0.5000	0.0374	*Random (Baseline)*
	24.9442	0.0779	0.0419	0.5590	0.5446	0.0418	Linear Regression
	24.9442	0.0779	0.0419	0.5589	0.5446	0.0418	Ridge
	24.9192	0.0794	0.0425	0.6108	0.5535	0.0433	Lasso
	23.2051	0.1067	0.0589	0.5655	0.6596	0.0576	Decision Tree
	21.8164	0.1074	0.0583	0.6788	0.6583	0.0560	Random Forest
	21.5278	0.1289	0.0739	0.5029	0.7043	0.0703	XGBoost
	22.6283	0.0721	0.0374	1.0000	0.5001	0.0374	MLP
**Classification**		**0.2790**	0.2006	0.4580	0.7870	0.1924	*Persistence (Baseline)*
		0.0418	0.0385	0.0457	0.5000	0.0374	*Random (Baseline)*
		0.1305	0.0843	0.2884	0.6926	0.0723	Logistic Regression
		0.1374	0.0871	0.3251	0.6949	0.0753	Decision Tree
		0.0004	0.5385	0.0002	0.7375	0.1001	Random Forest
		0.1363	0.1368	0.1359	0.7394	0.0982	XGBoost
		0.1367	0.1068	0.1896	0.7192	0.0839	MLP
**Procurement**							
**Regression**	37.2930	0.0481	0.1974	0.0274	0.5125	0.0275	*Persistence (Baseline)*
	33.5045	0.0182	0.0177	0.0188	0.5000	0.0187	*Random (Baseline)*
	25.5801	0.1314	0.0710	0.8786	0.7108	0.0623	Linear Regression
	25.5801	0.1314	0.0710	0.8786	0.7108	0.0623	Ridge
	25.5803	0.1316	0.0712	0.8837	0.7106	0.0623	Lasso
	24.9463	0.1671	0.0958	0.6510	0.6416	0.0389	Decision Tree
	26.6634	0.1382	0.0745	0.9595	0.7361	0.0368	Random Forest
	24.5075	**0.2879**	0.1737	0.8399	0.8499	0.0894	XGBoost
	**24.3030**	0.1809	0.1030	0.7398	0.7143	0.0434	MLP
**Classification**		0.0481	0.1974	0.0274	0.5125	0.0275	*Persistence (Baseline)*
		0.0182	0.0177	0.0188	0.5000	0.0187	*Random (Baseline)*
		0.2534	0.1850	0.4021	0.8928	0.1646	Logistic Regression
		0.2523	0.2246	0.2878	0.8790	0.1722	Decision Tree
		0.0506	0.5379	0.0266	0.9036	0.2089	Random Forest
		0.2742	0.2706	0.2780	0.9072	0.2104	XGBoost
		**0.2787**	0.2235	0.3700	0.9037	0.2031	MLP

For comparability across task formulations, we also convert regression-based RS predictions to binary scores and report classification metrics on those outputs.

A first result is a clear asymmetry across levels of aggregation. In the procurement domain (firm level), learned models substantially outperform both baselines across both task formulations. In regression, all supervised models reduce RMSE relative to persistence, and several models also improve downstream classification performance when regression outputs are thresholded. In classification, the strongest models (XGBoost and MLP) achieve markedly higher F1 scores than persistence, indicating that a predictive signal exists beyond simple temporal carryover at the firm level.

By contrast, in the trade domain (country-level), persistence remains difficult to outperform, especially for entry-oriented prediction. Across both regression and classification formulations, no model exceeds the persistence baseline simultaneously on the main summary metrics. Some models improve isolated metrics (e.g., precision or recall), but these gains do not translate into better overall predictive performance. This result should not be read as evidence against the usefulness of richer representations; rather, it identifies a boundary condition in which temporal persistence already captures a large share of the predictable variation, leaving limited room for additional gains from higher-capacity models and feature sets.

A second result is a task asymmetry that is consistent across domains. RS classification (entry prediction) and RS regression (magnitude prediction) are not equivalent forecasting problems. In the procurement domain, models that perform well in regression do not necessarily perform well in classification, and regression outputs converted to binary outcomes can, in some cases, yield competitive or higher F1 scores than models trained directly for classification. This pattern indicates that modeling continuous variation in RS may retain information that is discarded when prediction is framed only as entry/non-entry.

In the trade domain, the same distinction is analytically important even when persistence remains dominant. The inability of more flexible models to outperform persistence in entry prediction suggests that country-level activation events are highly path-dependent at the considered prediction horizon. This makes country-level entry prediction a stringent benchmark and a useful contrast case for interpreting where embedding-derived features are most likely to add value: not necessarily in persistence-dominated entry events, but in tasks and settings where first-order temporal carryover and local overlap are less sufficient.

The class imbalance in the case of the classification task (only 3.7% in the trade and 1.9% in procurement) leads to the necessity of nuance regarding the interpretation of results. In the trade domain, ML models clear the 0.5 baseline of the ROC-AUC metric. However, metrics sensitive to prevalence show that many machine learning models tend to over-predict diversification events, resulting in comparatively high recall and low precision relative to realized entry rates. This pattern is consistent with outcome sparsity in diversification prediction, and reinforces the importance of evaluating models jointly across multiple metrics rather than relying on a single metric.

In addition to modeling diversification as entry classification and RS magnitude prediction, we have also evaluated these models in a ranking system on a pooled and per-entity basis (Table S16 in S1 File). For the evaluation of the regression models as ranking instruments, we ranked by predicted RS magnitude for activity with $R_{u,a}<1$. The results mirror the asymmetries observed in the main results. In the trade domain, persistence outperforms the other models, with only XGBoost and the Random Forest coming close on the pooled measures (precision@100 of 0.4100 and 0.4000, respectively, against persistence’s 0.4600). The gap becomes wider for the per-entity evaluation, where persistence reaches precision@5 = 0.3153 while the best ML model (the Random Forest) attains only 0.1750. This strengthens the path dependence argument already raised in the discussion of classification results. For procurement, the ML models clearly overcome the baseline results, with XGBoost and the Random Forest clearly above the baseline scores (precision@100 of 0.6900 and 0.6400 versus 0.3900; per-entity recall@50 of 0.8220 versus 0.1142. Here, the models’ performance reflects learned structure that is consistent with the results observed for the procurement domain.

We report hyperparameter settings for all models in S2 Table–S5 Table in S1 File. Differences across domains and tasks are consistent with the distinct statistical structure of the prediction problems, including sparsity in classification outcomes and different preferred embedding dimensionalities in regression versus classification settings.

#### 3.2.2. Conditional complementarity of embedding-derived features under low relatedness.

The baseline comparisons above establish that predictive difficulty differs sharply across tasks and levels of aggregation, with country-level entry prediction being particularly persistence-dominated. We therefore evaluate the contribution of embedding-derived features more specifically: not only by overall model performance, but also by testing whether they provide additional signal when the canonical relatedness measure is low.

To assess this, we examine diversification success in low-relatedness settings using a quadrant analysis (S5 Fig). Specifically, we compare the odds of successful diversification across high versus low values of capability alignment and capability gap, conditional on low relatedness. For the capability gap, a low gap is the favorable condition in the odds ratio analysis. This design isolates cases in which local overlap, as captured by relatedness, is weak, thereby providing a direct test of whether embedding-derived features provide additional structural signal beyond first-order proximity. These odds-ratio comparisons are used to assess conditional predictive complementarity under low-relatedness regimes and should not be interpreted as causal estimates.

The results indicate scale-dependent patterns. In international trade, both embedding-space measures are associated with a moderate positive lift under low relatedness (odds ratios: high capability alignment ≈1.15–1.26; low capability gap ≈1.12–1.22). This pattern is consistent with embedding-derived features that capture additional structural information about feasible diversification paths beyond what local overlap alone represents. In the procurement domain, the pattern is more heterogeneous. Capability alignment remains positively associated with diversification success under low relatedness (odds ratios ≈1.03–1.19), but a lower capability gap is not consistently associated with higher success and is often negatively associated (odds ratios ≈0.63–1.02). This asymmetry indicates that, at the firm level, some successful diversification events occur from embedding-space positions that are not locally close to the target activity, consistent with the more modular and specialized organization of firm capability portfolios observed in Section 3.1.2.

Together, these results refine the role of embedding-derived features in the prediction of diversification. They do not uniformly improve prediction across all settings and do not replace relatedness as a first-order predictor. Instead, they provide complementary signals in structurally specific regimes–-particularly when diversification occurs under weak local relatedness and when broader structural position in capability space may carry information not captured by local overlap alone.

### 3.3. Feature attribution across tasks and scales

#### 3.3.1. SHAP analysis of local versus structural-position predictors.

To examine which signals drive model predictions across tasks and levels of aggregation, we compute feature attributions using SHAP (Shapley Additive Explanations) [50] for the best-performing model in each domain-task combination. Fig 3 summarizes mean absolute SHAP values, allowing comparison of the relative contribution of canonical economic complexity measures and embedding-derived features across prediction settings. We use SHAP here as a feature-attribution tool for model interpretation, not as evidence of causal effects. Because panel A differs from the remaining panels in model class, we additionally report the corresponding MLP-based SHAP analysis in S6 Fig in S1 File.

**Fig 3:**
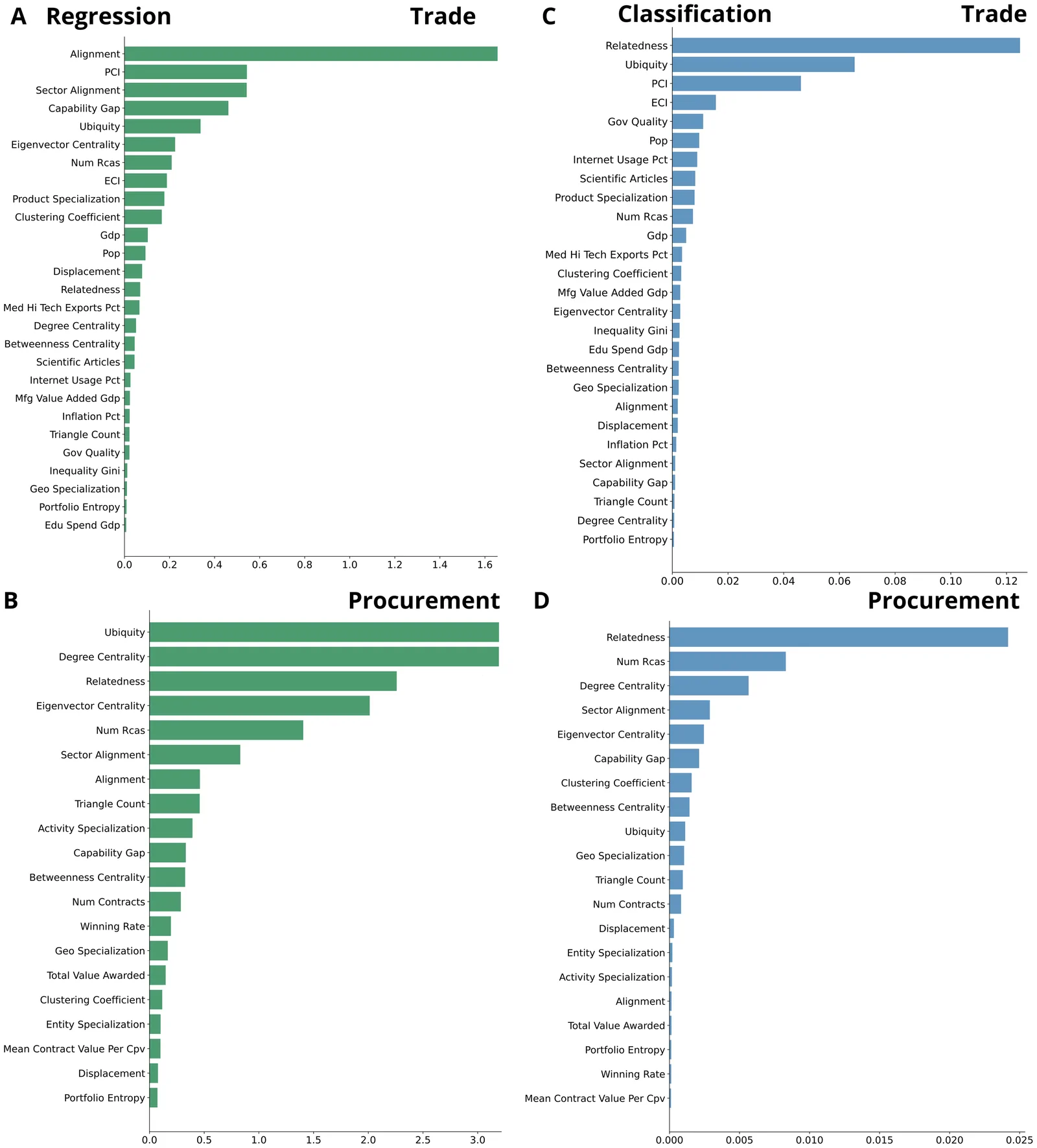
Mean absolute SHAP values for the best-performing models by domain and prediction task. Features are ordered by importance within each panel. The figure contrasts the contributions of canonical local predictors (e.g., relatedness) and embedding-derived/network-based features across RS classification (entry prediction) and RS regression (magnitude prediction). Panel A uses XGBoost and panels B–D use MLP models (see S6 Fig. in S1 File for the corresponding MLP-based attribution).

The SHAP results show a clear task asymmetry. In classification models (entry prediction), the traditional relatedness measure is the dominant predictor in both domains (Fig. 3C–D), with substantially larger mean absolute SHAP values than most other variables. This is consistent with the role of relatedness as a first-order predictor of diversification events and aligns with the persistence-dominated nature of entry prediction identified in Section 3.2.1, especially at the country level.

In regression models (RS magnitude prediction), feature importance is more distributed and varies by level of aggregation. In the trade domain (Fig 3A), capability alignment is the top-ranked predictor by a large margin, with sector alignment and capability gap also ranking highly, while relatedness contributes comparatively less. In the procurement domain (Fig 3B), the largest contributions come from ubiquity and degree centrality, with relatedness also ranking highly; embedding-derived variables (including sector alignment, alignment, and capability gap) contribute additional signal but are not uniformly dominant. This indicates that RS magnitude prediction depends on a broader set of factors, including local compatibility, target-activity network position, and portfolio-level structure.

In the trade domain, we also find a notable role for the PCI measure in entry prediction, both in the classification and regression tasks. Although the overall Spearman correlation between PCI and its SHAP contribution is generally negative (classification −0.4160; regression −0.6930), a look at the PCI tertiles for the two tasks adds nuance. For the regression task, we find a weakening Spearman correlation between the PCI and its contribution to the predicted RS ($\rho^{1}$ =−0.4061; $\rho^{2}$ = −0.3370; $\rho^{3}$ = −0.1742). In the classification task, an initial weakly positive correlation ($\rho^{1}$ = 0.2802) then changes sign at higher complexity ($\rho^{2}$ = −0.6153; $\rho^{3}$ = −0.4482), potentially indicating that among the least complex activities, a higher PCI is associated with a higher probability of entry, while for more complex activities it is associated with a predicted lower probability of entry.

The attribution patterns also differ by level of aggregation, mirroring the structural analyses in Section 3.1. In the procurement domain, SHAP rankings place greater weight on activity-level prevalence and network-position descriptors, consistent with the stronger modular segmentation observed in firm embedding spaces. In the trade domain, the greater relative importance of capability alignment in regression is consistent with a broader system-level organization in which structural-position information may carry a signal not captured by local overlap alone.

These results do not imply that embedding-derived features replace canonical measures. Rather, they clarify how the contribution of local overlap and structural-position information depends on the prediction task and the level of aggregation. Relatedness remains central for entry prediction, while embedding-derived features become more informative in specific settings–-most clearly in trade RS regression and in low-relatedness diversification regimes–-where local overlap alone provides an incomplete description of diversification structure.

### 3.4. Ablation study

We performed an ablation study to understand which of the feature sets contribute to the two tasks in the two domains, respectively. To this end, we divided the feature sets into three parts, i.e., EC features, embedding-derived features, and baseline (country- and firm-specific) features. Additionally, we isolate relatedness as a feature and test it independently of the EC feature set. In Table 5, we summarize the ablations of the feature-set by RMSE for the regression task and F1, recall, precision as well as ROC-AUC and Average Precision (AP) for the classification task per domain. Since across tasks and domains the XGBoost model has the most consistent performance, we elected to summarize here the ablation results using this model, while providing full result tables for all models in S18 Table–S26 Table in S1 File.

**Table 5:** Ablations by feature set. Performance metrics are based on XGBoost model. Two ablation families are shown per task. *Baseline-augmented*: the reference set (baseline features only, i.e., country- and firm-specific features) augmented with one feature set each. *Baseline* is the unaugmented baseline and *Full* is the complete feature set from 4. The EC feature set bundles relatedness and as such, the + EC row also includes the relatedness feature. *Standalone*: a single feature group in isolation, with the baseline features excluded. Bold marks the best configuration per column within each ablation case and task block. The acronym *EDF* denotes the embedding-derived feature set.

	Trade						Procurement					
	RMSE	F1	Prec	Rec	ROC-AUC	AP	RMSE	F1	Prec	Rec	ROC-AUC	AP
*Regression*												
Baseline	24.9327	0.0958	0.0525	0.5476	0.6161	0.0501	26.0790	0.0536	0.0279	0.6995	0.6609	0.0314
+ EC	24.7759	0.1252	0.0715	0.5054	0.6963	0.0673	25.1942	0.1493	0.0832	0.7219	**0.8751**	**0.1170**
+ EDF	22.9236	0.1061	0.0598	0.4669	0.6438	0.0550	24.8917	0.0524	0.0278	0.4521	0.6108	0.0272
+ Relatedness	24.9394	0.0995	0.0547	**0.5526**	0.6330	0.0521	25.6477	0.1256	0.0685	0.7556	0.8551	0.0991
Full	**21.5278**	**0.1289**	**0.0739**	0.5029	**0.7043**	**0.0703**	**24.5075**	**0.2879**	**0.1737**	**0.8399**	0.8499	0.0894
EC-only	24.8514	**0.1179**	**0.0658**	0.5706	**0.6845**	**0.0643**	25.6097	**0.1439**	**0.0804**	0.6876	0.8619	0.0985
EDF	**23.3326**	0.0865	0.0471	0.5315	0.5774	0.0444	**25.1148**	0.0456	0.0245	0.3317	0.5607	0.0237
Relatedness-only	24.9692	0.0870	0.0462	**0.7484**	0.5754	0.0427	25.9010	0.1093	0.0585	**0.8289**	**0.8667**	**0.1274**
*Classification*												
Baseline		0.1203	0.0811	**0.2330**	0.6768	0.0671		0.1390	0.1151	0.1755	0.7878	0.0789
+ EC		**0.1462**	0.1184	0.1908	0.7350	0.0930		**0.2802**	0.2332	0.3509	0.9068	0.2063
+ EDF		0.0873	0.0842	0.0907	0.6772	0.0664		0.1431	0.0981	0.2646	0.7936	0.0802
+ Relatedness		0.0960	0.0922	0.1002	0.6862	0.0697		0.2695	0.2186	**0.3515**	0.9024	0.1938
Full		0.1363	**0.1368**	0.1359	**0.7394**	**0.0982**		0.2742	**0.2706**	0.2780	**0.9072**	**0.2104**
EC-only		**0.1260**	**0.1269**	0.1251	**0.7275**	**0.0912**		**0.2621**	**0.2177**	**0.3294**	**0.9001**	**0.1865**
EDF		0.1109	0.0816	**0.1731**	0.6506	0.0636		0.0784	0.0510	0.1701	0.7104	0.0433
Relatedness-only		0.1049	0.0862	0.1338	0.6664	0.0659		0.2127	0.1583	0.3240	0.8680	0.1279

The ablation results show that each of the feature sets contributes in different ways to the regression and classification tasks across the two domains. EC and relatedness represent most of the signal in entry prediction tasks for both the procurement and trade domains. As an isolated feature set, EC-only leads on the classification metrics, reaching an F1 of 0.2621 (ROC-AUC 0.9001) in procurement. For trade, the performance gap is narrower and the EC feature set achieves only a marginally better F1 (0.1260) than the embedding-derived feature set (0.1109) or even relatedness itself (0.1049). In procurement entry classification, relatedness reaches an F1 of 0.2127 with a ROC-AUC of 0.8680, recovering much of the EC set’s performance (F1 0.2621, ROC-AUC 0.9001) by itself.

The signal provided by the embedding-derived features is largely concentrated in the magnitude-prediction task. The feature set configurations in the trade domain that have the lowest RMSE all include embeddings, the full set (21.5278), baseline-plus-embeddings (22.9236), and embeddings-only (23.3326), while the addition of EC features have no tangible impact on the RMSE metric. Similarly, in procurement, where the three lowest-RMSE configurations are again the embedding-bearing ones (the full set at 24.5075, baseline-plus-embeddings at 24.8917, and embeddings-only at 25.1148). The reverse holds for entry prediction, where in procurement entry classification the embedding-derived feature set in isolation is weakest (F1 0.0784, ROC-AUC 0.7104) and they provide only marginal lift over the domain-specific baseline features.

The entry-ranking task (S36 Table; full results in S1 File, S27 Table–S35 Table in S1 File) reinforces this pattern. The EC feature set (including relatedness) are the main driver of ranking performance, whereas the embedding-derived feature set contributes little. The domain difference between procurement and trade shows some differentiation within the EC feature set: In the procurement domain, relatedness alone accounts for nearly all of the signal. As a standalone feature it slightly exceeds or approximates the performance o the full EC set (recall@50 of 0.7859 vs. 0.7395 in magnitude ranking; 0.8118 vs. 0.7926 in the classifier ranking). In the trade domain, the EC feature set is the best-performing standalone feature set and outperforms relatedness alone (P@1000 of 0.0920 vs. 0.0180, P@10 of 0.0884 vs. 0.0441, recall@50 of 0.1050 vs. 0.0552)). Nevertheless, absolute ranking performance in the trade domain remains low, mirroring the overall domain results in Table 4.

Overall, the ablation study further qualifies the results of the SHAP analysis. The three feature sets encode complementary information, with entry prediction driven by relatedness in procurement and by the broader EC feature set in the trade domain, while the embedding-derived features contribute primarily to magnitude prediction.

## 4. Discussion

The principle of relatedness remains a central empirical regularity in diversification research, and our results are consistent with that role across domains and levels of aggregation. At the same time, our findings show that the same underlying proximity structure can be represented in ways that capture different aspects of diversification. In particular, embedding-derived features provide information about structural position in capability space that is not imputable to first-order co-occurrence alone.

The central empirical finding is not that embedding-derived features outperform relatedness, but that the diversification signal is organized differently across prediction targets and levels of aggregation. In entry prediction (RS classification), relatedness remains the dominant predictor, particularly at the country level, where persistence is the strongest benchmark. In RS regression, by contrast, attribution rankings are less concentrated, and embedding-derived measures — especially capability alignment in trade — become more prominent. This pattern indicates that entry and RS magnitude are related but non-equivalent prediction problems, and that they rely on different combinations of local-overlap and structural-position information.

A second result concerns differences across scales. The embedding-space analyses reveal patterns consistent with distinct organization in firm- and country-level systems, consistent with prior evidence that firm–activity networks are more modular while country–activity networks tend to be more nested [56]. In our data, firm embeddings show stronger sectoral segmentation, whereas country embeddings occupy broader configurations in capability space. These differences help interpret why embedding-derived features contribute differently across domains: in more segmented systems, diversification may involve movements across specialized regions of capability space, whereas in broader systems, positional structure may be more informative for variation in RS magnitude than for binary entry events.

The quadrant analysis further refines this interpretation. In low-relatedness settings, embedding-derived measures (particularly capability alignment) are associated with differences in diversification success, whereas the capability-gap specification shows domain-dependent behavior. This indicates that embedding features can provide additional signal in regimes where local overlap is weak. Importantly, this is a conditional and regime-specific contribution: it does not imply uniform gains across all prediction tasks, nor does it weaken the central role of relatedness as a first-order predictor of diversification events.

These results also clarify the role of embedding-based representations in economic complexity applications. Their value lies less in replacing canonical constructs and more in making structural-position information beyond pairwise overlap available for theory-grounded feature design and comparative evaluation. In this sense, the contribution of embeddings is both methodological and substantive: they provide a tractable way to operationalize positional structure in capability networks while preserving links to interpretable concepts such as alignment, gap, and portfolio composition. These attribution patterns, however, should be interpreted as model-based importance rankings rather than evidence of causal mechanisms.

The implications for applied use should therefore be stated carefully. In policy-oriented or strategic settings, relatedness remains an appropriate benchmark for identifying nearby diversification opportunities [93–95]. Embedding-derived features may be useful as complementary indicators when the objective is to evaluate variation in feasibility among low-relatedness opportunities, compare structurally distinct diversification paths, or identify cases in which local overlap is an incomplete descriptor of capability position. Whether these features improve decision-making in practice remains an empirical question that requires domain-specific validation.

This study has several limitations. First, although the embedding-derived features are theory-informed, they remain less directly interpretable than canonical measures such as relatedness, diversity, and ubiquity. Second, our framework inherits the strengths and limitations of RS-based representations: capabilities that are emerging but not yet reflected in observed comparative advantage may be underrepresented. Third, the approach is intentionally agnostic to specific mesoscopic properties of proximity networks (e.g., core–periphery, ring-like structures, or other domain-specific morphologies) [19,54,63]; this may improve portability across domains but may also limit performance or interpretability in settings where such structures are central to diversification dynamics.

Several extensions follow from these limitations. Future work could evaluate embedding spaces using alternative downstream tasks (e.g., recommendation-style ranking of diversification opportunities based directly on entity embeddings instead of embedding-derived features), compare node embedding methods that emphasize different structural notions (e.g., topology preservation versus role similarity), and test whether domain-specific constraints or network morphology can be integrated into feature construction without sacrificing interpretability. A further direction is to assess how embedding-derived features perform under alternative definitions of activity entry beyond RS thresholds, particularly in settings where emerging capabilities are only weakly expressed in observed outcomes.

## Supporting information

S1 File Supplementary Information. This file contains six figures (S1–S6) and thirty-six tables (S1–S36). S1 Fig.–S2 Fig. reproduce Fig. 2 for different *p*/*q* configurations. S3 Fig. and S4 Fig. are referred to in Section 3.1.1 to elucidate the correlation analysis. S5 Fig. contains the odds-ratio figure discussed in Section 3.2.2. S6 Fig. shows the SHAP values for the international trade regression model (MLP) for Fig. 3. S1 Table contains the hyperparameter grids for international trade and public procurement clustering. S2 Table – S5 Table provide summaries of the ML model and embedding space parameters of the models compared in Table 4. S6 Table and S7 Table contain the embedding generation hyperparameters for international trade and public procurement, respectively. S8 Table – S13 Table provide additional cluster analyses. S14 Table – S16 Table contain feature dictionaries. Network and embedding features are computed identically for both datasets (firms/CPV groups for procurement, countries/HS4 products for trade); these tables define them and link features to their sources. S17 Table contains the ranking evaluation of ML models for the full feature set. S18 Table – S26 Table contain result tables for the ablation study (Section 3.4) concerning the entry prediction and specialization magnitude tasks (main results in Table 4). S27 Table – S35 Table contain the ranking evaluation of ML models as part of the ablation study. S36 Table provides the ablation summary (Table 5 for the main task) for the ranking task.
(PDF)

## References

[1] Hidalgo CA. Economic complexity theory and applications. Nat Rev Phys. 2021;3(2):92–113. doi: 10.1038/s42254-020-00275-1

[2] Hidalgo CA, Hausmann R. The building blocks of economic complexity. Proc Natl Acad Sci U S A. 2009;106(26):10570–5. doi: 10.1073/pnas.0900943106 19549871 PMC2705545

[3] Hidalgo CA, Klinger B, Barabási A-L, Hausmann R. The product space conditions the development of nations. Science. 2007;317(5837):482–7. doi: 10.1126/science.1144581 17656717

[4] Hidalgo CA, Balland PA, Boschma R, Delgado M, Feldman M, Frenken K. The principle of relatedness. The Principle of Relatedness. Springer International Publishing. 2018. p. 451–7. doi: 10.1007/978-3-319-96661-8_46

[5] Pinheiro FL, Hartmann D, Boschma R, Hidalgo CA. The time and frequency of unrelated diversification. Research Policy. 2022;51(8):104323. doi: 10.1016/j.respol.2021.104323

[6] Content J, Frenken K. Related variety and economic development: a literature review. European Planning Studies. 2016;24(12):2097–112. doi: 10.1080/09654313.2016.1246517

[7] Boschma R, Capone G. Relatedness and diversification in the European Union (EU-27) and European Neighbourhood Policy countries. Environment and Planning C: Government and Policy. 2015;34(4):617–37. doi: 10.1177/0263774x15614729

[8] Li Y, Neffke FMH. Evaluating the principle of relatedness: Estimation, drivers and implications for policy. Research Policy. 2024;53(3):104952. doi: 10.1016/j.respol.2024.104952

[9] Li Y, Rigby D. Relatedness, complexity, and economic growth in Chinese cities. International Regional Science Review. 2022;46(1):3–37. doi: 10.1177/01600176221082308

[10] Rigby DL. Technological Relatedness and Knowledge Space: Entry and Exit of US Cities from Patent Classes. Regional Studies. 2013;49(11):1922–37. doi: 10.1080/00343404.2013.854878

[11] Balland P-A, Boschma R, Crespo J, Rigby DL. Smart specialization policy in the European Union: relatedness, knowledge complexity and regional diversification. Regional Studies. 2018;53(9):1252–68. doi: 10.1080/00343404.2018.1437900

[12] Boschma R. Relatedness as driver of regional diversification: a research agenda. Regional Studies. 2016;51(3):351–64. doi: 10.1080/00343404.2016.1254767

[13] Boschma R, Minondo A, Navarro M. The Emergence of New Industries at the Regional Level in Spain: A Proximity Approach Based on Product Relatedness. Economic Geography. 2012;89(1):29–51. doi: 10.1111/j.1944-8287.2012.01170.x

[14] Bruno M, Saracco F, Squartini T, Dueñas M. Colombian export capabilities: building the firms-products network. Entropy. 2018;20(10):785. doi: 10.3390/e20100785 PMC7512347 33265873

[15] Poncet S, de Waldemar FS. Product Relatedness and Firm Exports in China. The World Bank Economic Review. 2013;29(3):579–605. doi: 10.1093/wber/lht037

[16] Neffke F, Henning M. Skill relatedness and firm diversification. Strategic Management Journal. 2012;34(3):297–316. doi: 10.1002/smj.2014

[17] Kim SH, Jun B, Lee J-D. Technological relatedness: how do firms diversify their technology?. Scientometrics. 2023;128(9):4901–31. doi: 10.1007/s11192-023-04775-6

[18] Breschi S, Lissoni F, Malerba F. Knowledge-relatedness in firm technological diversification. Research Policy. 2003;32(1):69–87. doi: 10.1016/s0048-7333(02)00004-5

[19] Guevara MR, Hartmann D, Aristarán M, Mendoza M, Hidalgo CA. The research space: using career paths to predict the evolution of the research output of individuals, institutions, and nations. Scientometrics. 2016;109(3):1695–709. doi: 10.1007/s11192-016-2125-9

[20] Juhász S, Wachs J, Kaminski J, Hidalgo CA. The software complexity of nations. Research Policy. 2026;55(3):105422. doi: 10.1016/j.respol.2026.105422

[21] Miao L, Murray D, Jung W-S, Larivière V, Sugimoto CR, Ahn Y-Y. The latent structure of global scientific development. Nat Hum Behav. 2022;6(9):1206–17. doi: 10.1038/s41562-022-01367-x 35654964

[22] Alshamsi A, Pinheiro FL, Hidalgo CA. Optimal diversification strategies in the networks of related products and of related research areas. Nat Commun. 2018;9(1):1328. doi: 10.1038/s41467-018-03740-9 29626192 PMC5889401

[23] Klement B, Strambach S. How do new music genres emerge? Diversification processes in symbolic knowledge bases. Regional Studies. 2019;53(10):1447–58. doi: 10.1080/00343404.2019.1580817

[24] Zhu S, Li R. Economic complexity, human capital and economic growth: empirical research based on cross-country panel data. Applied Economics. 2016;49(38):3815–28. doi: 10.1080/00036846.2016.1270413

[25] Stojkoski V, Utkovski Z, Kocarev L. The Impact of Services on Economic Complexity: Service Sophistication as Route for Economic Growth. PLoS One. 2016;11(8):e0161633. doi: 10.1371/journal.pone.0161633 27560133 PMC4999235

[26] Romero JP, Gramkow C. Economic complexity and greenhouse gas emissions. World Development. 2021;139:105317. doi: 10.1016/j.worlddev.2020.105317

[27] Zhu S, Yu C, He C. Export structures, income inequality and urban-rural divide in China. Applied Geography. 2020;115:102150. doi: 10.1016/j.apgeog.2020.102150

[28] Cohen WM, Levinthal DA. Absorptive capacity: A new perspective on learning and innovation. Administrative Science Quarterly. 1990;35(1):128. doi: 10.2307/2393553

[29] Teece DJ, Rumelt R, Dosi G, Winter S. Understanding corporate coherence. Journal of Economic Behavior & Organization. 1994;23(1):1–30. doi: 10.1016/0167-2681(94)90094-9

[30] Lüthge A. The concept of relatedness in diversification research: review and synthesis. Rev Manag Sci. 2018;14(1):1–35. doi: 10.1007/s11846-018-0293-0

[31] Albora G, Pietronero L, Tacchella A, Zaccaria A. Product progression: a machine learning approach to forecasting industrial upgrading. Sci Rep. 2023;13(1):1481. doi: 10.1038/s41598-023-28179-x 36707529 PMC9880377

[32] Albora G, Zaccaria A. Machine Learning to Assess Relatedness: The Advantage of Using Firm‐Level Data. Complexity. 2022;2022(1). doi: 10.1155/2022/2095048

[33] Straccamore M, Pietronero L, Zaccaria A. Which will be your firm’s next technology? Comparison between machine learning and network-based algorithms. J Phys Complex. 2022;3(3):035002. doi: 10.1088/2632-072x/ac768d

[34] Tacchella A, Zaccaria A, Miccheli M, Pietronero L. Relatedness in the era of machine learning. Chaos, Solitons & Fractals. 2023;176:114071. doi: 10.1016/j.chaos.2023.114071

[35] Gnecco G, Nutarelli F, Riccaboni M. A machine learning approach to economic complexity based on matrix completion. Sci Rep. 2022;12(1):9639. doi: 10.1038/s41598-022-13206-0 35689004 PMC9187690

[36] Nutarelli F, Edet S, Gnecco G, Riccaboni M. Predicting the technological complexity of global cities based on unsupervised and supervised machine learning methods. Journal of Economic Behavior & Organization. 2025;234:107011. doi: 10.1016/j.jebo.2025.107011

[37] Huang Y, Xu S, Lü L, Zaccaria A, Mariani MS. Uncovering key predictors of high-growth firms via explainable machine learning. arXiv. 2024. 10.48550/ARXIV.2408.09149

[38] Choi J, Menon A, Tabakovic H. Using machine learning to revisit the diversification–performance relationship. Strategic Management Journal. 2021;42(9):1632–61. doi: 10.1002/smj.3317

[39] Sturm NF, Candia C, Damásio B, Pinheiro FL. Augmenting Firm Diversification Behavior Prediction with Graph Embeddings. In: Cherifi H, et al., editors. Complex Networks 2025. Springer Nature Switzerland AG. 2026. p. 1–12. doi: 10.1007/978-3-032-16723-1_10

[40] Gnecco G, Nutarelli F, Riccaboni M. Matrix completion of world trade: An analysis of interpretability through Shapley values. World Economy. 2023;46(9):2707–31. doi: 10.1111/twec.13457

[41] Hamilton WL, Ying R, Leskovec J. Representation Learning on Graphs: Methods and Applications. In: 2017. 10.48550/ARXIV.1709.05584

[42] Suominen A, Toivanen H, Seppänen M. Firms’ knowledge profiles: Mapping patent data with unsupervised learning. Technological Forecasting and Social Change. 2017;115:131–42. doi: 10.1016/j.techfore.2016.09.028

[43] Murray D, Yoon J, Kojaku S, Costas R, Jung W-S, Milojević S, et al. Unsupervised embedding of trajectories captures the latent structure of scientific migration. Proc Natl Acad Sci U S A. 2023;120(52):e2305414120. doi: 10.1073/pnas.2305414120 38134198 PMC10756268

[44] Gurciullo S, Mikhaylov SJ. Detecting policy preferences and dynamics in the UN general debate with neural word embeddings. In: 2017 International Conference on the Frontiers and Advances in Data Science (FADS), 2017. 74–9. 10.1109/fads.2017.8253197

[45] Gezici AHB, Sefer E. PageRank-Based Unsupervised Deep Vertex Representations for Anti-Money Laundering Detection. IEEE Access. 2025;13:196935–50. doi: 10.1109/access.2025.3634197

[46] Van Belle R, Mitrović S, De Weerdt J. Representation learning in graphs for credit card fraud detection. Representation Learning in Graphs for Credit Card Fraud Detection. Springer International Publishing. 2020. p. 32–46. doi: 10.1007/978-3-030-37720-5_3

[47] Wang D, Lin J, Cui P, Jia Q, Wang Z, Fang Y, et al. A Semi-Supervised Graph Attentive Network for Financial Fraud Detection. In: 2019 IEEE International Conference on Data Mining (ICDM), 2019. 598–607. 10.1109/icdm.2019.00070

[48] Singer U, Guy I, Radinsky K. Node Embedding over Temporal Graphs. In: Proceedings of the Twenty-Eighth International Joint Conference on Artificial Intelligence, 2019. 4605–12. 10.24963/ijcai.2019/640

[49] Grover A, Leskovec J. node2vec: Scalable Feature Learning for Networks. KDD. 2016;2016:855–64. doi: 10.1145/2939672.2939754 27853626 PMC5108654

[50] Lundberg S, Lee SI. A Unified Approach to Interpreting Model Predictions. In: 2017. 10.48550/ARXIV.1705.07874v2

[51] Observatory of Economic Complexity. HS6 rev. 1996 (1998 - 2023). https://oec.world/en/resources/bulk-download/international?topic=Trade 2023.

[52] World Bank. World Development Indicators; 2025. Online. Available from: https://data.worldbank.org/

[53] Instituto dos Mercados Públicos, do Imobiliário e da Construção (IMPIC). Portal BASE. https://www.base.gov.pt/Base4/pt/ 2025.

[54] Sturm NF, Candia C, Damásio B, Pinheiro FL. High earnings through firm influence: the role of hierarchical structures in public procurement. EPJ Data Sci. 2025;14(1). doi: 10.1140/epjds/s13688-025-00543-z

[55] Balassa B. Trade liberalisation and “revealed” comparative advantage. Manchester School. 1965;33(2):99–123. doi: 10.1111/j.1467-9957.1965.tb00050.x

[56] Laudati D, Mariani MS, Pietronero L, Zaccaria A. The different structure of economic ecosystems at the scales of companies and countries. J Phys Complex. 2023;4(2):025011. doi: 10.1088/2632-072x/accb35

[57] Pinheiro FL, Balland P-A, Boschma R, Hartmann D. The dark side of the geography of innovation: relatedness, complexity and regional inequality in Europe. Regional Studies. 2022;59(1). doi: 10.1080/00343404.2022.2106362

[58] Boschma R, Martín V, Minondo A. Neighbour regions as the source of new industries. Papers in Regional Science. 2017;96(2):227–46. doi: 10.1111/pirs.12215

[59] Boschma R, Balland PA, Kogler DF. Relatedness and technological change in cities: the rise and fall of technological knowledge in US metropolitan areas from 1981 to 2010. Industrial and Corporate Change. 2014;24(1):223–50. doi: 10.1093/icc/dtu012

[60] Phelps C, Heidl R, Wadhwa A. Knowledge, Networks, and Knowledge Networks: A Review and Research Agenda. Journal of Management. 2012;38(4):1115–66. doi: 10.1177/0149206311432640

[61] Hausmann R, Hidalgo CA. The network structure of economic output. J Econ Growth. 2011;16(4):309–42. doi: 10.1007/s10887-011-9071-4

[62] Juhász S, Broekel T, Boschma R. Explaining the dynamics of relatedness: The role of co‐location and complexity. Papers in Regional Science. 2021;100(1):3–22. doi: 10.1111/pirs.12567

[63] Hidalgo CA, Stojkoski V. The Theory of Economic Complexity. 2025. 10.48550/ARXIV.2506.18829v6

[64] Kudret E, Sefer E. Optimal Reconstruction of Graph Evolution History Under Preferential Attachment Model. IEEE Trans Comput Biol Bioinform. 2026;23(4):1475–86. doi: 10.1109/TCBBIO.2026.3683906 41979950

[65] Mattsson CES. Network growth under opportunistic attachment. Appl Netw Sci. 2025;10(1). doi: 10.1007/s41109-025-00706-x

[66] Lo Turco A, Maggioni D. On firms’ product space evolution: the role of firm and local product relatedness. J Econ Geogr. 2015;16(5):975–1006. doi: 10.1093/jeg/lbv024

[67] Hamilton WL, Leskovec J, Jurafsky D. Diachronic Word Embeddings Reveal Statistical Laws of Semantic Change. In: Proceedings of the 54th Annual Meeting of the Association for Computational Linguistics (Volume 1: Long Papers), 2016. 1489–501. 10.18653/v1/p16-1141

[68] Hurley JR, Cattell RB. The procrustes program: Producing direct rotation to test a hypothesized factor structure. Syst Res. 2007;7(2):258–62. doi: 10.1002/bs.3830070216

[69] Hoberg G, Phillips G. Text-Based Network Industries and Endogenous Product Differentiation. Journal of Political Economy. 2016;124(5):1423–65. doi: 10.1086/688176

[70] Gouvard P, Durand R. Clearing Up Confusion: The effect of outlier similarity on IPO underpricing. Organization Studies. 2024;47(1):157–88. doi: 10.1177/01708406241286287

[71] Jo S, Kim T, Park J, Yu J, Bae H, Chung J. A BERT-based denoising framework for inter-company similarity analysis using 10-K reports. 2025. 10.2139/ssrn.5798353

[72] Gerling C. Company2Vec — German company embeddings based on corporate websites. International Journal of Information Technology & Decision Making. 2023;23(06):2209–43. doi: 10.1142/s0219622023500694

[73] Ethayarajh K. How Contextual are Contextualized Word Representations? Comparing the Geometry of BERT, ELMo, and GPT-2 Embeddings. In: Proceedings of the 2019 Conference on Empirical Methods in Natural Language Processing and the 9th International Joint Conference on Natural Language Processing (EMNLP-IJCNLP), 2019. 55–65. 10.18653/v1/d19-1006

[74] Zhou T, Su R-Q, Liu R-R, Jiang L-L, Wang B-H, Zhang Y-C. Accurate and diverse recommendations via eliminating redundant correlations. New J Phys. 2009;11(12):123008. doi: 10.1088/1367-2630/11/12/123008

[75] Morandi Stagni R, Santaló J. Investors’ attention and the paradox of technologically related diversification: Evidence of stock market mispricing. Strategic Management Journal. 2025;46(10):2432–66. doi: 10.1002/smj.3726

[76] Hoberg G, Phillips G. Conglomerate industry choice and product language. Management Science. 2018;64(8):3735–55. doi: 10.1287/mnsc.2016.2693

[77] Schommer M, Richter A, Karna A. Does the Diversification–Firm Performance Relationship Change Over Time? A Meta‐Analytical Review. J Management Studies. 2018;56(1):270–98. doi: 10.1111/joms.12393

[78] Raghunathan SP. A Refinement of the Entropy Measure of Firm Diversification: Toward Definitional and Computational Accuracy. Journal of Management. 1995;21(5):989–1002. doi: 10.1177/014920639502100510

[79] Teza G, Caraglio M, Stella AL. Entropic measure unveils country competitiveness and product specialization in the World trade web. Sci Rep. 2021;11(1):10189. doi: 10.1038/s41598-021-89519-3 33986366 PMC8119984

[80] Saviotti PP, Frenken K. Export variety and the economic performance of countries. J Evol Econ. 2008;18(2):201–18. doi: 10.1007/s00191-007-0081-5

[81] Rocchetta S, Mina A. Technological coherence and the adaptive resilience of regional economies. Regional Studies. 2019;53(10):1421–34. doi: 10.1080/00343404.2019.1577552

[82] Boschma R, Iammarino S. Related variety, trade linkages, and regional growth in Italy. Economic Geography. 2009;85(3):289–311. doi: 10.1111/j.1944-8287.2009.01034.x

[83] Frenken K, Van Oort F, Verburg T. Related variety, unrelated variety and regional economic growth. Regional Studies. 2007;41(5):685–97. doi: 10.1080/00343400601120296

[84] McInnes L, Healy J, Astels S. hdbscan: Hierarchical density based clustering. JOSS. 2017;2(11):205. doi: 10.21105/joss.00205

[85] McInnes L, Healy J, Melville J. UMAP: Uniform Manifold Approximation and Projection for Dimension Reduction. In: 2018. 10.48550/ARXIV.1802.03426v3

[86] Kruskal WH, Wallis WA. Errata: Use of Ranks in One-Criterion Variance Analysis. Journal of the American Statistical Association. 1953;48(264):907. doi: 10.2307/2281082

[87] Benjamini Y, Hochberg Y. Controlling the False Discovery Rate: A Practical and Powerful Approach to Multiple Testing. Journal of the Royal Statistical Society Series B: Statistical Methodology. 1995;57(1):289–300. doi: 10.1111/j.2517-6161.1995.tb02031.x

[88] Buuren S v, Groothuis-Oudshoorn K. Mice: Multivariate imputation by chained equations in R. Journal of Statistical Software. 2011;45(3). doi: 10.18637/jss.v045.i03

[89] Breiman L, Friedman JH, Olshen RA, Stone CJ. Classification and regression trees. Routledge. 1984. doi: 10.1201/9781315139470

[90] Breiman L. Random Forests. Machine Learning. 2001;45(1):5–32. doi: 10.1023/a:1010933404324

[91] Chen T, Guestrin C. In: Proceedings of the 22nd ACM SIGKDD International Conference on Knowledge Discovery and Data Mining, 2016. 785–94. 10.1145/2939672.2939785

[92] Buitinck L, Louppe G, Blondel M, Pedregosa F, Mueller A, Grisel O. API design for machine learning software: experiences from the scikit-learn project. 2013. 10.48550/ARXIV.1309.0238

[93] Boschma R. Designing Smart Specialization Policy: Relatedness, Unrelatedness, or What?. Oxford University Press. 2025. p. 59–79. doi: 10.1093/oxfordhb/9780198866190.013.0010

[94] Panori A, Kakderi C, Dimitriadis I. Combining technological relatedness and sectoral specialization for improving prioritization in Smart Specialisation. Regional Studies. 2021;56(9):1454–67. doi: 10.1080/00343404.2021.1988552

[95] Conte GL, Mina A, Rocchetta S. Turning Technological Relatedness into Industrial Strategy: The Productivity Effects of Smart Specialization in Europe. Economic Geography. 2025;101(1):28–59. doi: 10.1080/00130095.2025.2456731

