# Correction: The latent structure of diversification from relatedness and network embeddings

**DOI:** 10.1371/journal.pone.0362979

**Published:** 2026-11-13

**Authors:** Niclas Frederic Sturm, Cristian Candia, Bruno Damásio, Flávio L. Pinheiro

There is an error in affiliation 1 for authors Niclas Frederic Sturm, Bruno Damásio and Flávio L. Pinheiro. The correct affiliation 1 is: NOVA Information Management School (NOVA IMS), Universidade NOVA de Lisboa, Campus de Campolide, 1070−312 Lisboa, Portugal.

The Funding statement for this article is incorrect. The correct Funding statement is as follows: NFS, BD, and FLP acknowledge the financial support provided by Fundação para a Ciência e Tecnologia (FCT) Portugal (https://www.fct.pt/) under the projects 2024/07378/IACDC (https://doi.org/10.54499/2024.07378.IACDC), 2024/07601/IACDC (https://doi.org/10.54499/2024.07601.IACDC), UID/04152/2025 – Centro de Investigação em Gestão de Informação (MagIC)/NOVA IMS (https://doi.org/10.54499/UID/04152/2025, 2025-01-01/2028-12-31), and UID/PRR/04152/2025 (https://doi.org/10.54499/UID/PRR/04152/2025, 2025-01-01/2026-06-30). The funding agency did not play any role in the study design, data collection and analysis, decision to publish, or preparation of the manuscript.
