# Supplementary material for: The latent structure of diversification from relatedness and network embeddings
Source: PLoS One. 2026 Oct 22;21(10):e0344958. doi: 10.1371/journal.pone.0344958 (PMC13690220; doi:10.1371/journal.pone.0344958)
Supplement: Supplementary file 1 [file pone.0344958.s001.pdf]

Supporting Information: The latent structure of diversification from relatedness and network embeddings

Niclas Frederic Sturm, Cristian Candia, Bruno Damásio, Flávio L. Pinheiro

Contents

S1 Embeddings Construction and Alignment **3**

S2 Clustering of Entities **5**

S3 Hyperparameter Summary **6**

S4 Node2Vec Generation Parameters **10**

S5 Clustering Analysis for Different p/q ratios **11**

S6 Cluster Statistics (Kruskal-Wallis/Effect Sizes) **13**

S7 Correlations **17**

S8 Odds Ratio Analysis **19**

S9 SHAP Analysis **20**

S10 Data Dictionaries **21**

S11 Ranking evaluation of ML models (Main Set) **23**

S12 Ablation Summaries **27**

S12.1 Classification/Regression 27

S12.1.1 No EC features 27

S12.1.2 No Embedding features 28

S12.1.3 No Relatedness 29

S12.1.4 EC-only 30

S12.1.5 Embedding-derived features only 31

S12.1.6 Relatedness-only 32

S12.1.7 Baseline Features 33

S12.1.8 Baseline Features/Embedding-derived features/Relatedness 34

S12.1.9 Baseline features/relatedness 35

S12.2 Ranking 36

S12.2.1 No EC features . . . 36
S12.2.2 No Embedding features . . . 39
S12.2.3 No Relatedness . . . 42
S12.2.4 EC-only . . . 45
S12.2.5 Embedding-derived features only . . . 48
S12.2.6 Relatedness-only . . . 51
S12.2.7 Baseline Features . . . 54
S12.2.8 Baseline Features/Embedding-derived features/Relatedness . . . 57
S12.2.9 Baseline features/relatedness . . . 60
S12.3 Ranking Ablation Summary . . . 63

S1 Embeddings Construction and Alignment

In this section, we provide additional detail to construction and subsequent alignment of the embedding spaces as well as sparsification procedures. The construction of the embedding space, comprising first-order activity embeddings and second-order entity embeddings aggregated via Eq. (5), has been described in the main text.

In the international trade domain, the proximity network is notably dense, with around 725,000 to 753,000 edges connecting 1,205 to 1,228 activities. Networks in the procurement domain are less dense, with networks comprising 14,000 to 24,000 edges and 289 to 302 activities. This higher edge-to-node ratio in the trade domain increases computational demands and risks noisier embeddings from weaker, less economically meaningful connections. To address these challenges, we apply a domain-specific sparsification approach to the international trade networks. We first construct a Maximum Spanning Tree (MST) to preserve network connectivity. Then, we reintroduce edges with a proximity of at least 0.5. The results of this sparsification strategy account for between 80% and 87% of the edges in the trade networks.

As noted in the main text, the tNodeEmbed framework, proposed by [7], addresses the challenge of learning stable node representations in temporal graphs. A primary issue in training static embeddings on independent snapshots is "stochastic drift" [5]. This phenomenon arises because the loss functions used to train embedding models are typically invariant to orthogonal transformations; consequently, training instances of the embeddings can produce latent spaces that, while being structurally equivalent, are rotated or reflected relative to one another.

The *tNodeEmbed* framework, proposed by [7], addresses the challenge of learning stable node representations in temporal graphs. A primary issue in training static embeddings on independent snapshots is "stochastic drift" [5]. This phenomenon arises because the loss functions used to train embedding models are typically invariant to orthogonal transformations; consequently, training instances of the embeddings can produce latent spaces that, while being structurally equivalent, are rotated or reflected relative to one another.

The methods proposed in the *tNodeEmbed* framework resolve this via a multi-stage process comprising initialization, node-based warm starts, and post-hoc orthogonal *Procrustes* alignment [4]. This alignment phase seeks an orthogonal transformation matrix R that maps the current latent space B into the coordinate system of the previous space A, minimizing the Frobenius norm:

$$\min_{R} \|BR - A\|_F \quad \text{subject to} \quad R^T R = I \tag{1}$$

The alignment is executed iteratively for each time step. The following algorithm describes the functional steps for aligning the embedding space at time t with the established reference frame at $t-1$. A limitation of this approach, naturally, is that it assumes the node embeddings have not dramatically changed between periods. We collect the raw activity embeddings $\vec{e}_a$ of period t as the rows of the snapshot matrix $Z_t \in \mathbb{R}^{|\mathcal{V}_t| \times d}$, and denote by $\tilde{Z}_t$ its aligned counterpart, whose rows are the aligned embeddings $\tilde{\vec{e}}_a$ used throughout the main text.

Algorithm 1 Temporal Embedding Alignment

Require: Aligned reference embeddings $\tilde{Z}_{t-1}$ and raw snapshot embeddings Z_t; Shared activities $\mathcal{V}_{shared} = \mathcal{V}_t \cap \mathcal{V}_{t-1}$

Ensure: Aligned embedding matrix $\tilde{Z}_t$, with rows $\tilde{\vec{e}}_a$

1: Construct $A \in \mathbb{R}^{|\mathcal{V}_{shared}| \times d}$ using embeddings $\tilde{\vec{e}}_a \in \tilde{Z}_{t-1}$ for $a \in \mathcal{V}_{shared}$
2: Construct $B \in \mathbb{R}^{|\mathcal{V}_{shared}| \times d}$ using embeddings $\vec{e}_a \in Z_t$ for $a \in \mathcal{V}_{shared}$
3: Compute cross-correlation matrix: $M \leftarrow A^T B$
4: Decompose M via Singular Value Decomposition: $U, \Sigma, V^T \leftarrow \text{SVD}(M)$
5: Recover optimal rotation matrix: $R \leftarrow V U^T$
6: Apply transformation to the global snapshot: $\tilde{Z}_t \leftarrow Z_t \cdot R$
7: **return** $\tilde{Z}_t$

S2 Clustering of Entities

To map semantic structures across the international trade and public procurement domains, we perform a clustering analysis that couples UMAP dimensionality reduction with HDBSCAN. UMAP here is primarily utilized as a pre-processing step to mitigate the "curse of dimensionality", which often renders density-based clustering difficult for high-dimensional data [1]. It is important to note that manifold learning techniques can introduce structural artifacts or distort global distances [2]; therefore, the projection is treated as a heuristic to highlight local connectivity rather than as a means of perfectly preserving the original topology.

To address these limitations and verify the meaningfulness of the results, we reviewed the resulting clusters and, as described in the main article, the distinctions align with underlying economic variables. Optimal configurations are identified through a grid search, selecting parameter combinations that maximize the silhouette score for non-noise data points (labels $\neq -1$). The grid is shown in Table S1.

It is important to note that the 2D structures used for visual representation in the cluster plots (Fig. 2 of the main article; Fig. S1 and Fig. S2) are generated via a separate UMAP projection (`n_components`=2) to ensure visual clarity, whereas the clustering logic operates on the specific dimensionality reduction targets defined in the hyperparameter grid.

The search space is constructed according to the constraints imposed by the specific density and scale of each dataset, contrasting broader country-level profiles of international trade with granular, firm-level data in public procurement.

Table S1: Hyperparameter grids for international trade and public procurement clustering. In both cases, the silhouette score was used to select the most fitting configuration.

**Parameter**	**International Trade**	**Public Procurement**
UMAP		
`n_neighbors`	$\{2, 4, 6, 8, 10, 12, 14\}$	$\{15, 30, 45, 60\}$
`n_components`	$0.2 \times$ Embedding Dims	$0.1 \times$ Embedding Dims
`metric`	Cosine	Cosine
HDBSCAN		
`min_cluster_size`	$\{5, 8, 10, 12, 15, 18, 20\}$	$\{10, 20, 35, 55, 80\}$
`min_samples`	$\{2, 4, 6, 8, 10, 12\}$	$\{2, 5, 8, 12\}$

S3 Hyperparameter Summary

Table S2: Summary of Classification ML Model Hyperparameters and Embedding Space Parameters (Public Procurement).

**Model Name**	**Hyperparameters**	**Embedding Space Hyperparameters**
Logistic Regression	penalty: l2<br>C: 0.0001	dim: 70<br>walk_len: 35<br>walk_num: 28<br>p: 0.4244<br>q: 0.3760<br>p/q: 1.1288<br>context: 9
Decision Tree	min_samples_split: 300<br>min_samples_leaf: 50<br>max_depth: 10<br>criterion: entropy	dim: 84<br>walk_len: 50<br>walk_num: 19<br>p: 1.1134<br>q: 0.7590<br>p/q: 1.4670<br>context: 4
Random Forest	n_estimators: 300<br>min_samples_leaf: 5<br>max_features: sqrt<br>max_depth: 60<br>criterion: entropy	dim: 124<br>walk_len: 41<br>walk_num: 16<br>p: 1.0358<br>q: 0.5566<br>p/q: 1.8609<br>context: 5
XGBoost	subsample: 0.8<br>scale_pos_weight: 2<br>n_estimators: 900<br>min_child_weight: 5<br>max_depth: 11<br>learning_rate: 0.01<br>colsample_bytree: 0.6	dim: 89<br>walk_len: 50<br>walk_num: 20<br>p: 0.8863<br>q: 1.4477<br>p/q: 0.6122<br>context: 7
MLP	learning_rate_init: 0.0001<br>hidden_layer_sizes: (256, 128, 64)<br>alpha: 0.0791<br>activation: relu	dim: 125<br>walk_len: 20<br>walk_num: 27<br>p: 0.4473<br>q: 0.5524<br>p/q: 0.8098<br>context: 5

Table S3: Summary of Regression ML Model Hyperparameters and Embedding Space Parameters (Public Procurement).

Model Name	Hyperparameters	Embedding Space Hyperparameters
Linear Regression	No hyperparameters	dim: 138<br>walk_len: 55<br>walk_num: 20<br>p: 1.0595<br>q: 1.4700<br>p/q: 0.7207<br>context: 10
Lasso	alpha: 0.00274	dim: 138<br>walk_len: 55<br>walk_num: 20<br>p: 1.0595<br>q: 1.4700<br>p/q: 0.7207<br>context: 10
Ridge	alpha: 1.11e-06	dim: 138<br>walk_len: 55<br>walk_num: 20<br>p: 1.0595<br>q: 1.4700<br>p/q: 0.7207<br>context: 10
Decision Tree	max_depth: 5<br>min_samples_split: 10<br>min_samples_leaf: 50<br>max_features: sqrt	dim: 125<br>walk_len: 20<br>walk_num: 27<br>p: 0.4473<br>q: 0.5524<br>p/q: 0.8098<br>context: 5
Random Forest	n_estimators: 100<br>max_depth: 30<br>min_samples_leaf: 5<br>max_features: sqrt	dim: 78<br>walk_len: 20<br>walk_num: 16<br>p: 1.1460<br>q: 0.8958<br>p/q: 1.2794<br>context: 5
XGBoost	n_estimators: 300<br>learning_rate: 0.05<br>max_depth: 3<br>subsample: 0.8<br>colsample_bytree: 1.0<br>reg_lambda: 100<br>reg_alpha: 0.1	dim: 107<br>walk_len: 75<br>walk_num: 21<br>p: 1.5732<br>q: 0.3943<br>p/q: 3.9895<br>context: 4
MLP	hidden_layer_sizes: [32]<br>alpha: 4.29e-05<br>activation: logistic<br>learning_rate_init: 0.001	dim: 125<br>walk_len: 20<br>walk_num: 27<br>p: 0.4473<br>q: 0.5524<br>p/q: 0.8098<br>context: 5

Table S4: Summary of Classification ML Model Hyperparameters and Embedding Space Parameters (International Trade).

Model Name	Hyperparameters	Embedding Space Hyperparameters
Logistic Regression	penalty: l1<br>C: 0.00689	dim: 200<br>walk_len: 75<br>walk_num: 14<br>p: 0.5541<br>q: 1.0049<br>p/q: 0.5514<br>context: 9
Decision Tree	min_samples_split: 300<br>min_samples_leaf: 50<br>max_depth: 10	dim: 494<br>walk_len: 68<br>walk_num: 25<br>p: 0.5015<br>q: 0.3030<br>p/q: 1.6549<br>context: 14
Random Forest	n_estimators: 500<br>min_samples_leaf: 5<br>max_features: 0.4<br>max_depth: 60	dim: 175<br>walk_len: 55<br>walk_num: 18<br>p: 1.5094<br>q: 1.0975<br>p/q: 1.3753<br>context: 9
XGBoost	n_estimators: 900<br>learning_rate: 0.01<br>max_depth: 11<br>subsample: 0.8<br>colsample_bytree: 0.6<br>min_child_weight: 5<br>scale_pos_weight: 2	dim: 175<br>walk_len: 55<br>walk_num: 18<br>p: 1.5094<br>q: 1.0975<br>p/q: 1.3753<br>context: 9
MLP	hidden_layer_sizes: (64, 64, 64)<br>alpha: 0.00146<br>activation: relu	dim: 314<br>walk_len: 31<br>walk_num: 28<br>p: 1.3204<br>q: 0.3073<br>p/q: 4.2966<br>context: 5

Table S5: Summary of Regression ML Model Hyperparameters and Embedding Space Parameters (International Trade).

Model Name	Hyperparameters	Embedding Space Hyperparameters
Linear Regression	No hyperparameters	dim: 384<br>walk_len: 31<br>walk_num: 11<br>p: 0.9134<br>q: 0.7585<br>p/q: 1.2042<br>context: 9
Lasso	alpha: 0.1290	dim: 384<br>walk_len: 31<br>walk_num: 11<br>p: 0.9134<br>q: 0.7585<br>p/q: 1.2042<br>context: 9
Ridge	alpha: 48.8025	dim: 384<br>walk_len: 31<br>walk_num: 11<br>p: 0.9134<br>q: 0.7585<br>p/q: 1.2042<br>context: 9
Decision Tree	min_samples_split: 2<br>min_samples_leaf: 50<br>max_features: 0.9<br>max_depth: 11	dim: 513<br>walk_len: 72<br>walk_num: 29<br>p: 0.3456<br>q: 1.0626<br>p/q: 0.3252<br>context: 6
Random Forest	n_estimators: 100<br>min_samples_leaf: 5<br>max_features: sqrt<br>max_depth: 10	dim: 164<br>walk_len: 56<br>walk_num: 25<br>p: 1.2582<br>q: 0.3095<br>p/q: 4.0653<br>context: 3
XGBoost	n_estimators: 800<br>learning_rate: 0.05<br>max_depth: 6<br>subsample: 0.6<br>colsample_bytree: 0.7<br>reg_lambda: 100<br>reg_alpha: 0.1	dim: 164<br>walk_len: 56<br>walk_num: 25<br>p: 1.2582<br>q: 0.3095<br>p/q: 4.0653<br>context: 3
MLP	hidden_layer_sizes: (256, 128, 64)<br>alpha: 0.00596<br>activation: relu<br>learning_rate_init: 0.001	dim: 340<br>walk_len: 40<br>walk_num: 13<br>p: 0.7170<br>q: 0.6326<br>p/q: 1.1334<br>context: 10

S4 Node2Vec Generation Parameters

Table S6: Embedding Generation Parameters (International Trade).

Config ID	dim	walk_len	walk_num	p	q	context
1	542	77	15	0.71	1.01	8
2	175	55	18	1.51	1.10	9
3	314	31	28	1.32	0.31	5
4	433	74	18	0.67	1.17	4
5	513	72	29	0.35	1.06	6
6	494	68	25	0.50	0.30	14
7	200	75	14	0.55	1.00	9
8	340	40	13	0.72	0.63	10
9	384	31	11	0.91	0.76	9
10	164	56	25	1.26	0.31	3

Table S7: Embedding Generation Parameters (Public Procurement).

Config ID	dim	walk_len	walk_num	p	q	context
1	73	21	18	0.710	1.211	5
2	138	55	20	1.059	1.470	10
3	70	35	28	0.424	0.376	9
4	125	20	27	0.447	0.552	5
5	107	75	21	1.573	0.394	4
6	73	36	27	1.667	0.555	14
7	84	50	19	1.113	0.759	4
8	124	41	16	1.036	0.557	5
9	78	20	16	1.146	0.896	5
10	89	50	20	0.886	1.448	7

S5 Clustering Analysis for Different p/q ratios

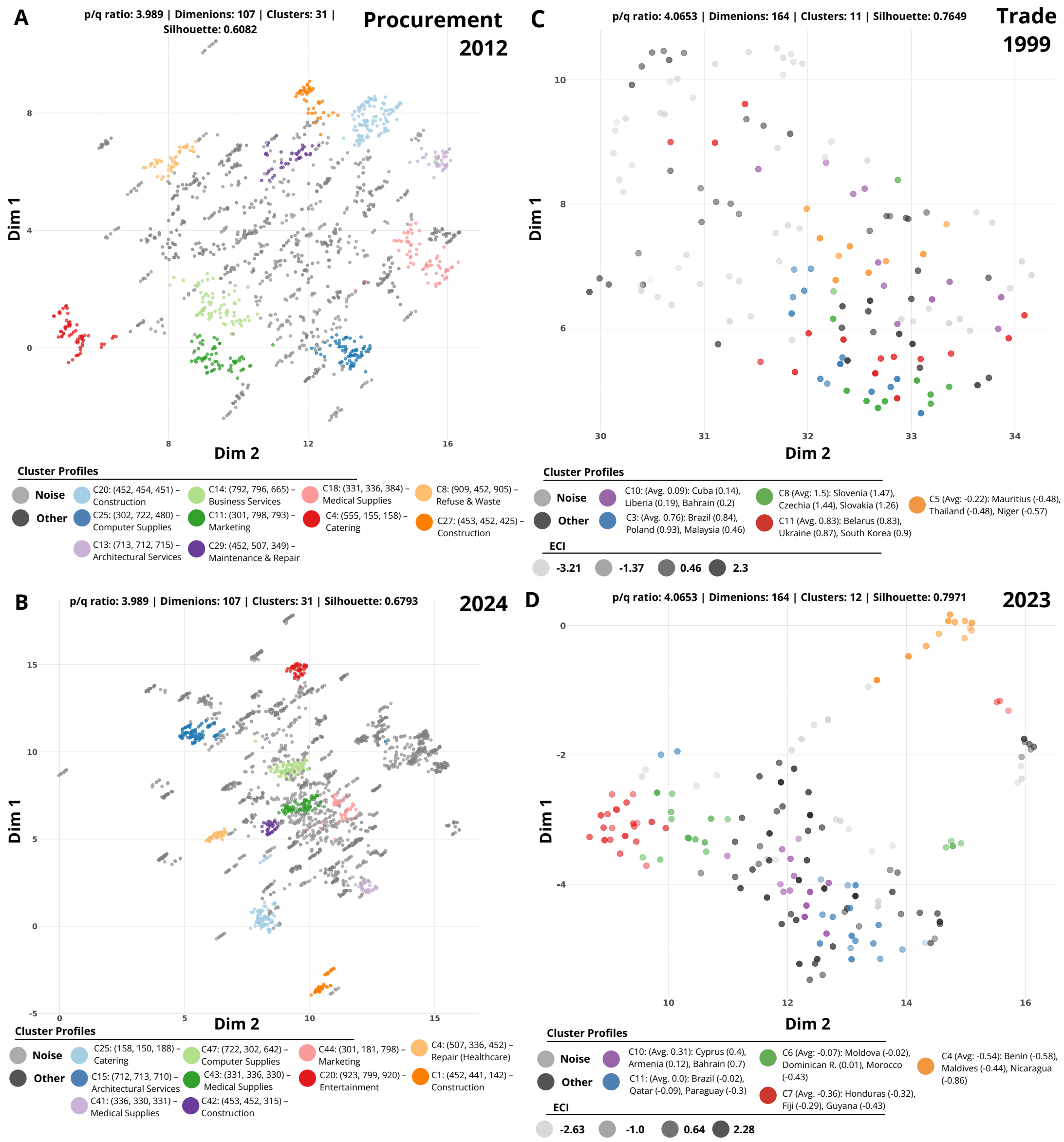

Figure S1: Clustering of Entities (Firms/Countries) in Embedding Space ($p/q > 1$). Per panel, the silhouette score, the p/q ratio, and the embedding dimensionality are reported. The chart shows the clusters for embeddings with a p/q ratio that emphasises local community structures ($p/q \simeq 1$). Panels A–B represent the embedding space for the public procurement domain (2012/2024) and panels C–D the international trade domain (1999–2023). In panels C–D, point opacity corresponds to each country's ECI. For public procurement, the 10 largest clusters are highlighted in colour, for international trade, the largest 5.

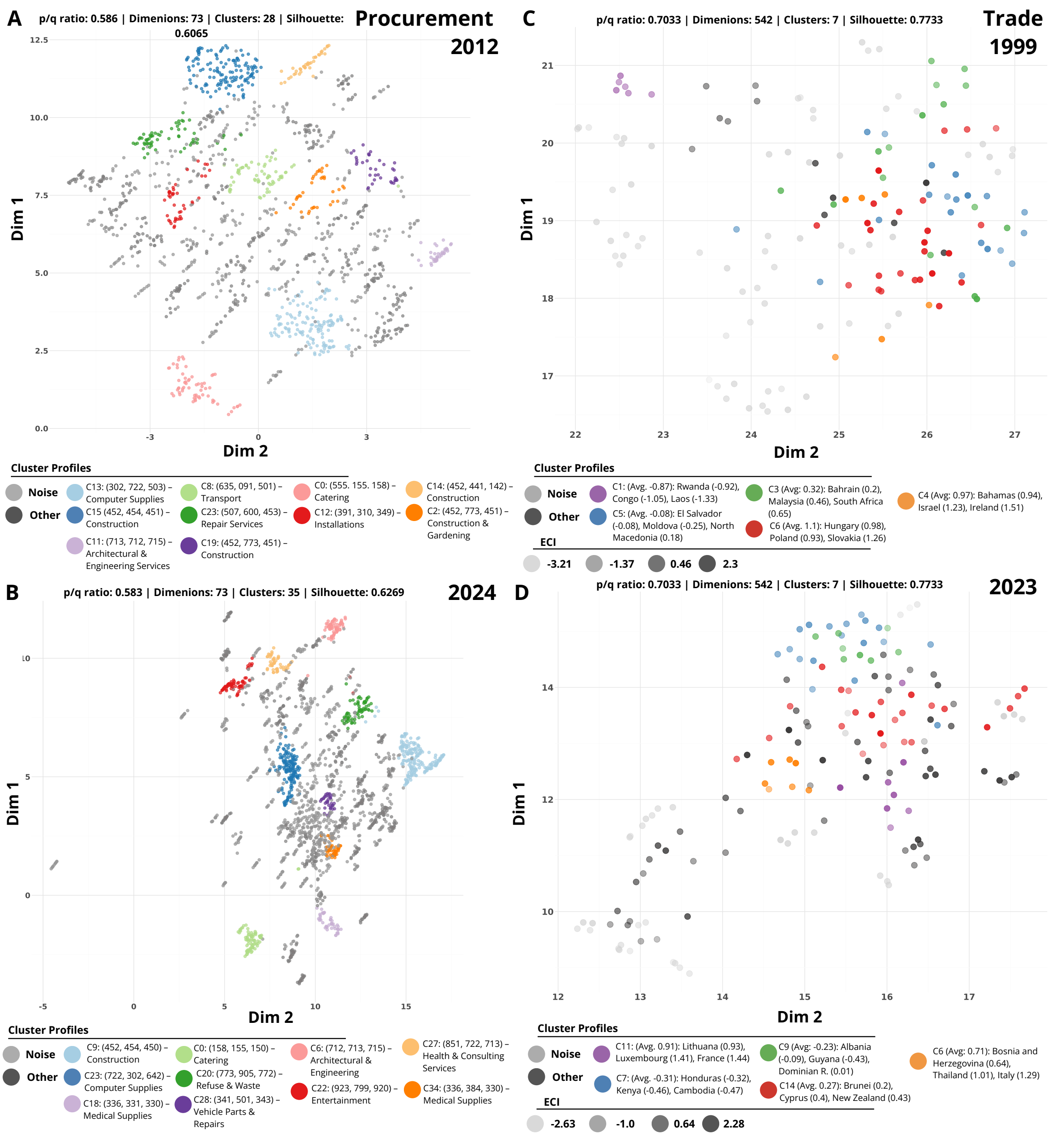

Figure S2: Clustering of Entities (Firms/Countries) in Embedding Space ($p/q < 1$). Per panel, the silhouette score, the p/q ratio, and the embedding dimensionality are reported. The chart shows the clusters for embeddings with a p/q ratio that emphasises the structural topology ($p/q \simeq 1$). Panels A–B represent the embedding space for the public procurement domain (2012/2024) and panels C–D the international trade domain (1999–2023). In panels C–D, point opacity corresponds to each country's ECI. For public procurement, the 10 largest clusters are highlighted in colour, for international trade, the largest 5.

S6 Cluster Statistics (Kruskal-Wallis/Effect Sizes)

Table S8: Cluster Analysis by dataset (Configs 6–10). Reported per dataset are the silhouette scores and the number of clusters. International trade metrics compare 1999/2023 periods while procurement metrics compare 2012/2024 periods. The values in parentheses for top separators represent η^2 effect sizes from Kruskal-Wallis tests for embedding space features. Kruskal H represents the test results for whether country ECI distinguishes between clusters.

Config ID	Metrics	Embedding Space Separators
**International Trade**		
6	Silhouette: 0.8186/0.8379	Capability Gap (0.3934)
	Kruskal H: 43.0548*** / 27.1794**	Capability Gap (0.3728)
	N° clusters: 10 / 10	
7	Silhouette: 0.7649/0.7971	Capability Gap (0.4995)
	Kruskal H: 61.2406*** / 23.7406*	Portfolio Entropy (0.4964)
	N° clusters: 11 / 12	
8	Silhouette: 0.7983/0.8032	Alignment (0.2776)
	Kruskal H: 32.6045*** / 6.0270*	Alignment (0.0184)
	N° clusters: 6 / 2	
9	Silhouette: 0.8055/0.8379	Capability Gap (0.3962)
	Kruskal H: 52.0462*** / 7.3725*	Portfolio Entropy (0.1438)
	N° clusters: 10 / 3	
10	Silhouette: 0.8109/0.8062	Alignment (0.3266)
	Kruskal H: 34.2769*** / 29.1416**	Alignment (0.3831)
	N° clusters: 7 / 13	
**Procurement**		
6	Silhouette: 0.6078/0.6344	Portfolio Entropy (0.0679)
	N° clusters: 24/32	Portfolio Entropy (0.3786)
7	Silhouette: 0.6198/0.6413	Portfolio Entropy (0.0851)
	N° clusters: 32/44	Portfolio Entropy (0.4684)
8	Silhouette: 0.6377/0.6812	Portfolio Entropy (0.1018)
	N° clusters: 34/67	Portfolio Entropy (0.5619)
9	Silhouette: 0.5709/0.6501	Portfolio Entropy (0.0481)
	N° clusters: 17/39	Portfolio Entropy (0.4920)
10	Silhouette: 0.6295/0.6417	Portfolio Entropy (0.0909)
	N° clusters: 36/59	Portfolio Entropy (0.5439)

Significance Levels (Benjamini-Hochberg): * $p < 0.05$; ** $p < 0.01$; *** $p < 0.001$

Table S9: Cluster Separation by ECI (International Trade).

Config ID	1999		2023	
	Kruskal H	η^2	Kruskal H	η^2
1	40.82***	0.4353	20.56**	0.1377
2	21.31***	0.2061	19.05*	0.0948
3	40.62***	0.4439	29.41*	0.1441
4	35.04***	0.4033	5.54	0.0411
5	25.03***	0.3004	5.78*	0.0304
6	43.05***	0.4257	27.18**	0.1567
7	61.24***	0.5757	23.74*	0.1019
8	32.60***	0.3366	6.03*	0.0679
9	52.05***	0.5186	7.37*	0.0680
10	34.28***	0.3821	29.14**	0.1714

Significance Levels (Benjamini-Hochberg): $***\ p < 0.001$, $**\ p < 0.01$, $*\ p < 0.05$

Table S10: Cluster Separation by Relatedness (International Trade).

Config ID	1999		2023	
	Kruskal H	η^2	Kruskal H	η^2
1	25.02***	0.2377	20.66**	0.1386
2	28.99***	0.2975	14.28	0.0498
3	32.47***	0.3394	18.72	0.0441
4	31.37***	0.3523	6.20*	0.0488
5	20.19***	0.2313	4.01*	0.0191
6	35.40***	0.3300	27.21**	0.1569
7	51.06***	0.4614	25.00*	0.1120
8	31.29***	0.3205	3.12	0.0286
9	47.80***	0.4675	3.55	0.0196
10	24.40***	0.2486	25.11*	0.1311

Significance Levels (Benjamini-Hochberg): $***\ p < 0.001$, $**\ p < 0.01$, $*\ p < 0.05$

Table S11: Cluster Separation by Relatedness (Public Procurement).

Config ID	2012		2024	
	Kruskal H	η^2	Kruskal H	η^2
1	269.17***	0.2427	467.97***	0.3587
2	329.30***	0.2643	586.92***	0.3425
3	278.17***	0.2459	605.12***	0.3637
4	313.91***	0.2428	503.74***	0.3006
5	301.17***	0.2573	492.55***	0.3631
6	255.29***	0.2550	461.42***	0.3466
7	295.13***	0.2709	545.63***	0.3405
8	300.14***	0.2671	549.29***	0.3246
9	192.89***	0.1988	538.54***	0.3503
10	296.88***	0.2540	524.46***	0.3644

Significance Levels (Benjamini-Hochberg): $***\ p < 0.001$, $**\ p < 0.01$, $*\ p < 0.05$

Table S12: Cluster separation by embedding space configuration (Configs 1-5). Reported per parameter set and domain are the Kruskal H scores and η^2 for the periods 1999/2023 (International Trade) and 2012/2024 (Public Procurement).

Config ID	Trade (1999/2023)		Procurement (2012/2024)	
	Feature	Kruskal H (η^2)	Feature	Kruskal H (η^2)
1	Alignment	29.69*** / 27.93*** (0.2961/0.2029)	Alignment	633.47*** / 62364.70*** (0.0020/0.1669)
	Portfolio Entropy	16.68* / 47.19*** (0.1335/0.3734)	Portfolio Entropy	50777.36*** / 166882.43*** (0.1700/0.4468)
	Capability Gap	28.02*** / 24.42*** (0.2753/0.1719)	Capability Gap	17976.05*** / 23615.01*** (0.0601/0.0631)
	Displacement	24.37*** / 20.71** (0.2296/0.1391)	Displacement	11757.60*** / 52186.32*** (0.0393/0.1396)
2	Alignment	21.39*** / 39.11*** (0.2070/0.2841)	Alignment	2368.91*** / 60508.72*** (0.0071/0.1256)
	Portfolio Entropy	1.68/ 42.73*** (0.0000/0.3182)	Portfolio Entropy	27710.44*** / 243651.05*** (0.0839/0.5060)
	Capability Gap	27.43*** / 39.02*** (0.2789/0.2832)	Capability Gap	24579.78*** / 23283.54*** (0.0744/0.0482)
	Displacement	15.54** / 24.68** (0.1374/0.1480)	Displacement	12272.86*** / 52620.08*** (0.0371/0.1092)
3	Alignment	34.72*** / 52.39*** (0.3682/0.3588)	Alignment	781.89*** / 73488.25*** (0.0025/0.1583)
	Portfolio Entropy	6.13/ 48.91*** (0.0017/0.3263)	Portfolio Entropy	20768.01*** / 237180.73*** (0.0691/0.5113)
	Capability Gap	30.77*** / 48.60*** (0.3175/0.3234)	Capability Gap	13519.51*** / 31078.55*** (0.0450/0.0669)
	Displacement	26.16*** / 26.30* (0.2585/0.1149)	Displacement	12128.24*** / 88805.63*** (0.0403/0.1914)
4	Alignment	30.33*** / 4.13ns (0.3380/0.0248)	Alignment	1939.13*** / 57314.41*** (0.0056/0.1220)
	Portfolio Entropy	17.79** / 11.72** (0.1637/0.1130)	Portfolio Entropy	47833.37*** / 251448.43*** (0.1409/0.5355)
	Capability Gap	32.61*** / 3.71ns (0.3696/0.0198)	Capability Gap	18295.96*** / 13443.85*** (0.0538/0.0285)
	Displacement	24.85*** / 3.46ns (0.2618/0.0170)	Displacement	16431.57*** / 35677.52*** (0.0483/0.0759)
5	Alignment	10.77* / 16.53*** (0.0967/0.0989)	Alignment	851.22*** / 64602.99*** (0.0026/0.1688)
	Portfolio Entropy	1.99/ 21.83*** (0.0000/0.1327)	Portfolio Entropy	42822.95*** / 221544.16*** (0.1355/0.5792)
	Capability Gap	13.61* / 20.10*** (0.1372/0.1217)	Capability Gap	14774.38*** / 25440.39*** (0.0467/0.0664)
	Displacement	11.60* / 17.24*** (0.1086/0.1035)	Displacement	13282.01*** / 61002.52*** (0.0420/0.1594)

Significance Levels (Benjamini-Hochberg): * $p < 0.05$; ** $p < 0.01$; *** $p < 0.001$

Table S13: Cluster separation by embedding space configuration (Configs 6-10). Reported per parameter set and domain are the Kruskal H scores and η^2 for the periods 1999/2023 (International Trade) and 2012/2024 (Public Procurement).

Config ID	Trade (1999/2023)		Procurement (2012/2024)	
	Feature	Kruskal H (η^2)	Feature	Kruskal H (η^2)
6	Alignment	34.78*** / 50.72*** (0.3222/0.3597)	Alignment	709.62*** / 67473.28*** (0.0025/0.1765)
	Portfolio Entropy	13.39/ 36.13*** (0.0549/0.2339)	Portfolio Entropy	18487.70*** / 144728.77*** (0.0679/0.3786)
	Capability Gap	40.47*** / 52.25*** (0.3934/0.3728)	Capability Gap	15106.71*** / 25231.07*** (0.0554/0.0659)
	Displacement	37.98*** / 38.77*** (0.3623/0.2567)	Displacement	6856.02*** / 55354.38*** (0.0251/0.1448)
7	Alignment	44.37*** / 71.48*** (0.3861/0.4839)	Alignment	902.98*** / 77780.06*** (0.0030/0.1705)
	Portfolio Entropy	25.01** / 73.05*** (0.1686/0.4964)	Portfolio Entropy	24973.72*** / 213610.25*** (0.0851/0.4684)
	Capability Gap	54.45*** / 67.31*** (0.4995/0.4505)	Capability Gap	11908.94*** / 30998.08*** (0.0405/0.0679)
	Displacement	49.42*** / 34.95*** (0.4430/0.1916)	Displacement	10758.65*** / 62010.70*** (0.0366/0.1359)
8	Alignment	27.76*** / 2.36ns (0.2776/0.0184)	Alignment	1200.04*** / 65807.93*** (0.0039/0.1409)
	Portfolio Entropy	19.37** / 0.28ns (0.1753/0.0000)	Portfolio Entropy	30654.80*** / 262312.59*** (0.1018/0.5619)
	Capability Gap	27.08*** / 1.62ns (0.2693/0.0084)	Capability Gap	15290.86*** / 21364.23*** (0.0507/0.0456)
	Displacement	22.87*** / 0.00ns (0.2179/0.0000)	Displacement	12525.27*** / 60598.46*** (0.0415/0.1297)
9	Alignment	38.13*** / 4.16ns (0.3509/0.0273)	Alignment	762.46*** / 66543.71*** (0.0028/0.1510)
	Portfolio Entropy	24.33** / 13.36** (0.1847/0.1438)	Portfolio Entropy	12704.49*** / 216707.84*** (0.0481/0.4920)
	Capability Gap	41.88*** / 1.61ns (0.3962/0.0000)	Capability Gap	9820.85*** / 21302.22*** (0.0372/0.0483)
	Displacement	32.56*** / 0.06ns (0.2839/0.0000)	Displacement	3366.49*** / 41941.12*** (0.0127/0.0952)
10	Alignment	30.17*** / 50.31*** (0.3266/0.3831)	Alignment	1453.83*** / 69928.00*** (0.0046/0.1740)
	Portfolio Entropy	8.62/ 43.26*** (0.0354/0.3126)	Portfolio Entropy	28254.14*** / 218511.10*** (0.0909/0.5439)
	Capability Gap	25.75*** / 47.80*** (0.2669/0.3580)	Capability Gap	14145.13*** / 22804.12*** (0.0454/0.0566)
	Displacement	21.33** / 35.74*** (0.2072/0.2374)	Displacement	12055.17*** / 66259.16*** (0.0387/0.1648)

Significance Levels (Benjamini-Hochberg): * $p < 0.05$; ** $p < 0.01$; *** $p < 0.001$

S7 Correlations

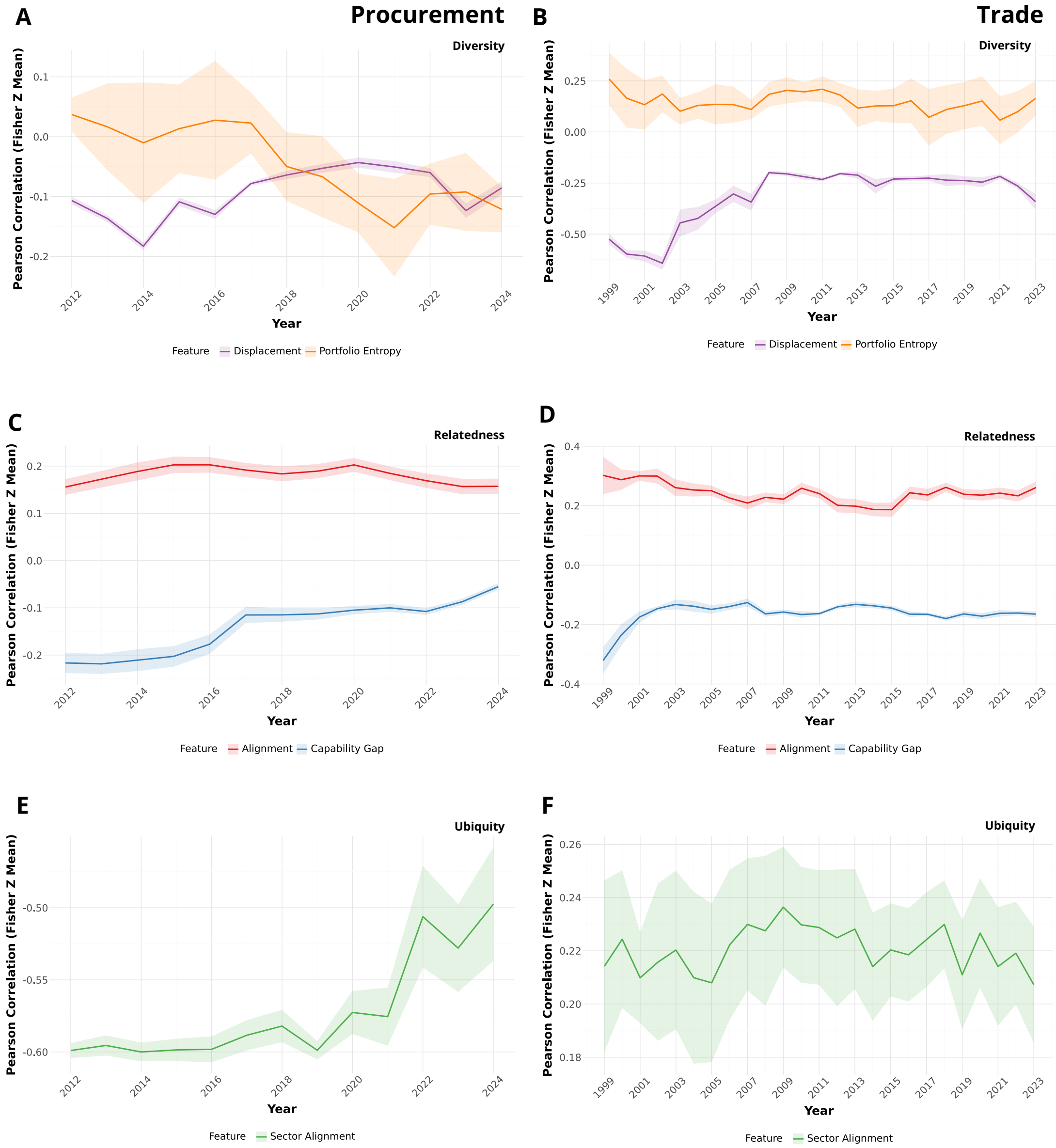

Figure S3: Correlations for embedding space features and relatedness, number of revealed specialization, and ubiquity over time. Panel A, C, and E show the public procurement domain, panels B, D, and F show the international trade domain. Correlations per embedding space configuration are transformed using the Fisher z-transform.

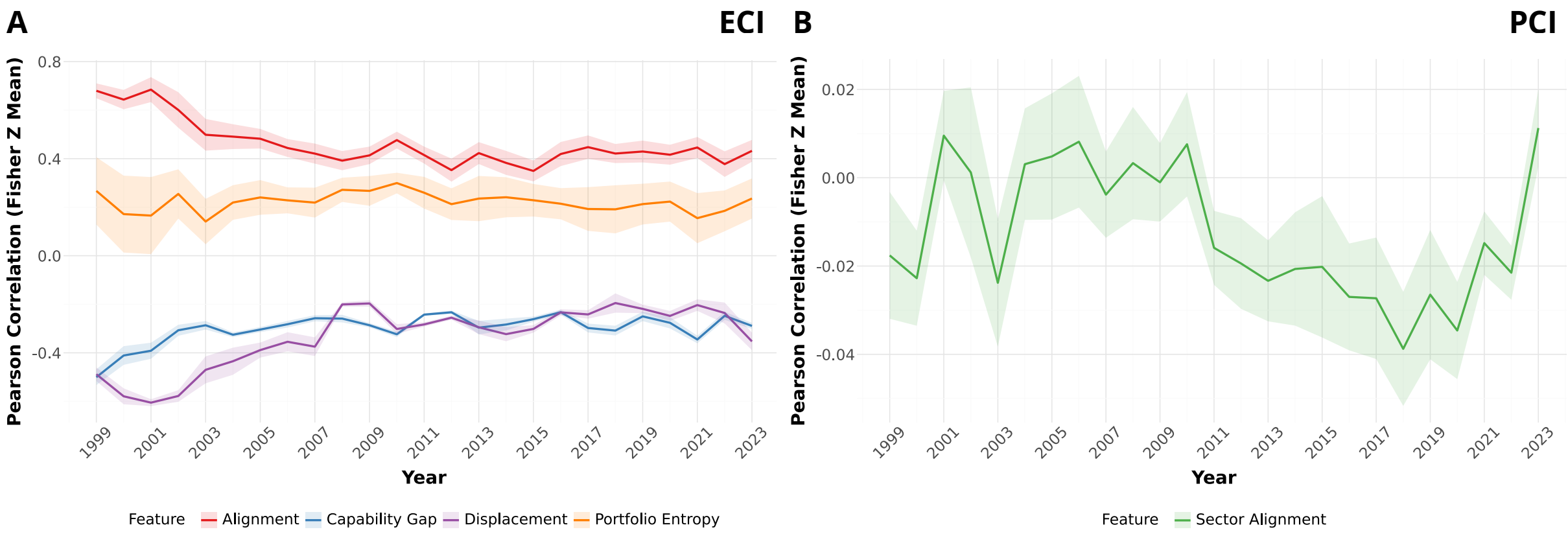

Figure S4: Correlations between complexity features (ECI and PCI) with embedding space features over time (international trade only). Correlations per embedding space configuration are transformed using the Fisher z-transform.

S8 Odds Ratio Analysis

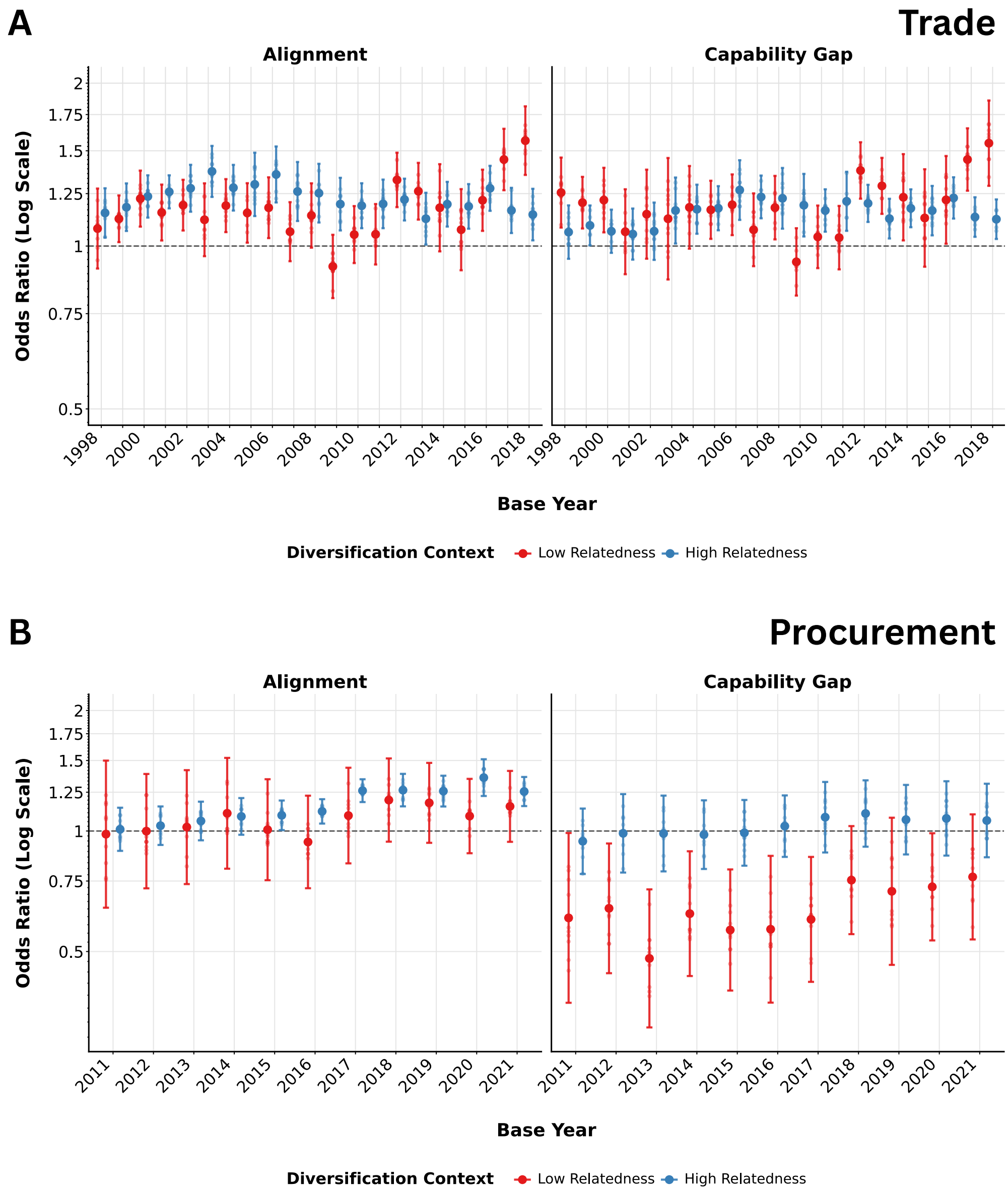

Figure S5: Lift from capability alignment/gap in low/high relatedness diversification. Odds ratios are defined based on the favorable condition (high alignment/low gap). Panel A presents the odds ratios for high alignment/low gap across both low- and high-relatedness (median-split) diversification cases in the international trade dataset, and Panel B presents the odds ratios for the public procurement dataset. The mean and error bars correspond to the pooled estimates over the embedding space configurations using Rubin's rules [6].

S9 SHAP Analysis

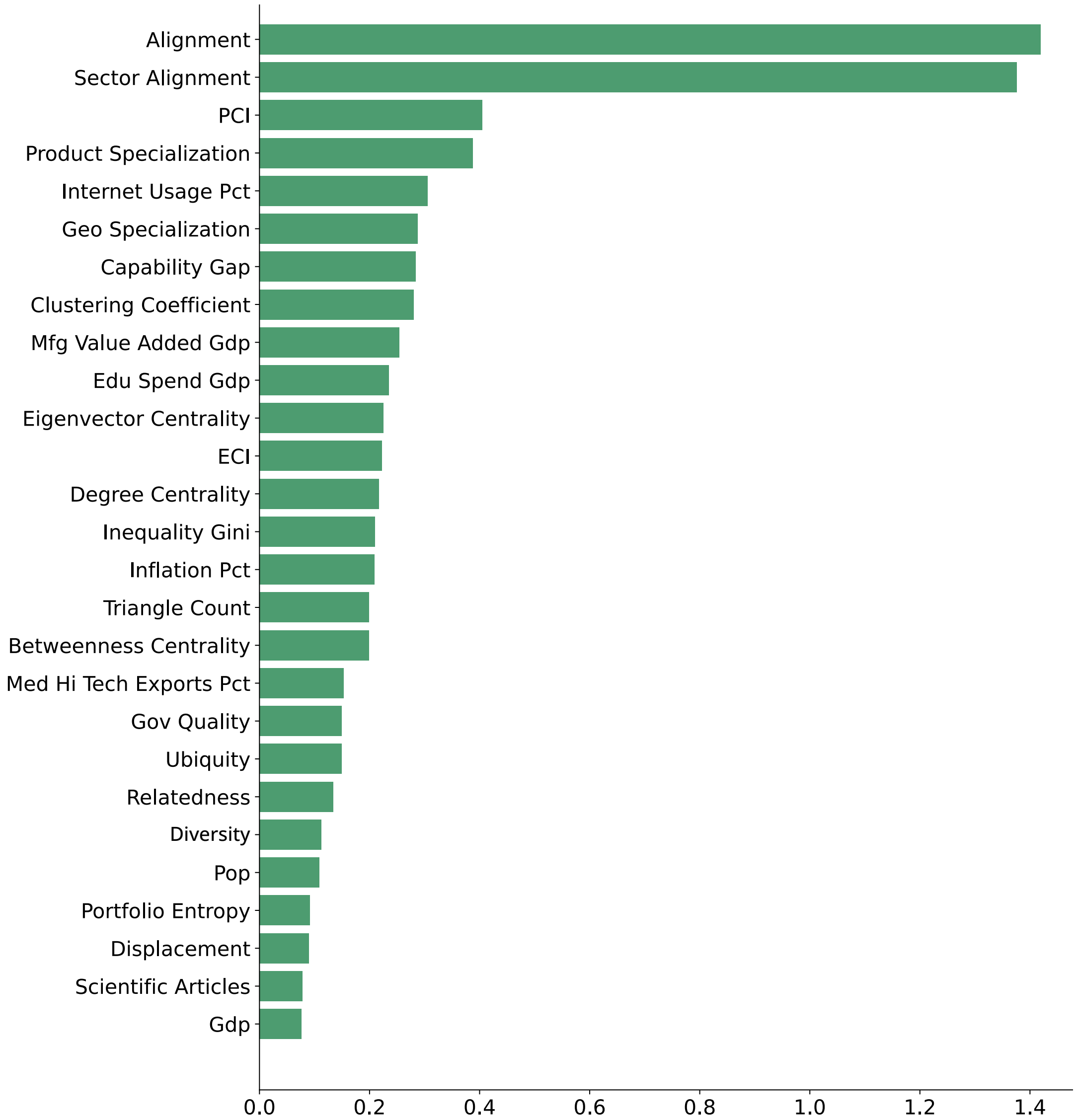

Figure S6: Mean absolute SHAP values for the international trade regression model (MLP).

S10 Data Dictionaries

Table S14: Feature dictionary for network and embedding-space measures. These features are computed identically for both datasets: for procurement, entities u are firms and activities a are CPV activity groups; for trade, entities are countries and activities are HS4 products. The proximity network G_t and target activity a are defined accordingly in each case.

**Feature**	**Description**	**Ref./Source**
Network measures (on the proximity network G_t, for target activity a)		
Degree centrality	Normalised count of a's direct links (non-zero $\phi_{a,a'}$) in G_t.	Standard
Clustering coefficient	Share of a's neighbours that are mutually connected.	Standard
Triangle count	Closed triplets containing a.	Standard
Betweenness centrality	Share of all-pairs shortest paths passing through a; bridging role.	Standard
Eigenvector centrality	Importance of a weighted by its neighbours' importance.	Standard
Embedding space		
Capability alignment	Cosine similarity, entity u vs. activity a.	Eq. 6
Capability gap	Euclidean distance, u vs. a.	Eq. 7
Displacement	Euclidean distance between u's Procrustes-aligned embeddings at t and $t-1$.	Eq. 12
Portfolio entropy	Shannon entropy of u's projection weights over K activity clusters.	Eqs. 9–11
Sectoral alignment	Mean cosine similarity of entities active in a to a's embedding; embedding analogue of ubiquity.	Eq. 8

Table S15: Feature dictionary for public procurement data. Network and embedding features are shared across datasets; see Table S14.

**Feature**	**Description**	**Ref./Source**
Firm level (HHI = Herfindahl–Hirschman index of contract count share)		
Geographic HHI	HHI across regions.	Portal BASE
Public entity HII	HHI across contracting authorities.	Portal BASE
Activity HHI	HHI across CPV activity groups.	Portal BASE
N° contracts won	Contracts awarded in the window.	Portal BASE
Total awards (log10)	Total awarded value, log-transformed.	Portal BASE
Tender winning rate	Contracts won / tenders bid.	Portal BASE
Mean contract value per CPV (log10)	Mean awarded value within a CPV group, log-transformed.	Portal BASE
Economic complexity		
Relatedness	Weighted mean proximity of a to u's portfolio.	Eq. 4
Diversity	Activities with RS ≥ 1 (k_u).	Eq. 2
Ubiquity	Entities with RS ≥ 1 in a (k_a).	Eq. 2

Table S16: Feature dictionary for international trade data. Network and embedding features are shared across datasets; see Table S14, with countries as entities and HS4 products as activities.

**Feature**	**Description**	**Ref./Source**
Country level		
Product HHI	HHI of export value across HS4 products.	OEC
Trading-partner HHI	HHI of trade value across partner countries.	OEC
Inequality (Gini)	Gini coefficient of income distribution.	`SI.POV.GINI`
Inflation (%)	Annual change in consumer prices.	`FP.CPI.TOTL.ZG`
GDP, current US$ (log10)	GDP at current prices, log-transformed.	`NY.GDP.MKTP.CD`
Population (log10)	Total population, log-transformed.	`SP.POP.TOTL`
Education spending (% GDP)	Government expenditure on education.	`SE.XPD.TOTL.GD.ZS`
Government quality	Government Effectiveness: perceived quality of public services, civil service and policy formulation, and credibility of government commitment.	`GE.EST`, WGI
Medium/high-tech exports (%)	Share of manufactured exports that are medium- or high-tech.	`TX.MNF.TECH.ZS.UN`
Scientific articles	Scientific and technical journal articles.	`IP.JRN.ARTC.SC`
Internet usage (%)	Share of population using the internet.	`IT.NET.USER.ZS`
Manufacturing value added (% GDP)	Value added by manufacturing.	`NV.IND.MANF.ZS`
Economic complexity		
Relatedness	Weighted mean proximity of a to the country's portfolio.	Eq. 4
Diversity	Products with RS ≥ 1 (k_u).	Eq. 2
Ubiquity	Countries with RS ≥ 1 in a (k_a).	Eq. 2
ECI	Economic Complexity Index of the export basket.	[3]
PCI	Product Complexity Index.	[3]

S11 Ranking evaluation of ML models (Main Set)

Table S17: Entry ranking performance by domain, task and model, at global and per-entity scope. *Global* rankings pool all candidates into one ranking and are scored at $k \in \{100, 500, 1000\}$. *per-entity* ranks within each entity and is macro-averaged over the entities, scored at $k \in \{5, 10, 20, 50\}$. For the regression models, each entity's ranked recommendation is derived by ordering its currently inactive entries, i.e. activities with RS < 1, by the magnitude of the predicted RS, so that a larger predicted RS denotes a stronger recommendation. Best precision and recall within each domain × scope × k group (across all models, baselines included) are in **bold**.

	Global (pooled)			Per-entity (macro-avg)		
Model	k	P@k	R@k	k	P@k	R@k
**Trade**						
Baseline						
Persistence	100	**0.4600**	**0.0012**	5	**0.3153**	**0.0526**
	500	**0.4040**	**0.0055**	10	**0.3023**	**0.0985**
	1000	**0.3780**	**0.0102**	20	**0.2829**	**0.1736**
				50	**0.2305**	**0.3243**
Random	100	0.0500	0.0001	5	0.0372	0.0045
	500	0.0380	0.0005	10	0.0366	0.0088
	1000	0.0390	0.0011	20	0.0378	0.0179
				50	0.0389	0.0472
Classification						
XGBoost	100	0.4100	0.0011	5	0.1687	0.0297
	500	0.3080	0.0042	10	0.1474	0.0490
	1000	0.2690	0.0073	20	0.1270	0.0805
				50	0.1074	0.1601
Random Forest	100	0.4000	0.0011	5	0.1750	0.0302
	500	0.3140	0.0042	10	0.1579	0.0518
	1000	0.2730	0.0074	20	0.1360	0.0867
				50	0.1110	0.1648
MLP	100	0.3800	0.0010	5	0.1389	0.0249
	500	0.2160	0.0029	10	0.1241	0.0403
	1000	0.1570	0.0042	20	0.1116	0.0679
				50	0.0973	0.1407
Decision Tree	100	0.1200	0.0003	5	0.0939	0.0152
	500	0.1220	0.0017	10	0.0904	0.0294
	1000	0.0960	0.0026	20	0.0827	0.0503
				50	0.0751	0.1037
Logistic Regression	100	0.1800	0.0005	5	0.1155	0.0214
	500	0.1180	0.0016	10	0.1085	0.0399
	1000	0.1160	0.0031	20	0.0992	0.0664
				50	0.0828	0.1303
Regression						
XGBoost	100	0.0700	0.0002	5	0.0891	0.0148

Table S17 – continued

Model	Global (pooled)			Per-entity (macro-avg)		
	k	P@k	R@k	k	P@k	R@k
	500	0.0600	0.0008	10	0.0860	0.0276
	1000	0.0680	0.0018	20	0.0851	0.0540
				50	0.0803	0.1194
Linear Regression	100	0.0000	0.0000	5	0.0374	0.0057
	500	0.0160	0.0002	10	0.0415	0.0134
	1000	0.0220	0.0006	20	0.0418	0.0265
				50	0.0423	0.0616
Ridge	100	0.0000	0.0000	5	0.0376	0.0057
	500	0.0160	0.0002	10	0.0416	0.0134
	1000	0.0220	0.0006	20	0.0418	0.0265
				50	0.0423	0.0616
Lasso	100	0.0000	0.0000	5	0.0384	0.0059
	500	0.0140	0.0002	10	0.0411	0.0125
	1000	0.0220	0.0006	20	0.0419	0.0261
				50	0.0432	0.0622
MLP	100	0.0600	0.0002	5	0.0414	0.0055
	500	0.0500	0.0007	10	0.0378	0.0097
	1000	0.0430	0.0012	20	0.0361	0.0176
				50	0.0368	0.0449
Decision Tree	100	0.0000	0.0000	5	0.0508	0.0085
	500	0.0160	0.0002	10	0.0522	0.0154
	1000	0.0260	0.0007	20	0.0567	0.0311
				50	0.0617	0.0828
Random Forest	100	0.0300	0.0001	5	0.0479	0.0064
	500	0.0340	0.0005	10	0.0555	0.0166
	1000	0.0340	0.0009	20	0.0629	0.0352
				50	0.0683	0.0938
**Procurement**						
Baseline						
Persistence	100	0.3900	0.0018	5	0.0369	0.0390
	500	0.3740	0.0084	10	0.0256	0.0528
	1000	0.3410	0.0154	20	0.0164	0.0722
				50	0.0117	0.1142
Random	100	0.0100	0.0000	5	0.0205	0.0185
	500	0.0180	0.0004	10	0.0202	0.0360
	1000	0.0200	0.0009	20	0.0205	0.0705
				50	0.0204	0.1796
Classification						
XGBoost	100	0.6400	0.0029	5	0.2725	0.3046
	500	0.5700	0.0129	10	**0.2132**	0.4468
	1000	0.5290	0.0239	20	**0.1552**	**0.6169**
				50	**0.0885**	**0.8220**

Table S17 – continued

Model	Global (pooled)			Per-entity (macro-avg)		
	k	P@k	R@k	k	P@k	R@k
Random Forest	100	**0.6900**	**0.0031**	5	0.2702	0.3004
	500	**0.5860**	**0.0132**	10	0.2128	0.4479
	1000	**0.5450**	**0.0246**	20	0.1537	0.6088
				50	0.0880	0.8185
MLP	100	0.5100	0.0023	5	**0.2727**	**0.3066**
	500	0.5160	0.0116	10	**0.2132**	**0.4511**
	1000	0.5090	0.0230	20	0.1540	0.6105
				50	0.0881	0.8203
Decision Tree	100	0.5000	0.0023	5	0.2291	0.2592
	500	0.4980	0.0112	10	0.1830	0.3897
	1000	0.4850	0.0219	20	0.1397	0.5614
				50	0.0841	0.7855
Logistic Regression	100	0.0100	0.0000	5	0.2471	0.2827
	500	0.1820	0.0041	10	0.1979	0.4212
	1000	0.2440	0.0110	20	0.1467	0.5836
				50	0.0863	0.8029
Regression						
XGBoost	100	0.1300	0.0006	5	0.1358	0.1667
	500	0.1240	0.0028	10	0.1223	0.2748
	1000	0.1330	0.0060	20	0.1061	0.4390
				50	0.0755	0.7194
Linear Regression	100	0.0500	0.0002	5	0.1062	0.1153
	500	0.1400	0.0032	10	0.0930	0.1872
	1000	0.1540	0.0069	20	0.0762	0.2884
				50	0.0537	0.4756
Ridge	100	0.0500	0.0002	5	0.1062	0.1153
	500	0.1400	0.0032	10	0.0930	0.1872
	1000	0.1540	0.0069	20	0.0762	0.2884
				50	0.0537	0.4756
Lasso	100	0.0500	0.0002	5	0.1061	0.1155
	500	0.1400	0.0032	10	0.0929	0.1872
	1000	0.1540	0.0069	20	0.0761	0.2882
				50	0.0536	0.4747
MLP	100	0.0900	0.0004	5	0.0907	0.1010
	500	0.0780	0.0018	10	0.0829	0.1740
	1000	0.0680	0.0031	20	0.0723	0.2881
				50	0.0528	0.4979
Decision Tree	100	0.0000	0.0000	5	0.0509	0.0629
	500	0.0040	0.0001	10	0.0517	0.1145
	1000	0.0130	0.0006	20	0.0459	0.1793
				50	0.0308	0.2811
	100	0.0300	0.0001	5	0.0458	0.0555
Random Forest						

Table S17 – continued

	Global (pooled)			Per-entity (macro-avg)		
Model	k	P@k	R@k	k	P@k	R@k
	500	0.0220	0.0005	10	0.0493	0.1117
	1000	0.0230	0.0010	20	0.0504	0.2157
				50	0.0472	0.4578

S12 Ablation Summaries

S12.1 Classification/Regression

S12.1.1 No EC features

Table S18: Comparison of baseline and advanced models for regression and classification tasks across Trade and Procurement domains, for the ablation excluding the economic complexity features (including relatedness) from the full feature set. Models are ordered from simpler to more complex. Baseline models are marked in italics. The best model for RMSE and F1 is highlighted, and the best for Precision/Recall/ROC-AUC and Average Precision (AP) is underlined. For ROC-AUC and AP the random baselines are the by-construction rates, i.e. 0.5 for ROC-AUC and prevalence for AP.

Domain	Test RMSE	Test F1	Test Precision	Test Recall	Test ROC-AUC	Test AP	Model
**International Trade**							
	**20.7366**	**0.2790**	<u>0.2006</u>	0.4580	<u>0.7870</u>	<u>0.1924</u>	*Persistence (Baseline)*
	39.2799	0.0418	0.0385	0.0457	0.5000	0.0374	*Random (Baseline)*
	24.9650	0.0786	0.0423	0.5547	0.5385	0.0398	Linear Regression
	24.9650	0.0786	0.0423	0.5547	0.5385	0.0398	Ridge
**Regression**	24.9809	0.0721	0.0374	<u>1.0000</u>	0.5000	0.0374	Lasso
	27.4583	0.0895	0.0485	0.5771	0.5892	0.0444	Decision Tree
	22.3057	0.0831	0.0438	0.8190	0.5597	0.0412	Random Forest
	22.9236	0.1061	0.0598	0.4669	0.6438	0.0550	XGBoost
	23.6290	0.0860	0.0460	0.6487	0.5804	0.0460	MLP
		**0.2790**	<u>0.2006</u>	0.4580	<u>0.7870</u>	<u>0.1924</u>	*Persistence (Baseline)*
		0.0418	0.0385	0.0457	0.5000	0.0374	*Random (Baseline)*
		0.1077	0.0629	0.3738	0.6381	0.0586	Logistic Regression
**Classification**		0.0722	0.0762	0.0685	0.6690	0.0635	Decision Tree
		0.1200	0.0796	0.2435	0.6700	0.0667	Random Forest
		0.0873	0.0842	0.0907	0.6772	0.0664	XGBoost
		0.1021	0.0561	0.5686	0.6404	0.0571	MLP
**Procurement**							
	37.2930	0.0481	<u>0.1974</u>	0.0274	0.5125	0.0275	*Persistence (Baseline)*
	33.5045	0.0182	0.0177	0.0188	0.5000	0.0187	*Random (Baseline)*
	25.7546	0.0407	0.0210	0.6483	0.5550	0.0226	Linear Regression
	25.7546	0.0407	0.0210	0.6483	0.5550	0.0226	Ridge
**Regression**	25.7544	0.0409	0.0211	0.6500	0.5570	0.0228	Lasso
	26.6564	0.0453	0.0235	0.6413	0.5870	0.0242	Decision Tree
	26.1871	0.0439	0.0226	<u>0.8076</u>	0.5954	0.0233	Random Forest
	**24.8917**	0.0524	0.0278	0.4521	0.6108	0.0272	XGBoost
	26.5311	0.0417	0.0215	0.6665	0.5598	0.0215	MLP
		0.0481	<u>0.1974</u>	0.0274	0.5125	0.0275	*Persistence (Baseline)*
		0.0182	0.0177	0.0188	0.5000	0.0187	*Random (Baseline)*
		0.1191	0.0789	0.2426	0.7662	0.0595	Logistic Regression
**Classification**		0.1238	0.1319	0.1166	0.7898	0.0768	Decision Tree
		0.1389	0.0972	0.2432	0.7852	0.0757	Random Forest
		**0.1431**	0.0981	0.2646	<u>0.7936</u>	<u>0.0802</u>	XGBoost
		0.1131	0.0711	0.2765	0.7601	0.0616	MLP

S12.1.2 No Embedding features

Table S19: Comparison of baseline and advanced models for regression and classification tasks across Trade and Procurement domains, for the ablation excluding the embedding-derived features from the full feature set. Models are ordered from simpler to more complex. Baseline models are marked in italics. The best model for RMSE and F1 is highlighted, and the best for Precision/Recall/ROC-AUC and Average Precision (AP) is underlined. For ROC-AUC and AP the random baselines are the by-construction rates, i.e. 0.5 for ROC-AUC and prevalence for AP.

Domain	Test RMSE	Test F1	Test Precision	Test Recall	Test ROC-AUC	Test AP	Model
**International Trade**							
	**20.7366**	**0.2790**	<u>0.2006</u>	0.4580	<u>0.7870</u>	<u>0.1924</u>	*Persistence (Baseline)*
	39.2799	0.0418	0.0385	0.0457	0.5000	0.0374	*Random (Baseline)*
	24.9428	0.0834	0.0448	0.6119	0.5750	0.0462	Linear Regression
	24.9428	0.0834	0.0448	0.6119	0.5748	0.0461	Ridge
**Regression**	24.9427	0.0833	0.0447	0.6117	0.5737	0.0460	Lasso
	25.1119	0.1131	0.0633	0.5261	0.6724	0.0594	Decision Tree
	24.5098	0.1045	0.0563	0.7206	0.6588	0.0564	Random Forest
	24.7759	0.1252	0.0715	0.5054	0.6963	0.0673	XGBoost
	24.7484	0.0995	0.0533	<u>0.7384</u>	0.6534	0.0599	MLP
		**0.2790**	<u>0.2006</u>	0.4580	<u>0.7870</u>	<u>0.1924</u>	*Persistence (Baseline)*
		0.0418	0.0385	0.0457	0.5000	0.0374	*Random (Baseline)*
		0.1309	0.0837	0.3000	0.6924	0.0724	Logistic Regression
**Classification**		0.1238	0.1320	0.1166	0.7324	0.0923	Decision Tree
		0.1446	0.1095	0.2130	0.7287	0.0885	Random Forest
		0.1462	0.1184	0.1908	0.7350	0.0930	XGBoost
		0.1424	0.0850	0.4368	0.7149	0.0827	MLP
**Procurement**							
	37.2930	0.0481	0.1974	0.0274	0.5125	0.0275	*Persistence (Baseline)*
	33.5045	0.0182	0.0177	0.0188	0.5000	0.0187	*Random (Baseline)*
	25.8170	0.0673	0.0351	0.8437	0.8065	0.1124	Linear Regression
	25.8170	0.0673	0.0351	0.8437	0.8065	0.1124	Ridge
**Regression**	25.8187	0.0701	0.0366	0.8285	0.8022	0.1108	Lasso
	25.9311	0.1069	0.0580	0.6744	0.8146	0.0675	Decision Tree
	25.3192	0.0952	0.0504	<u>0.8481</u>	0.8471	0.0945	Random Forest
	**25.1942**	0.1493	0.0832	0.7219	0.8751	0.1170	XGBoost
	25.2808	0.1239	0.0687	0.6306	0.8147	0.0868	MLP
		0.0481	0.1974	0.0274	0.5125	0.0275	*Persistence (Baseline)*
		0.0182	0.0177	0.0188	0.5000	0.0187	*Random (Baseline)*
		0.2495	0.2135	0.3002	0.8960	0.1640	Logistic Regression
**Classification**		0.2663	<u>0.2614</u>	0.2714	0.9039	0.1988	Decision Tree
		0.2593	0.1865	0.4256	0.9011	0.1847	Random Forest
		**0.2802**	0.2332	0.3509	<u>0.9068</u>	<u>0.2063</u>	XGBoost
		0.2766	0.2414	0.3239	0.9035	0.2003	MLP

S12.1.3 No Relatedness

Table S20: Comparison of baseline and advanced models for regression and classification tasks across Trade and Procurement domains, for the ablation excluding the relatedness feature from the full feature set. Models are ordered from simpler to more complex. Baseline models are marked in italics. The best model for RMSE and F1 is highlighted, and the best for Precision/Recall/ROC-AUC and Average Precision (AP) is underlined. For ROC-AUC and AP the random baselines are the by-construction rates, i.e. 0.5 for ROC-AUC and prevalence for AP.

Domain	Test RMSE	Test F1	Test Precision	Test Recall	Test ROC-AUC	Test AP	Model
**International Trade**							
	**20.7366**	**0.2790**	<u>0.2006</u>	0.4580	<u>0.7870</u>	<u>0.1924</u>	*Persistence (Baseline)*
	39.2799	0.0418	0.0385	0.0457	0.5000	0.0374	*Random (Baseline)*
	24.9438	0.0787	0.0423	0.5659	0.5482	0.0423	Linear Regression
	24.9438	0.0787	0.0423	0.5660	0.5482	0.0423	Ridge
**Regression**	24.9192	0.0794	0.0425	0.6108	0.5535	0.0433	Lasso
	24.2208	0.1033	0.0565	0.5939	0.6353	0.0535	Decision Tree
	21.9527	0.1078	0.0585	<u>0.6826</u>	0.6641	0.0579	Random Forest
	22.4470	0.1241	0.0705	0.5170	0.6952	0.0667	XGBoost
	22.3054	0.0958	0.0605	0.2304	0.6242	0.0544	MLP
		**0.2790**	<u>0.2006</u>	0.4580	<u>0.7870</u>	<u>0.1924</u>	*Persistence (Baseline)*
		0.0418	0.0385	0.0457	0.5000	0.0374	*Random (Baseline)*
		0.1275	0.0828	0.2773	0.6866	0.0707	Logistic Regression
**Classification**		0.1206	0.1297	0.1128	0.7306	0.0914	Decision Tree
		0.1415	0.1122	0.1918	0.7289	0.0885	Random Forest
		0.1281	0.1302	0.1260	0.7343	0.0931	XGBoost
		0.1350	0.1091	0.1771	0.7157	0.0826	MLP
**Procurement**							
	37.2930	0.0481	<u>0.1974</u>	0.0274	0.5125	0.0275	*Persistence (Baseline)*
	33.5045	0.0182	0.0177	0.0188	0.5000	0.0187	*Random (Baseline)*
	25.7531	0.0411	0.0212	0.6536	0.5581	0.0227	Linear Regression
	25.7531	0.0411	0.0212	0.6536	0.5581	0.0227	Ridge
**Regression**	25.7531	0.0411	0.0212	0.6547	0.5584	0.0228	Lasso
	25.3925	0.0545	0.0287	0.5306	0.6371	0.0250	Decision Tree
	27.8744	0.0456	0.0236	<u>0.6673</u>	0.5736	0.0214	Random Forest
	24.8949	0.0537	0.0286	0.4455	0.6197	0.0280	XGBoost
	**24.4752**	0.0623	0.0336	0.4296	0.6391	0.0295	MLP
		0.0481	<u>0.1974</u>	0.0274	0.5125	0.0275	*Persistence (Baseline)*
		0.0182	0.0177	0.0188	0.5000	0.0187	*Random (Baseline)*
		0.1366	0.0914	0.2699	0.7848	0.0717	Logistic Regression
**Classification**		0.1373	0.1564	0.1223	0.8054	0.0890	Decision Tree
		0.1463	0.1333	0.1620	0.7995	0.0853	Random Forest
		**0.1587**	0.1164	0.2491	<u>0.8076</u>	<u>0.0904</u>	XGBoost
		0.1346	0.0895	0.2712	0.7895	0.0691	MLP

S12.1.4 EC-only

Table S21: Comparison of baseline and advanced models for regression and classification tasks across Trade and Procurement domains, for the ablation using only the economic complexity features (including relatedness). Models are ordered from simpler to more complex. Baseline models are marked in italics. The best model for RMSE and F1 is highlighted, and the best for Precision/Recall/ROC-AUC and Average Precision (AP) is underlined. For ROC-AUC and AP the random baselines are the by-construction rates, i.e. 0.5 for ROC-AUC and prevalence for AP.

Domain	Test RMSE	Test F1	Test Precision	Test Recall	Test ROC-AUC	Test AP	Model
**International Trade**							
	**20.7366**	**0.2790**	<u>0.2006</u>	0.4580	<u>0.7870</u>	<u>0.1924</u>	*Persistence (Baseline)*
	39.2799	0.0418	0.0385	0.0457	0.5000	0.0374	*Random (Baseline)*
	24.9532	0.0815	0.0438	0.5868	0.5571	0.0443	Linear Regression
	24.9532	0.0815	0.0438	0.5868	0.5571	0.0443	Ridge
**Regression**	24.9532	0.0814	0.0437	0.5881	0.5571	0.0443	Lasso
	24.9207	0.1082	0.0608	0.4907	0.6515	0.0552	Decision Tree
	24.7503	0.1035	0.0558	<u>0.7047</u>	0.6521	0.0552	Random Forest
	24.8514	0.1179	0.0658	0.5706	0.6845	0.0643	XGBoost
	24.8453	0.0969	0.0537	0.4953	0.6176	0.0527	MLP
		**0.2790**	<u>0.2006</u>	0.4580	<u>0.7870</u>	<u>0.1924</u>	*Persistence (Baseline)*
		0.0418	0.0385	0.0457	0.5000	0.0374	*Random (Baseline)*
		0.1286	0.0785	0.3555	0.6898	0.0702	Logistic Regression
**Classification**		0.1322	0.1272	0.1377	0.7260	0.0908	Decision Tree
		0.1462	0.1081	0.2258	0.7251	0.0872	Random Forest
		0.1260	0.1269	0.1251	0.7275	0.0912	XGBoost
		0.1366	0.1074	0.1877	0.7180	0.0852	MLP
**Procurement**							
	37.2930	0.0481	0.1974	0.0274	0.5125	0.0275	*Persistence (Baseline)*
	33.5045	0.0182	0.0177	0.0188	0.5000	0.0187	*Random (Baseline)*
	25.8306	0.0758	0.0398	<u>0.7964</u>	0.7897	0.1084	Linear Regression
	25.8306	0.0758	0.0398	<u>0.7964</u>	0.7897	0.1084	Ridge
**Regression**	25.8306	0.0758	0.0398	<u>0.7964</u>	0.7897	0.1084	Lasso
	25.6202	0.1262	0.0699	0.6452	0.8357	0.0796	Decision Tree
	**25.5406**	0.1299	0.0719	0.6720	0.8449	0.0869	Random Forest
	25.6097	0.1439	0.0804	0.6876	0.8619	0.0985	XGBoost
	25.5620	0.1254	0.0692	0.6665	0.8407	0.0888	MLP
		0.0481	0.1974	0.0274	0.5125	0.0275	*Persistence (Baseline)*
		0.0182	0.0177	0.0188	0.5000	0.0187	*Random (Baseline)*
		0.2424	0.1828	0.3593	0.8892	0.1529	Logistic Regression
**Classification**		0.2583	<u>0.2366</u>	0.2843	0.9000	<u>0.1884</u>	Decision Tree
		0.2557	0.2365	0.2782	0.8995	0.1848	Random Forest
		**0.2621**	0.2177	0.3294	<u>0.9001</u>	0.1865	XGBoost
		0.2580	0.2258	0.3011	0.8997	0.1882	MLP

S12.1.5 Embedding-derived features only

Table S22: Comparison of baseline and advanced models for regression and classification tasks across Trade and Procurement domains, for the ablation using only the embedding-derived features. Models are ordered from simpler to more complex. Baseline models are marked in italics. The best model for RMSE and F1 is highlighted, and the best for Precision/Recall/ROC-AUC and Average Precision (AP) is underlined. For ROC-AUC and AP the random baselines are the by-construction rates, i.e. 0.5 for ROC-AUC and prevalence for AP.

Domain	Test RMSE	Test F1	Test Precision	Test Recall	Test ROC-AUC	Test AP	Model
**International Trade**							
	**20.7366**	**0.2790**	<u>0.2006</u>	0.4580	<u>0.7870</u>	<u>0.1924</u>	*Persistence (Baseline)*
	39.2799	0.0418	0.0385	0.0457	0.5000	0.0374	*Random (Baseline)*
	24.9781	0.0757	0.0405	0.5761	0.5250	0.0382	Linear Regression
	24.9781	0.0757	0.0405	0.5761	0.5250	0.0382	Ridge
**Regression**	24.9809	0.0721	0.0374	<u>1.0000</u>	0.5000	0.0374	Lasso
	24.5603	0.0728	0.0382	0.7546	0.4882	0.0354	Decision Tree
	22.9234	0.0755	0.0396	0.8245	0.4976	0.0349	Random Forest
	23.3326	0.0865	0.0471	0.5315	0.5774	0.0444	XGBoost
	25.7466	0.0710	0.0411	0.2598	0.5390	0.0401	MLP
		**0.2790**	<u>0.2006</u>	0.4580	<u>0.7870</u>	<u>0.1924</u>	*Persistence (Baseline)*
		0.0418	0.0385	0.0457	0.5000	0.0374	*Random (Baseline)*
		0.0987	0.0561	0.4102	0.6034	0.0514	Logistic Regression
**Classification**		0.0662	0.0887	0.0528	0.6469	0.0626	Decision Tree
		0.1020	0.0803	0.1397	0.6480	0.0626	Random Forest
		0.1109	0.0816	0.1731	0.6506	0.0636	XGBoost
		0.1011	0.0561	0.5073	0.6255	0.0581	MLP
**Procurement**							
	37.2930	0.0481	<u>0.1974</u>	0.0274	0.5125	0.0275	*Persistence (Baseline)*
	33.5045	0.0182	0.0177	0.0188	0.5000	0.0187	*Random (Baseline)*
	25.7633	0.0400	0.0206	0.6688	0.5539	0.0227	Linear Regression
	25.7633	0.0400	0.0206	0.6688	0.5539	0.0227	Ridge
**Regression**	25.7626	0.0402	0.0207	0.6594	0.5549	0.0228	Lasso
	25.7632	0.0386	0.0197	<u>0.8767</u>	0.5436	0.0217	Decision Tree
	26.5364	0.0399	0.0206	0.6795	0.5482	0.0212	Random Forest
	**25.1148**	0.0456	0.0245	0.3317	0.5607	0.0237	XGBoost
	26.5009	0.0386	0.0199	0.6364	0.5318	0.0209	MLP
		0.0481	<u>0.1974</u>	0.0274	0.5125	0.0275	*Persistence (Baseline)*
		0.0182	0.0177	0.0188	0.5000	0.0187	*Random (Baseline)*
		0.0803	0.0590	0.1260	0.7117	<u>0.0436</u>	Logistic Regression
**Classification**		0.0741	0.0549	0.1142	0.7090	0.0424	Decision Tree
		0.0277	0.0604	0.0180	0.7053	0.0390	Random Forest
		0.0784	0.0510	0.1701	0.7104	0.0433	XGBoost
		**0.0817**	0.0518	0.1929	<u>0.7178</u>	0.0413	MLP

S12.1.6 Relatedness-only

Table S23: Comparison of baseline and advanced models for regression and classification tasks across Trade and Procurement domains, for the ablation using only the relatedness feature. Models are ordered from simpler to more complex. Baseline models are marked in italics. The best model for RMSE and F1 is highlighted, and the best for Precision/Recall/ROC-AUC and Average Precision (AP) is underlined. For ROC-AUC and AP the random baselines are the by-construction rates, i.e. 0.5 for ROC-AUC and prevalence for AP.

Domain	Test RMSE	Test F1	Test Precision	Test Recall	Test ROC-AUC	Test AP	Model
**International Trade**							
	**20.7366**	**0.2790**	<u>0.2006</u>	0.4580	<u>0.7870</u>	<u>0.1924</u>	*Persistence (Baseline)*
	39.2799	0.0418	0.0385	0.0457	0.5000	0.0374	*Random (Baseline)*
	24.9764	0.0717	0.0380	0.6454	0.5228	0.0418	Linear Regression
	24.9764	0.0717	0.0380	0.6454	0.5228	0.0418	Ridge
**Regression**	24.9765	0.0713	0.0377	0.6585	0.5189	0.0408	Lasso
	24.9782	0.0774	0.0403	<u>0.9804</u>	0.4905	0.0372	Decision Tree
	24.9684	0.0809	0.0426	0.8054	0.5358	0.0382	Random Forest
	24.9692	0.0870	0.0462	0.7484	0.5754	0.0427	XGBoost
	24.9746	0.0769	0.0408	0.6621	0.5200	0.0374	MLP
		**0.2790**	<u>0.2006</u>	0.4580	<u>0.7870</u>	<u>0.1924</u>	*Persistence (Baseline)*
		0.0418	0.0385	0.0457	0.5000	0.0374	*Random (Baseline)*
		0.1158	0.0720	0.2963	0.6541	0.0626	Logistic Regression
**Classification**		0.0802	0.0859	0.0753	0.6521	0.0631	Decision Tree
		0.1163	0.0813	0.2039	0.6674	0.0661	Random Forest
		0.1049	0.0862	0.1338	0.6664	0.0659	XGBoost
		0.0986	0.0530	0.7029	0.6445	0.0575	MLP
**Procurement**							
	37.2930	0.0481	<u>0.1974</u>	0.0274	0.5125	0.0275	*Persistence (Baseline)*
	33.5045	0.0182	0.0177	0.0188	0.5000	0.0187	*Random (Baseline)*
	25.8978	0.0878	0.0461	<u>0.9007</u>	<u>0.8681</u>	<u>0.1288</u>	Linear Regression
	25.8978	0.0878	0.0461	<u>0.9007</u>	<u>0.8681</u>	<u>0.1288</u>	Ridge
**Regression**	25.8978	0.0878	0.0461	<u>0.9007</u>	<u>0.8681</u>	<u>0.1288</u>	Lasso
	25.8962	0.0931	0.0491	0.8834	0.8431	0.1086	Decision Tree
	25.9093	0.1109	0.0595	0.8191	0.8651	0.1184	Random Forest
	25.9010	0.1093	0.0585	0.8289	0.8667	0.1274	XGBoost
	**25.8862**	0.1169	0.0630	0.8072	<u>0.8681</u>	<u>0.1288</u>	MLP
		0.0481	<u>0.1974</u>	0.0274	0.5125	0.0275	*Persistence (Baseline)*
		0.0182	0.0177	0.0188	0.5000	0.0187	*Random (Baseline)*
		0.2116	0.1480	0.3711	<u>0.8681</u>	<u>0.1288</u>	Logistic Regression
**Classification**		0.2123	0.1530	0.3464	0.8672	0.1165	Decision Tree
		0.2123	0.1518	0.3532	0.8667	0.1198	Random Forest
		0.2127	0.1583	0.3240	0.8680	0.1279	XGBoost
		**0.2128**	0.1582	0.3249	<u>0.8681</u>	<u>0.1288</u>	MLP

S12.1.7 Baseline Features

Table S24: Comparison of baseline and advanced models for regression and classification tasks across Trade and Procurement domains, for the ablation using only the baseline features, i.e. excluding both the economic complexity and the embedding-derived features. Models are ordered from simpler to more complex. Baseline models are marked in italics. The best model for RMSE and F1 is highlighted, and the best for Precision/Recall/ROC-AUC and Average Precision (AP) is underlined. For ROC-AUC and AP the random baselines are the by-construction rates, i.e. 0.5 for ROC-AUC and prevalence for AP.

Domain	Test RMSE	Test F1	Test Precision	Test Recall	Test ROC-AUC	Test AP	Model
**International Trade**							
	**20.7366**	**0.2790**	<u>0.2006</u>	0.4580	<u>0.7870</u>	<u>0.1924</u>	*Persistence (Baseline)*
	39.2799	0.0418	0.0385	0.0457	0.5000	0.0374	*Random (Baseline)*
	24.9639	0.0694	0.0371	0.5309	0.5059	0.0363	Linear Regression
	24.9639	0.0694	0.0371	0.5310	0.5059	0.0363	Ridge
**Regression**	24.9643	0.0695	0.0370	0.5584	0.5048	0.0362	Lasso
	25.0019	0.0721	0.0374	<u>1.0000</u>	0.5417	0.0407	Decision Tree
	24.8842	0.0831	0.0437	0.8452	0.5704	0.0430	Random Forest
	24.9327	0.0958	0.0525	0.5476	0.6161	0.0501	XGBoost
	24.9586	0.0837	0.0461	0.4488	0.5756	0.0440	MLP
		**0.2790**	<u>0.2006</u>	0.4580	<u>0.7870</u>	<u>0.1924</u>	*Persistence (Baseline)*
		0.0418	0.0385	0.0457	0.5000	0.0374	*Random (Baseline)*
		0.1064	0.0599	0.4783	0.6398	0.0580	Logistic Regression
**Classification**		0.0828	0.0791	0.0869	0.6677	0.0638	Decision Tree
		0.1194	0.0801	0.2342	0.6709	0.0673	Random Forest
		0.1203	0.0811	0.2330	0.6768	0.0671	XGBoost
		0.1088	0.0608	0.5170	0.6550	0.0613	MLP
**Procurement**							
	37.2930	0.0481	<u>0.1974</u>	0.0274	0.5125	0.0275	*Persistence (Baseline)*
	33.5045	0.0182	0.0177	0.0188	0.5000	0.0187	*Random (Baseline)*
	26.0912	0.0414	0.0212	<u>0.9122</u>	0.6487	0.0325	Linear Regression
	26.0912	0.0414	0.0212	0.9120	0.6486	0.0325	Ridge
**Regression**	26.0912	0.0414	0.0212	0.9118	0.6484	0.0325	Lasso
	26.0987	0.0354	0.0182	0.7006	0.4789	0.0181	Decision Tree
	26.0841	0.0459	0.0236	0.8545	0.6333	0.0265	Random Forest
	26.0790	0.0536	0.0279	0.6995	0.6609	0.0314	XGBoost
	**26.0779**	0.0423	0.0218	0.7861	0.6026	0.0285	MLP
		0.0481	<u>0.1974</u>	0.0274	0.5125	0.0275	*Persistence (Baseline)*
		0.0182	0.0177	0.0188	0.5000	0.0187	*Random (Baseline)*
		0.1163	0.0738	0.2732	0.7621	0.0594	Logistic Regression
**Classification**		0.1371	0.1004	0.2161	0.7856	0.0752	Decision Tree
		0.1322	0.0876	0.2694	0.7816	0.0723	Random Forest
		**0.1390**	0.1151	0.1755	<u>0.7878</u>	<u>0.0789</u>	XGBoost
		0.1293	0.0894	0.2340	0.7741	0.0706	MLP

S12.1.8 Baseline Features/Embedding-derived features/Relatedness

Table S25: Comparison of baseline and advanced models for regression and classification tasks across Trade and Procurement domains, for the ablation excluding the economic complexity features except relatedness (i.e. using the baseline, relatedness, and embedding-derived features). Models are ordered from simpler to more complex. Baseline models are marked in italics. The best model for RMSE and F1 is highlighted, and the best for Precision/Recall/ROC-AUC and Average Precision (AP) is underlined. For ROC-AUC and AP the random baselines are the by-construction rates, i.e. 0.5 for ROC-AUC and prevalence for AP.

Domain	Test RMSE	Test F1	Test Precision	Test Recall	Test ROC-AUC	Test AP	Model
**International Trade**							
	**20.7366**	**0.2790**	<u>0.2006</u>	0.4580	<u>0.7870</u>	<u>0.1924</u>	*Persistence (Baseline)*
	39.2799	0.0418	0.0385	0.0457	0.5000	0.0374	*Random (Baseline)*
	24.9641	0.0769	0.0413	0.5585	0.5310	0.0390	Linear Regression
	24.9641	0.0769	0.0413	0.5585	0.5310	0.0390	Ridge
**Regression**	24.9429	0.0777	0.0416	0.5993	0.5392	0.0404	Lasso
	24.0533	0.0810	0.0429	0.7235	0.5653	0.0418	Decision Tree
	22.4477	0.0851	0.0448	0.8507	0.5696	0.0418	Random Forest
	24.2519	0.1031	0.0571	0.5305	0.6418	0.0538	XGBoost
	23.7357	0.0721	0.0374	<u>1.0000</u>	0.5000	0.0374	MLP
		**0.2790**	<u>0.2006</u>	0.4580	<u>0.7870</u>	<u>0.1924</u>	*Persistence (Baseline)*
		0.0418	0.0385	0.0457	0.5000	0.0374	*Random (Baseline)*
		0.1157	0.0698	0.3362	0.6551	0.0629	Logistic Regression
**Classification**		0.0703	0.0917	0.0570	0.6829	0.0686	Decision Tree
		0.1215	0.0823	0.2315	0.6793	0.0689	Random Forest
		0.0932	0.0907	0.0958	0.6853	0.0694	XGBoost
		0.1089	0.0595	0.6354	0.6676	0.0644	MLP
**Procurement**							
	37.2930	0.0481	0.1974	0.0274	0.5125	0.0275	*Persistence (Baseline)*
	33.5045	0.0182	0.0177	0.0188	0.5000	0.0187	*Random (Baseline)*
	25.6217	0.0547	0.0283	0.7842	0.7154	0.0623	Linear Regression
	25.6217	0.0547	0.0283	0.7842	0.7154	0.0623	Ridge
**Regression**	25.6221	0.0550	0.0285	0.7811	0.7151	0.0621	Lasso
	25.3360	0.1003	0.0543	0.6510	0.7927	0.0547	Decision Tree
	26.9867	0.0553	0.0285	<u>0.9113</u>	0.7341	0.0372	Random Forest
	24.6795	0.1220	0.0665	0.7327	0.8439	0.0858	XGBoost
	**24.5863**	0.0957	0.0517	0.6405	0.7774	0.0606	MLP
		0.0481	0.1974	0.0274	0.5125	0.0275	*Persistence (Baseline)*
		0.0182	0.0177	0.0188	0.5000	0.0187	*Random (Baseline)*
		0.2396	0.1739	0.3849	0.8867	0.1551	Logistic Regression
**Classification**		0.2629	0.2155	0.3371	0.9003	0.1883	Decision Tree
		0.2541	0.1903	0.3825	0.8965	0.1737	Random Forest
		**0.2672**	<u>0.2410</u>	0.2998	<u>0.9031</u>	<u>0.1942</u>	XGBoost
		0.2637	0.2033	0.3751	0.8988	0.1857	MLP

S12.1.9 Baseline features/relatedness

Table S26: Comparison of baseline and advanced models for regression and classification tasks across Trade and Procurement domains, for the ablation using only the baseline features and the relatedness feature. Models are ordered from simpler to more complex. Baseline models are marked in italics. The best model for RMSE and F1 is highlighted, and the best for Precision/Recall/ROC-AUC and Average Precision (AP) is underlined. For ROC-AUC and AP the random baselines are the by-construction rates, i.e. 0.5 for ROC-AUC and prevalence for AP.

Domain	Test RMSE	Test F1	Test Precision	Test Recall	Test ROC-AUC	Test AP	Model
**International Trade**							
**Regression**	**20.7366**	**0.2790**	<u>0.2006</u>	0.4580	<u>0.7870</u>	<u>0.1924</u>	*Persistence (Baseline)*
	39.2799	0.0418	0.0385	0.0457	0.5000	0.0374	*Random (Baseline)*
	24.9637	0.0713	0.0382	0.5319	0.5153	0.0372	Linear Regression
	24.9637	0.0713	0.0382	0.5320	0.5153	0.0372	Ridge
	24.9641	0.0710	0.0379	0.5586	0.5127	0.0369	Lasso
	24.9787	0.0825	0.0432	0.8940	0.5260	0.0396	Decision Tree
	24.9039	0.0778	0.0407	<u>0.9034</u>	0.5239	0.0379	Random Forest
	24.9394	0.0995	0.0547	0.5526	0.6330	0.0521	XGBoost
	24.9287	0.0885	0.0475	0.6585	0.5881	0.0452	MLP
**Classification**		**0.2790**	<u>0.2006</u>	0.4580	<u>0.7870</u>	<u>0.1924</u>	*Persistence (Baseline)*
		0.0418	0.0385	0.0457	0.5000	0.0374	*Random (Baseline)*
		0.1163	0.0701	0.3411	0.6550	0.0629	Logistic Regression
		0.0721	0.0942	0.0583	0.6829	0.0688	Decision Tree
		0.1227	0.0827	0.2380	0.6809	0.0696	Random Forest
		0.0960	0.0922	0.1002	0.6862	0.0697	XGBoost
		0.1169	0.0708	0.3353	0.6644	0.0640	MLP
**Procurement**							
**Regression**	37.2930	0.0481	0.1974	0.0274	0.5125	0.0275	*Persistence (Baseline)*
	33.5045	0.0182	0.0177	0.0188	0.5000	0.0187	*Random (Baseline)*
	25.8688	0.0782	0.0409	<u>0.8649</u>	0.8310	0.1149	Linear Regression
	25.8688	0.0782	0.0409	<u>0.8649</u>	0.8310	0.1149	Ridge
	25.8710	0.0824	0.0433	0.8449	0.8266	0.1134	Lasso
	25.8637	0.1052	0.0569	0.7011	0.8089	0.0653	Decision Tree
	**25.4602**	0.0939	0.0497	0.8576	0.8478	0.0908	Random Forest
	25.6477	0.1256	0.0685	0.7556	0.8551	0.0991	XGBoost
	25.8335	0.0884	0.0468	0.7868	0.7979	0.0719	MLP
**Classification**		0.0481	0.1974	0.0274	0.5125	0.0275	*Persistence (Baseline)*
		0.0182	0.0177	0.0188	0.5000	0.0187	*Random (Baseline)*
		0.2334	0.1994	0.2813	0.8882	0.1534	Logistic Regression
		0.2614	0.1942	0.3993	0.8999	0.1845	Decision Tree
		0.2505	0.1786	0.4192	0.8968	0.1749	Random Forest
		**0.2695**	0.2186	0.3515	<u>0.9024</u>	<u>0.1938</u>	XGBoost
		0.2623	<u>0.2273</u>	0.3101	0.8987	0.1882	MLP

S12.2 Ranking

S12.2.1 No EC features

Table S27: Entry-ranking evaluation: Precision@k and Recall@k for regression and classification models across Trade and Procurement domains, for the ablation excluding the economic complexity features (including relatedness) from the full feature set. Global metrics pool all candidates into a single ranking ($k \in \{100, 500, 1000\}$); per-entity metrics are computed within each entity's candidate set and macro-averaged ($k \in \{5, 10, 20, 50\}$). Best precision and recall within each domain × scope × k group (across all models, baselines included) are in bold.

	Global (pooled)			Per-entity (macro-avg)		
Model	k	P@k	R@k	k	P@k	R@k
**Trade**						
Classification						
XGBoost	100	0.0700	0.0002	5	**0.0723**	**0.0123**
	500	0.0700	0.0009	10	**0.0735**	**0.0253**
	1000	0.0800	0.0022	20	**0.0697**	**0.0449**
				50	**0.0645**	**0.0983**
Random Forest	100	0.1400	**0.0004**	5	**0.0723**	0.0119
	500	0.0940	0.0013	10	0.0704	0.0214
	1000	0.1060	0.0029	20	0.0650	0.0383
				50	0.0601	0.0859
MLP	100	0.0900	0.0002	5	0.0477	0.0091
	500	0.0680	0.0009	10	0.0463	0.0163
	1000	0.0690	0.0019	20	0.0446	0.0304
				50	0.0437	0.0683
Decision Tree	100	0.0600	0.0002	5	0.0702	0.0116
	500	0.0560	0.0008	10	0.0686	0.0222
	1000	0.0690	0.0019	20	0.0660	0.0395
				50	0.0606	0.0908
Logistic Regression	100	**0.1500**	**0.0004**	5	0.0441	0.0055
	500	**0.1340**	**0.0018**	10	0.0455	0.0116
	1000	**0.1250**	**0.0034**	20	0.0446	0.0227
				50	0.0465	0.0585
Regression						
XGBoost	100	0.0700	0.0002	5	0.0540	0.0094
	500	0.0260	0.0004	10	0.0528	0.0175
	1000	0.0370	0.0010	20	0.0553	0.0345
				50	0.0553	0.0856
Linear Regression	100	0.0100	0.0000	5	0.0405	0.0058
	500	0.0160	0.0002	10	0.0408	0.0117
	1000	0.0180	0.0005	20	0.0405	0.0233
				50	0.0408	0.0546
Ridge	100	0.0100	0.0000	5	0.0405	0.0058
	500	0.0160	0.0002	10	0.0408	0.0117
	1000	0.0180	0.0005	20	0.0405	0.0233

Table S27 – continued

Model	Global (pooled)			Per-entity (macro-avg)		
	k	P@k	R@k	k	P@k	R@k
				50	0.0408	0.0546
Lasso	100	0.0000	0.0000	5	0.0403	0.0053
	500	0.0340	0.0005	10	0.0374	0.0096
	1000	0.0360	0.0010	20	0.0357	0.0174
				50	0.0366	0.0446
MLP	100	0.0400	0.0001	5	0.0466	0.0060
	500	0.0520	0.0007	10	0.0456	0.0118
	1000	0.0470	0.0013	20	0.0461	0.0246
				50	0.0456	0.0591
Decision Tree	100	0.0100	0.0000	5	0.0399	0.0057
	500	0.0040	0.0001	10	0.0412	0.0118
	1000	0.0030	0.0001	20	0.0415	0.0222
				50	0.0407	0.0515
Random Forest	100	0.0400	0.0001	5	0.0431	0.0060
	500	0.0360	0.0005	10	0.0444	0.0130
	1000	0.0340	0.0009	20	0.0449	0.0255
				50	0.0437	0.0622
**Procurement**						
Classification						
XGBoost	100	**0.2900**	**0.0013**	5	**0.1274**	**0.1330**
	500	0.2700	0.0061	10	**0.1047**	**0.2080**
	1000	**0.2480**	**0.0112**	20	**0.0855**	**0.3272**
				50	**0.0599**	**0.5486**
Random Forest	100	0.2100	0.0009	5	0.1218	0.1301
	500	0.2160	0.0049	10	0.1011	0.2067
	1000	0.2190	0.0099	20	0.0831	0.3184
				50	0.0593	0.5412
MLP	100	0.2300	0.0010	5	0.1118	0.1174
	500	0.2180	0.0049	10	0.0917	0.1843
	1000	0.1840	0.0083	20	0.0763	0.2903
				50	0.0567	0.5157
Decision Tree	100	**0.2900**	**0.0013**	5	0.1169	0.1208
	500	**0.2740**	**0.0062**	10	0.0987	0.1963
	1000	0.2260	0.0102	20	0.0829	0.3163
				50	0.0596	0.5460
Logistic Regression	100	0.0900	0.0004	5	0.0995	0.1085
	500	0.0640	0.0014	10	0.0831	0.1692
	1000	0.0910	0.0041	20	0.0732	0.2774
				50	0.0553	0.4991
Regression						
XGBoost	100	0.0100	0.0000	5	0.0301	0.0275
	500	0.0060	0.0001	10	0.0303	0.0567

Table S27 – continued

	Global (pooled)			Per-entity (macro-avg)		
Model	k	P@k	R@k	k	P@k	R@k
	1000	0.0140	0.0006	20	0.0289	0.1056
				50	0.0269	0.2377
Linear Regression	100	0.0000	0.0000	5	0.0232	0.0189
	500	0.0160	0.0004	10	0.0236	0.0417
	1000	0.0200	0.0009	20	0.0239	0.0853
				50	0.0233	0.2037
Ridge	100	0.0000	0.0000	5	0.0232	0.0189
	500	0.0160	0.0004	10	0.0236	0.0417
	1000	0.0200	0.0009	20	0.0239	0.0853
				50	0.0233	0.2037
Lasso	100	0.0000	0.0000	5	0.0234	0.0189
	500	0.0160	0.0004	10	0.0241	0.0425
	1000	0.0200	0.0009	20	0.0243	0.0867
				50	0.0236	0.2065
MLP	100	0.0100	0.0000	5	0.0234	0.0229
	500	0.0040	0.0001	10	0.0236	0.0451
	1000	0.0120	0.0005	20	0.0225	0.0843
				50	0.0221	0.2035
Decision Tree	100	0.0800	0.0004	5	0.0373	0.0434
	500	0.0200	0.0005	10	0.0292	0.0620
	1000	0.0300	0.0014	20	0.0253	0.0998
				50	0.0223	0.2083
Random Forest	100	0.0000	0.0000	5	0.0174	0.0197
	500	0.0020	0.0000	10	0.0185	0.0392
	1000	0.0020	0.0001	20	0.0196	0.0782
				50	0.0231	0.2149

S12.2.2 No Embedding features

Table S28: Entry-ranking evaluation: Precision@k and Recall@k for regression and classification models across Trade and Procurement domains, for the ablation excluding the embedding-derived features from the full feature set. Global metrics pool all candidates into a single ranking ($k \in \{100, 500, 1000\}$); per-entity metrics are computed within each entity's candidate set and macro-averaged ($k \in \{5, 10, 20, 50\}$). Best precision and recall within each domain × scope × k group (across all models, baselines included) are in bold.

	Global (pooled)			Per-entity (macro-avg)		
Model	k	P@k	R@k	k	P@k	R@k
**Trade**						
Classification						
XGBoost	100	0.2700	0.0007	5	0.1532	0.0268
	500	0.2360	0.0032	10	0.1375	0.0455
	1000	**0.2260**	**0.0061**	20	0.1201	0.0752
				50	0.1015	0.1517
Random Forest	100	0.2600	0.0007	5	0.1565	**0.0284**
	500	0.1920	0.0026	10	0.1386	**0.0490**
	1000	0.1780	0.0048	20	**0.1243**	**0.0788**
				50	**0.1032**	**0.1553**
MLP	100	0.2300	0.0006	5	0.1368	0.0240
	500	0.1720	0.0023	10	0.1243	0.0416
	1000	0.1680	0.0045	20	0.1072	0.0679
				50	0.0925	0.1340
Decision Tree	100	**0.2900**	**0.0008**	5	**0.1578**	0.0266
	500	**0.2580**	**0.0035**	10	**0.1398**	0.0473
	1000	0.2230	0.0060	20	0.1220	0.0780
				50	0.1020	0.1537
Logistic Regression	100	0.1800	0.0005	5	0.1151	0.0225
	500	0.1320	0.0018	10	0.1091	0.0400
	1000	0.1210	0.0033	20	0.0995	0.0678
				50	0.0831	0.1307
Regression						
XGBoost	100	0.0800	0.0002	5	0.0884	0.0135
	500	0.0840	0.0011	10	0.0864	0.0269
	1000	0.0770	0.0021	20	0.0857	0.0522
				50	0.0793	0.1158
Linear Regression	100	0.0500	0.0001	5	0.0254	0.0046
	500	0.0280	0.0004	10	0.0269	0.0085
	1000	0.0290	0.0008	20	0.0302	0.0184
				50	0.0397	0.0616
Ridge	100	0.0500	0.0001	5	0.0254	0.0046
	500	0.0280	0.0004	10	0.0268	0.0084
	1000	0.0290	0.0008	20	0.0301	0.0184
				50	0.0397	0.0615

Table S28 – continued

Model	Global (pooled)			Per-entity (macro-avg)		
	k	P@k	R@k	k	P@k	R@k
Lasso	100	0.0500	0.0001	5	0.0252	0.0045
	500	0.0280	0.0004	10	0.0266	0.0084
	1000	0.0290	0.0008	20	0.0298	0.0184
				50	0.0390	0.0611
MLP	100	0.0500	0.0001	5	0.0718	0.0103
	500	0.0780	0.0011	10	0.0765	0.0214
	1000	0.0790	0.0021	20	0.0758	0.0428
				50	0.0699	0.0984
Decision Tree	100	0.0000	0.0000	5	0.0605	0.0084
	500	0.0420	0.0006	10	0.0596	0.0167
	1000	0.0500	0.0014	20	0.0611	0.0345
				50	0.0638	0.0883
Random Forest	100	0.0600	0.0002	5	0.0702	0.0095
	500	0.0580	0.0008	10	0.0682	0.0190
	1000	0.0600	0.0016	20	0.0666	0.0373
				50	0.0670	0.0919
**Procurement**						
Classification						
XGBoost	100	0.5200	0.0023	5	**0.2741**	**0.3058**
	500	**0.5400**	**0.0122**	10	**0.2147**	**0.4494**
	1000	**0.5130**	**0.0231**	20	**0.1549**	**0.6139**
				50	**0.0886**	**0.8224**
Random Forest	100	0.5100	0.0023	5	0.2663	0.3012
	500	0.4440	0.0100	10	0.2067	0.4392
	1000	0.4270	0.0193	20	0.1514	0.6046
				50	0.0877	0.8157
MLP	100	0.5400	0.0024	5	0.2693	0.3007
	500	0.5140	0.0116	10	0.2125	0.4435
	1000	0.5060	0.0228	20	0.1546	0.6127
				50	0.0883	0.8211
Decision Tree	100	**0.6000**	**0.0027**	5	0.2641	0.2961
	500	0.5300	0.0120	10	0.2089	0.4405
	1000	0.4950	0.0223	20	0.1526	0.6063
				50	0.0878	0.8186
Logistic Regression	100	0.0100	0.0000	5	0.2437	0.2801
	500	0.1760	0.0040	10	0.1958	0.4170
	1000	0.2460	0.0111	20	0.1474	0.5898
				50	0.0872	0.8147
Regression						
XGBoost	100	0.2400	0.0011	5	0.1850	0.2149
	500	0.2360	0.0053	10	0.1613	0.3496
	1000	0.2220	0.0100	20	0.1292	0.5223

Table S28 – continued

Model	Global (pooled)			Per-entity (macro-avg)		
	k	P@k	R@k	k	P@k	R@k
				50	0.0826	0.7798
Linear Regression	100	0.0200	0.0001	5	0.1756	0.2013
	500	0.1340	0.0030	10	0.1470	0.3083
	1000	0.1730	0.0078	20	0.1137	0.4506
				50	0.0716	0.6554
Ridge	100	0.0200	0.0001	5	0.1756	0.2013
	500	0.1340	0.0030	10	0.1470	0.3083
	1000	0.1730	0.0078	20	0.1137	0.4506
				50	0.0716	0.6554
Lasso	100	0.0200	0.0001	5	0.1735	0.1978
	500	0.1340	0.0030	10	0.1451	0.3039
	1000	0.1740	0.0078	20	0.1120	0.4415
				50	0.0708	0.6465
MLP	100	0.1400	0.0006	5	0.1434	0.1595
	500	0.1400	0.0032	10	0.1317	0.2757
	1000	0.1360	0.0061	20	0.1106	0.4375
				50	0.0734	0.6792
Decision Tree	100	0.0100	0.0000	5	0.1010	0.1222
	500	0.0540	0.0012	10	0.0946	0.2113
	1000	0.1010	0.0046	20	0.0834	0.3397
				50	0.0645	0.6097
Random Forest	100	0.1700	0.0008	5	0.1484	0.1759
	500	0.1540	0.0035	10	0.1341	0.2914
	1000	0.1690	0.0076	20	0.1117	0.4566
				50	0.0771	0.7325

S12.2.3 No Relatedness

Table S29: Entry-ranking evaluation: Precision@k and Recall@k for regression and classification models across Trade and Procurement domains, for the ablation excluding the relatedness feature from the full feature set. Global metrics pool all candidates into a single ranking ($k \in \{100, 500, 1000\}$); per-entity metrics are computed within each entity's candidate set and macro-averaged ($k \in \{5, 10, 20, 50\}$). Best precision and recall within each domain × scope × k group (across all models, baselines included) are in bold.

	Global (pooled)			Per-entity (macro-avg)		
Model	k	P@k	R@k	k	P@k	R@k
**Trade**						
Classification						
XGBoost	100	**0.3400**	**0.0009**	5	**0.1571**	**0.0278**
	500	**0.2440**	**0.0033**	10	**0.1392**	0.0455
	1000	0.2120	0.0057	20	**0.1215**	0.0767
				50	0.1016	**0.1547**
Random Forest	100	0.3200	**0.0009**	5	0.1500	0.0266
	500	0.2020	0.0027	10	0.1370	**0.0470**
	1000	0.1780	0.0048	20	0.1211	**0.0790**
				50	0.1016	0.1518
MLP	100	0.1900	0.0005	5	0.1441	0.0265
	500	0.1420	0.0019	10	0.1326	0.0455
	1000	0.1370	0.0037	20	0.1154	0.0734
				50	0.0958	0.1398
Decision Tree	100	0.3000	0.0008	5	0.1557	0.0270
	500	**0.2440**	**0.0033**	10	0.1358	0.0455
	1000	**0.2230**	**0.0060**	20	0.1193	0.0761
				50	**0.1017**	0.1525
Logistic Regression	100	0.1400	0.0004	5	0.1275	0.0239
	500	0.1200	0.0016	10	0.1121	0.0394
	1000	0.1270	0.0034	20	0.0997	0.0671
				50	0.0795	0.1227
Regression						
XGBoost	100	0.0200	0.0001	5	0.0735	0.0123
	500	0.0520	0.0007	10	0.0756	0.0251
	1000	0.0590	0.0016	20	0.0754	0.0488
				50	0.0755	0.1137
Linear Regression	100	0.0000	0.0000	5	0.0380	0.0057
	500	0.0160	0.0002	10	0.0415	0.0132
	1000	0.0220	0.0006	20	0.0422	0.0266
				50	0.0430	0.0617
Ridge	100	0.0000	0.0000	5	0.0380	0.0057
	500	0.0160	0.0002	10	0.0415	0.0132
	1000	0.0220	0.0006	20	0.0422	0.0266
				50	0.0430	0.0617

Table S29 – continued

Model	Global (pooled)			Per-entity (macro-avg)		
	k	P@k	R@k	k	P@k	R@k
Lasso	100	0.0000	0.0000	5	0.0384	0.0059
	500	0.0140	0.0002	10	0.0411	0.0125
	1000	0.0220	0.0006	20	0.0419	0.0261
				50	0.0432	0.0622
MLP	100	0.0300	0.0001	5	0.0626	0.0097
	500	0.0480	0.0006	10	0.0597	0.0177
	1000	0.0570	0.0015	20	0.0613	0.0363
				50	0.0612	0.0903
Decision Tree	100	0.0500	0.0001	5	0.0525	0.0072
	500	0.0500	0.0007	10	0.0513	0.0140
	1000	0.0480	0.0013	20	0.0527	0.0307
				50	0.0557	0.0779
Random Forest	100	0.0200	0.0001	5	0.0557	0.0074
	500	0.0460	0.0006	10	0.0608	0.0177
	1000	0.0470	0.0013	20	0.0619	0.0345
				50	0.0664	0.0924
**Procurement**						
Classification						
XGBoost	100	**0.3600**	**0.0016**	5	**0.1468**	**0.1601**
	500	**0.2960**	**0.0067**	10	**0.1163**	**0.2374**
	1000	**0.2730**	**0.0123**	20	**0.0909**	**0.3529**
				50	**0.0625**	**0.5783**
Random Forest	100	0.2900	0.0013	5	0.1377	0.1479
	500	0.2540	0.0057	10	0.1109	0.2257
	1000	0.2510	0.0113	20	0.0862	0.3323
				50	0.0607	0.5588
MLP	100	0.2500	0.0011	5	0.1145	0.1229
	500	0.1940	0.0044	10	0.0981	0.1984
	1000	0.1620	0.0073	20	0.0806	0.3068
				50	0.0593	0.5385
Decision Tree	100	0.3200	0.0014	5	0.1416	0.1550
	500	0.2880	0.0065	10	0.1130	0.2338
	1000	0.2560	0.0115	20	0.0874	0.3395
				50	0.0612	0.5670
Logistic Regression	100	0.1700	0.0008	5	0.1202	0.1341
	500	0.1800	0.0041	10	0.0925	0.1963
	1000	0.1880	0.0085	20	0.0760	0.2960
				50	0.0562	0.5129
Regression						
XGBoost	100	0.0000	0.0000	5	0.0314	0.0298
	500	0.0120	0.0003	10	0.0319	0.0603
	1000	0.0160	0.0007	20	0.0304	0.1129

Table S29 – continued

Model	Global (pooled)			Per-entity (macro-avg)		
	k	P@k	R@k	k	P@k	R@k
				50	0.0280	0.2486
Linear Regression	100	0.0100	0.0000	5	0.0235	0.0199
	500	0.0180	0.0004	10	0.0245	0.0448
	1000	0.0190	0.0009	20	0.0246	0.0881
				50	0.0241	0.2155
Ridge	100	0.0100	0.0000	5	0.0235	0.0199
	500	0.0180	0.0004	10	0.0245	0.0448
	1000	0.0190	0.0009	20	0.0246	0.0881
				50	0.0241	0.2155
Lasso	100	0.0100	0.0000	5	0.0235	0.0199
	500	0.0180	0.0004	10	0.0246	0.0448
	1000	0.0200	0.0009	20	0.0246	0.0885
				50	0.0241	0.2162
MLP	100	0.0000	0.0000	5	0.0276	0.0295
	500	0.0000	0.0000	10	0.0321	0.0681
	1000	0.0030	0.0001	20	0.0331	0.1355
				50	0.0315	0.2997
Decision Tree	100	0.0100	0.0000	5	0.0171	0.0193
	500	0.0060	0.0001	10	0.0191	0.0419
	1000	0.0060	0.0003	20	0.0227	0.0954
				50	0.0257	0.2503
Random Forest	100	0.0000	0.0000	5	0.0136	0.0125
	500	0.0000	0.0000	10	0.0150	0.0290
	1000	0.0040	0.0002	20	0.0163	0.0618
				50	0.0201	0.1882

S12.2.4 EC-only

Table S30: Entry-ranking evaluation: Precision@k and Recall@k for regression and classification models across Trade and Procurement domains, for the ablation using only the economic complexity features (including relatedness). Global metrics pool all candidates into a single ranking ($k \in \{100, 500, 1000\}$); per-entity metrics are computed within each entity's candidate set and macro-averaged ($k \in \{5, 10, 20, 50\}$). Best precision and recall within each domain × scope × k group (across all models, baselines included) are in bold.

	Global (pooled)			Per-entity (macro-avg)		
Model	k	P@k	R@k	k	P@k	R@k
**Trade**						
Classification						
XGBoost	100	**0.3500**	**0.0009**	5	**0.1582**	0.0251
	500	0.2220	0.0030	10	0.1370	0.0443
	1000	0.2210	0.0060	20	**0.1197**	0.0737
				50	**0.0983**	**0.1419**
Random Forest	100	0.2000	0.0005	5	0.1534	**0.0274**
	500	0.2020	0.0027	10	**0.1399**	**0.0473**
	1000	0.1870	0.0051	20	0.1188	**0.0742**
				50	0.0969	0.1388
MLP	100	0.3200	**0.0009**	5	0.1330	0.0207
	500	0.2220	0.0030	10	0.1201	0.0350
	1000	0.2080	0.0056	20	0.1061	0.0619
				50	0.0939	0.1287
Decision Tree	100	0.3100	0.0008	5	0.1517	0.0266
	500	**0.2320**	**0.0031**	10	0.1356	0.0440
	1000	**0.2310**	**0.0063**	20	0.1186	0.0711
				50	0.0963	0.1386
Logistic Regression	100	0.1200	0.0003	5	0.1218	0.0228
	500	0.1340	0.0018	10	0.1121	0.0398
	1000	0.1110	0.0030	20	0.0991	0.0664
				50	0.0790	0.1204
Regression						
XGBoost	100	0.0500	0.0001	5	0.0905	0.0135
	500	0.0720	0.0010	10	0.0884	0.0250
	1000	0.0920	0.0025	20	0.0834	0.0464
				50	0.0783	0.1050
Linear Regression	100	0.0300	0.0001	5	0.0315	0.0046
	500	0.0480	0.0006	10	0.0354	0.0125
	1000	0.0370	0.0010	20	0.0359	0.0251
				50	0.0405	0.0632
Ridge	100	0.0300	0.0001	5	0.0315	0.0046
	500	0.0480	0.0006	10	0.0354	0.0125
	1000	0.0360	0.0010	20	0.0359	0.0251
				50	0.0405	0.0632

Table S30 – continued

Model	Global (pooled)			Per-entity (macro-avg)		
	k	P@k	R@k	k	P@k	R@k
Lasso	100	0.0300	0.0001	5	0.0315	0.0046
	500	0.0480	0.0006	10	0.0353	0.0127
	1000	0.0370	0.0010	20	0.0361	0.0252
				50	0.0405	0.0630
MLP	100	0.1300	0.0004	5	0.0826	0.0114
	500	0.1180	0.0016	10	0.0817	0.0222
	1000	0.0920	0.0025	20	0.0784	0.0455
				50	0.0736	0.1020
Decision Tree	100	0.0700	0.0002	5	0.0641	0.0089
	500	0.0620	0.0008	10	0.0608	0.0173
	1000	0.0510	0.0014	20	0.0614	0.0332
				50	0.0607	0.0807
Random Forest	100	0.0400	0.0001	5	0.0739	0.0097
	500	0.0620	0.0008	10	0.0731	0.0203
	1000	0.0580	0.0016	20	0.0691	0.0366
				50	0.0667	0.0903
**Procurement**						
Classification						
XGBoost	100	0.4600	0.0021	5	0.2485	0.2810
	500	0.4780	0.0108	10	0.1971	0.4172
	1000	0.4550	0.0205	20	**0.1485**	**0.5948**
				50	**0.0871**	**0.8118**
Random Forest	100	0.4800	0.0022	5	0.2486	0.2824
	500	0.4720	0.0106	10	0.1945	0.4114
	1000	0.4860	0.0219	20	0.1476	0.5921
				50	0.0869	0.8109
MLP	100	0.4600	0.0021	5	**0.2530**	0.2863
	500	**0.5260**	**0.0119**	10	**0.2002**	**0.4230**
	1000	**0.4980**	**0.0225**	20	0.1481	0.5942
				50	0.0867	0.8103
Decision Tree	100	**0.5000**	**0.0023**	5	0.2516	**0.2866**
	500	0.5220	0.0118	10	0.1974	0.4167
	1000	0.4860	0.0219	20	0.1480	0.5933
				50	0.0869	0.8112
Logistic Regression	100	0.0100	0.0000	5	0.2161	0.2455
	500	0.1500	0.0034	10	0.1779	0.3814
	1000	0.2180	0.0098	20	0.1372	0.5505
				50	0.0842	0.7855
Regression						
XGBoost	100	0.1300	0.0006	5	0.1417	0.1636
	500	0.1580	0.0036	10	0.1359	0.2940
	1000	0.1280	0.0058	20	0.1149	0.4702

Table S30 – continued

Model	Global (pooled)			Per-entity (macro-avg)		
	k	P@k	R@k	k	P@k	R@k
				50	0.0775	0.7395
Linear Regression	100	0.0300	0.0001	5	0.1693	0.1935
	500	0.1360	0.0031	10	0.1418	0.2955
	1000	0.1820	0.0082	20	0.1099	0.4300
				50	0.0696	0.6329
Ridge	100	0.0300	0.0001	5	0.1693	0.1935
	500	0.1360	0.0031	10	0.1418	0.2955
	1000	0.1820	0.0082	20	0.1099	0.4300
				50	0.0696	0.6329
Lasso	100	0.0300	0.0001	5	0.1693	0.1935
	500	0.1360	0.0031	10	0.1418	0.2955
	1000	0.1820	0.0082	20	0.1099	0.4300
				50	0.0696	0.6329
MLP	100	0.1400	0.0006	5	0.1320	0.1517
	500	0.1380	0.0031	10	0.1282	0.2777
	1000	0.1310	0.0059	20	0.1071	0.4386
				50	0.0710	0.6821
Decision Tree	100	0.1100	0.0005	5	0.1067	0.1245
	500	0.1020	0.0023	10	0.1090	0.2434
	1000	0.0950	0.0043	20	0.0974	0.4049
				50	0.0697	0.6725
Random Forest	100	0.1100	0.0005	5	0.1186	0.1357
	500	0.1540	0.0035	10	0.1191	0.2634
	1000	0.1340	0.0060	20	0.1039	0.4318
				50	0.0730	0.7007

S12.2.5 Embedding-derived features only

Table S31: Entry-ranking evaluation: Precision@k and Recall@k for regression and classification models across Trade and Procurement domains, for the ablation using only the embedding-derived features. Global metrics pool all candidates into a single ranking ($k \in \{100, 500, 1000\}$); per-entity metrics are computed within each entity's candidate set and macro-averaged ($k \in \{5, 10, 20, 50\}$). Best precision and recall within each domain × scope × k group (across all models, baselines included) are in bold.

	Global (pooled)			Per-entity (macro-avg)		
Model	k	P@k	R@k	k	P@k	R@k
**Trade**						
Classification						
XGBoost	100	0.0800	0.0002	5	0.0496	0.0062
	500	0.1080	0.0015	10	0.0478	0.0121
	1000	**0.1060**	**0.0029**	20	**0.0506**	**0.0266**
				50	**0.0483**	0.0606
Random Forest	100	0.0800	0.0002	5	**0.0504**	**0.0067**
	500	0.1100	0.0015	10	**0.0492**	**0.0130**
	1000	0.1020	0.0028	20	0.0488	0.0256
				50	0.0480	**0.0620**
MLP	100	0.0900	0.0002	5	0.0420	0.0057
	500	0.0860	0.0012	10	0.0434	0.0115
	1000	0.0860	0.0023	20	0.0415	0.0226
				50	0.0417	0.0541
Decision Tree	100	0.1300	**0.0004**	5	0.0498	0.0061
	500	0.1020	0.0014	10	0.0464	0.0120
	1000	0.0990	0.0027	20	0.0456	0.0232
				50	0.0431	0.0548
Logistic Regression	100	**0.1600**	**0.0004**	5	0.0477	0.0057
	500	**0.1160**	**0.0016**	10	0.0441	0.0114
	1000	**0.1060**	**0.0029**	20	0.0426	0.0216
				50	0.0432	0.0550
Regression						
XGBoost	100	0.0400	0.0001	5	0.0450	0.0054
	500	0.0340	0.0005	10	0.0449	0.0110
	1000	0.0320	0.0009	20	0.0438	0.0220
				50	0.0425	0.0529
Linear Regression	100	0.0000	0.0000	5	0.0426	**0.0067**
	500	0.0140	0.0002	10	0.0428	0.0121
	1000	0.0140	0.0004	20	0.0430	0.0227
				50	0.0414	0.0531
Ridge	100	0.0000	0.0000	5	0.0426	**0.0067**
	500	0.0140	0.0002	10	0.0428	0.0121
	1000	0.0140	0.0004	20	0.0431	0.0227
				50	0.0414	0.0531

Table S31 – continued

	Global (pooled)			Per-entity (macro-avg)		
Model	k	P@k	R@k	k	P@k	R@k
Lasso	100	0.0000	0.0000	5	0.0403	0.0053
	500	0.0340	0.0005	10	0.0374	0.0096
	1000	0.0360	0.0010	20	0.0357	0.0174
				50	0.0366	0.0446
MLP	100	0.0500	0.0001	5	0.0282	0.0037
	500	0.0540	0.0007	10	0.0306	0.0088
	1000	0.0450	0.0012	20	0.0294	0.0160
				50	0.0296	0.0383
Decision Tree	100	0.0200	0.0001	5	0.0382	0.0052
	500	0.0220	0.0003	10	0.0372	0.0096
	1000	0.0400	0.0011	20	0.0367	0.0180
				50	0.0372	0.0462
Random Forest	100	0.0300	0.0001	5	0.0454	0.0058
	500	0.0300	0.0004	10	0.0411	0.0100
	1000	0.0300	0.0008	20	0.0407	0.0209
				50	0.0404	0.0515
**Procurement**						
Classification						
XGBoost	100	0.2100	0.0009	5	**0.0789**	**0.0791**
	500	**0.1840**	**0.0041**	10	0.0669	0.1300
	1000	**0.1470**	**0.0066**	20	0.0551	0.2079
				50	0.0455	0.4213
Random Forest	100	0.0200	0.0001	5	0.0682	0.0648
	500	0.0500	0.0011	10	0.0632	0.1222
	1000	0.0530	0.0024	20	0.0548	0.2074
				50	0.0448	0.4138
MLP	100	0.0200	0.0001	5	0.0726	0.0702
	500	0.0280	0.0006	10	0.0641	0.1213
	1000	0.0260	0.0012	20	0.0572	0.2119
				50	**0.0482**	**0.4439**
Decision Tree	100	0.2100	0.0009	5	0.0754	0.0759
	500	0.1180	0.0027	10	0.0661	0.1314
	1000	0.1060	0.0048	20	0.0548	0.2078
				50	0.0457	0.4211
Logistic Regression	100	**0.3200**	**0.0014**	5	0.0787	0.0764
	500	0.1720	0.0039	10	**0.0685**	**0.1316**
	1000	0.1240	0.0056	20	**0.0577**	**0.2160**
				50	0.0459	0.4217
Regression						
XGBoost	100	0.0100	0.0000	5	0.0328	0.0317
	500	0.0160	0.0004	10	0.0328	0.0619
	1000	0.0160	0.0007	20	0.0309	0.1143

Table S31 – continued

Model	Global (pooled)			Per-entity (macro-avg)		
	k	P@k	R@k	k	P@k	R@k
				50	0.0269	0.2387
Linear Regression	100	0.0000	0.0000	5	0.0239	0.0197
	500	0.0200	0.0005	10	0.0251	0.0446
	1000	0.0230	0.0010	20	0.0260	0.0931
				50	0.0249	0.2176
Ridge	100	0.0000	0.0000	5	0.0239	0.0197
	500	0.0200	0.0005	10	0.0251	0.0446
	1000	0.0230	0.0010	20	0.0260	0.0931
				50	0.0249	0.2176
Lasso	100	0.0000	0.0000	5	0.0240	0.0198
	500	0.0200	0.0005	10	0.0254	0.0449
	1000	0.0230	0.0010	20	0.0261	0.0934
				50	0.0251	0.2196
MLP	100	0.0000	0.0000	5	0.0270	0.0248
	500	0.0180	0.0004	10	0.0266	0.0468
	1000	0.0170	0.0008	20	0.0240	0.0833
				50	0.0211	0.1808
Decision Tree	100	0.0200	0.0001	5	0.0280	0.0293
	500	0.0400	0.0009	10	0.0261	0.0518
	1000	0.0400	0.0018	20	0.0260	0.0998
				50	0.0247	0.2251
Random Forest	100	0.0000	0.0000	5	0.0184	0.0170
	500	0.0100	0.0002	10	0.0185	0.0332
	1000	0.0120	0.0005	20	0.0208	0.0761
				50	0.0224	0.1971

S12.2.6 Relatedness-only

Table S32: Entry-ranking evaluation: Precision@k and Recall@k for regression and classification models across Trade and Procurement domains, for the ablation using only the relatedness feature. Global metrics pool all candidates into a single ranking ($k \in \{100, 500, 1000\}$); per-entity metrics are computed within each entity's candidate set and macro-averaged ($k \in \{5, 10, 20, 50\}$). Best precision and recall within each domain × scope × k group (across all models, baselines included) are in bold.

	Global (pooled)			Per-entity (macro-avg)		
Model	k	P@k	R@k	k	P@k	R@k
**Trade**						
Classification						
XGBoost	100	0.0400	0.0001	5	0.0420	0.0057
	500	0.1080	**0.0015**	10	0.0396	0.0101
	1000	0.0960	0.0026	20	0.0409	0.0204
				50	0.0412	0.0522
Random Forest	100	0.0900	0.0002	5	0.0576	0.0087
	500	0.0800	0.0011	10	0.0505	0.0143
	1000	0.0860	0.0023	20	0.0478	0.0251
				50	0.0474	0.0600
MLP	100	0.0300	0.0001	5	0.0662	0.0110
	500	0.0420	0.0006	10	0.0618	0.0197
	1000	0.0520	0.0014	20	0.0580	0.0343
				50	0.0553	0.0770
Decision Tree	100	**0.1500**	**0.0004**	5	0.0458	0.0063
	500	**0.1120**	**0.0015**	10	0.0433	0.0113
	1000	**0.1060**	**0.0029**	20	0.0429	0.0224
				50	0.0426	0.0529
Logistic Regression	100	0.0800	0.0002	5	**0.0683**	**0.0113**
	500	0.0620	0.0008	10	**0.0632**	**0.0200**
	1000	0.0680	0.0018	20	0.0594	0.0349
				50	**0.0565**	**0.0784**
Regression						
XGBoost	100	0.0100	0.0000	5	0.0473	0.0063
	500	0.0120	0.0002	10	0.0441	0.0112
	1000	0.0180	0.0005	20	0.0416	0.0210
				50	0.0428	0.0552
Linear Regression	100	0.0800	0.0002	5	0.0681	0.0112
	500	0.0600	0.0008	10	0.0630	0.0199
	1000	0.0730	0.0020	20	**0.0595**	**0.0350**
				50	**0.0565**	**0.0784**
Ridge	100	0.0800	0.0002	5	0.0681	**0.0113**
	500	0.0600	0.0008	10	**0.0632**	**0.0200**
	1000	0.0730	0.0020	20	**0.0595**	0.0349
				50	**0.0565**	**0.0784**

Table S32 – continued

Model	Global (pooled)			Per-entity (macro-avg)		
	k	P@k	R@k	k	P@k	R@k
Lasso	100	0.0800	0.0002	5	0.0676	0.0112
	500	0.0600	0.0008	10	**0.0632**	**0.0200**
	1000	0.0530	0.0014	20	**0.0595**	0.0349
				50	**0.0565**	**0.0784**
MLP	100	0.0200	0.0001	5	0.0498	0.0091
	500	0.0100	0.0001	10	0.0481	0.0164
	1000	0.0140	0.0004	20	0.0451	0.0285
				50	0.0436	0.0645
Decision Tree	100	0.0100	0.0000	5	0.0408	0.0055
	500	0.0120	0.0002	10	0.0375	0.0097
	1000	0.0100	0.0003	20	0.0358	0.0178
				50	0.0366	0.0449
Random Forest	100	0.0400	0.0001	5	0.0401	0.0051
	500	0.0360	0.0005	10	0.0418	0.0102
	1000	0.0400	0.0011	20	0.0423	0.0215
				50	0.0428	0.0524
**Procurement**						
Classification						
XGBoost	100	0.2400	**0.0011**	5	0.2090	**0.2405**
	500	**0.2800**	**0.0063**	10	**0.1729**	**0.3658**
	1000	**0.2820**	**0.0127**	20	0.1339	**0.5413**
				50	0.0845	0.7926
Random Forest	100	0.1600	0.0007	5	0.1912	0.2272
	500	0.1900	0.0043	10	0.1632	0.3551
	1000	0.1900	0.0086	20	0.1301	0.5323
				50	0.0839	0.7880
MLP	100	0.0100	0.0000	5	0.2082	0.2399
	500	0.1520	0.0034	10	0.1724	0.3655
	1000	0.2080	0.0094	20	**0.1341**	0.5406
				50	**0.0846**	**0.7928**
Decision Tree	100	0.1400	0.0006	5	0.1863	0.2228
	500	0.1880	0.0042	10	0.1600	0.3512
	1000	0.1790	0.0081	20	0.1287	0.5303
				50	0.0835	0.7878
Logistic Regression	100	0.0100	0.0000	5	0.2082	0.2399
	500	0.1520	0.0034	10	0.1724	0.3655
	1000	0.2080	0.0094	20	**0.1341**	0.5406
				50	**0.0846**	**0.7928**
Regression						
XGBoost	100	**0.2500**	**0.0011**	5	**0.2094**	0.2392
	500	0.2640	0.0060	10	0.1721	0.3653
	1000	0.2410	0.0109	20	0.1330	0.5362

Table S32 – continued

Model	Global (pooled)			Per-entity (macro-avg)		
	k	P@*k*	R@*k*	*k*	P@*k*	R@*k*
				50	0.0841	0.7859
Linear Regression	100	0.0100	0.0000	5	0.2082	0.2399
	500	0.1520	0.0034	10	0.1724	0.3655
	1000	0.2080	0.0094	20	**0.1341**	0.5406
				50	**0.0846**	**0.7928**
Ridge	100	0.0100	0.0000	5	0.2082	0.2399
	500	0.1520	0.0034	10	0.1724	0.3655
	1000	0.2080	0.0094	20	**0.1341**	0.5406
				50	**0.0846**	**0.7928**
Lasso	100	0.0100	0.0000	5	0.2082	0.2399
	500	0.1520	0.0034	10	0.1724	0.3655
	1000	0.2080	0.0094	20	**0.1341**	0.5406
				50	**0.0846**	**0.7928**
MLP	100	0.0100	0.0000	5	0.2082	0.2399
	500	0.1520	0.0034	10	0.1724	0.3655
	1000	0.2080	0.0094	20	**0.1341**	0.5406
				50	**0.0846**	**0.7928**
Decision Tree	100	0.1500	0.0007	5	0.1913	0.2191
	500	0.1900	0.0043	10	0.1571	0.3346
	1000	0.2450	0.0111	20	0.1230	0.4935
				50	0.0778	0.7197
Random Forest	100	0.0500	0.0002	5	0.1808	0.2167
	500	0.1140	0.0026	10	0.1592	0.3446
	1000	0.1560	0.0070	20	0.1283	0.5213
				50	0.0831	0.7818

S12.2.7 Baseline Features

Table S33: Entry-ranking evaluation: Precision@k and Recall@k for regression and classification models across Trade and Procurement domains, for the ablation using only the baseline features, i.e. excluding both the economic complexity and the embedding-derived features. Global metrics pool all candidates into a single ranking ($k \in \{100, 500, 1000\}$); per-entity metrics are computed within each entity's candidate set and macro-averaged ($k \in \{5, 10, 20, 50\}$). Best precision and recall within each domain × scope × k group (across all models, baselines included) are in bold.

	Global (pooled)			Per-entity (macro-avg)		
Model	k	P@k	R@k	k	P@k	R@k
**Trade**						
Classification						
XGBoost	100	0.0400	0.0001	5	**0.0779**	**0.0141**
	500	0.0680	0.0009	10	**0.0784**	**0.0255**
	1000	0.0810	0.0022	20	**0.0700**	**0.0452**
				50	**0.0657**	**0.0996**
Random Forest	100	**0.1500**	**0.0004**	5	0.0716	0.0115
	500	**0.1300**	**0.0018**	10	0.0721	0.0215
	1000	**0.1180**	**0.0032**	20	0.0666	0.0398
				50	0.0633	0.0928
MLP	100	0.0500	0.0001	5	0.0599	0.0109
	500	0.0420	0.0006	10	0.0612	0.0228
	1000	0.0580	0.0016	20	0.0586	0.0406
				50	0.0528	0.0845
Decision Tree	100	0.1000	0.0003	5	0.0737	0.0121
	500	0.0720	0.0010	10	0.0731	0.0233
	1000	0.0630	0.0017	20	0.0684	0.0424
				50	0.0615	0.0918
Logistic Regression	100	0.1300	**0.0004**	5	0.0391	0.0041
	500	0.1080	0.0015	10	0.0395	0.0082
	1000	0.1040	0.0028	20	0.0435	0.0187
				50	0.0427	0.0466
Regression						
XGBoost	100	0.0400	0.0001	5	0.0582	0.0103
	500	0.0580	0.0008	10	0.0545	0.0201
	1000	0.0510	0.0014	20	0.0544	0.0369
				50	0.0536	0.0844
Linear Regression	100	0.0500	0.0001	5	0.0261	0.0040
	500	0.0240	0.0003	10	0.0262	0.0075
	1000	0.0260	0.0007	20	0.0291	0.0164
				50	0.0297	0.0443
Ridge	100	0.0500	0.0001	5	0.0261	0.0040
	500	0.0240	0.0003	10	0.0262	0.0075
	1000	0.0260	0.0007	20	0.0291	0.0164
				50	0.0296	0.0442

Table S33 – continued

Model	Global (pooled)			Per-entity (macro-avg)		
	k	P@k	R@k	k	P@k	R@k
Lasso	100	0.0400	0.0001	5	0.0261	0.0040
	500	0.0240	0.0003	10	0.0262	0.0075
	1000	0.0260	0.0007	20	0.0289	0.0163
				50	0.0289	0.0416
MLP	100	0.0300	0.0001	5	0.0527	0.0090
	500	0.0320	0.0004	10	0.0524	0.0174
	1000	0.0490	0.0013	20	0.0506	0.0337
				50	0.0489	0.0768
Decision Tree	100	0.0200	0.0001	5	0.0303	0.0044
	500	0.0200	0.0003	10	0.0325	0.0085
	1000	0.0180	0.0005	20	0.0349	0.0175
				50	0.0357	0.0441
Random Forest	100	0.0300	0.0001	5	0.0481	0.0078
	500	0.0320	0.0004	10	0.0497	0.0157
	1000	0.0280	0.0008	20	0.0503	0.0310
				50	0.0486	0.0736
**Procurement**						
Classification						
XGBoost	100	**0.3600**	**0.0016**	5	**0.1248**	**0.1332**
	500	**0.2780**	**0.0063**	10	**0.1018**	0.2075
	1000	**0.2570**	**0.0116**	20	**0.0843**	**0.3233**
				50	**0.0592**	**0.5480**
Random Forest	100	0.2700	0.0012	5	0.1174	0.1266
	500	0.1920	0.0043	10	0.1016	**0.2077**
	1000	0.1940	0.0088	20	0.0816	0.3125
				50	0.0580	0.5318
MLP	100	0.2400	0.0011	5	0.1142	0.1304
	500	0.2060	0.0046	10	0.0886	0.1885
	1000	0.1960	0.0088	20	0.0759	0.2962
				50	0.0561	0.5184
Decision Tree	100	0.3300	0.0015	5	0.1186	0.1216
	500	0.2620	0.0059	10	0.0994	0.1978
	1000	0.2430	0.0110	20	0.0821	0.3168
				50	0.0586	0.5391
Logistic Regression	100	0.0700	0.0003	5	0.1020	0.1129
	500	0.1220	0.0028	10	0.0825	0.1684
	1000	0.1270	0.0057	20	0.0716	0.2686
				50	0.0544	0.4937
Regression						
XGBoost	100	0.0100	0.0000	5	0.0484	0.0578
	500	0.0460	0.0010	10	0.0423	0.0906
	1000	0.0540	0.0024	20	0.0394	0.1600

Table S33 – continued

Model	Global (pooled)			Per-entity (macro-avg)		
	k	P@k	R@k	k	P@k	R@k
				50	0.0341	0.3216
Linear Regression	100	0.0300	0.0001	5	0.0685	0.0889
	500	0.0360	0.0008	10	0.0627	0.1446
	1000	0.0430	0.0019	20	0.0559	0.2382
				50	0.0433	0.4230
Ridge	100	0.0300	0.0001	5	0.0685	0.0889
	500	0.0360	0.0008	10	0.0627	0.1446
	1000	0.0430	0.0019	20	0.0559	0.2382
				50	0.0433	0.4230
Lasso	100	0.0300	0.0001	5	0.0689	0.0895
	500	0.0360	0.0008	10	0.0622	0.1435
	1000	0.0430	0.0019	20	0.0559	0.2382
				50	0.0433	0.4230
MLP	100	0.0100	0.0000	5	0.0492	0.0536
	500	0.0700	0.0016	10	0.0440	0.0893
	1000	0.0810	0.0037	20	0.0376	0.1443
				50	0.0306	0.2813
Decision Tree	100	0.0100	0.0000	5	0.0217	0.0246
	500	0.0480	0.0011	10	0.0204	0.0423
	1000	0.0520	0.0023	20	0.0209	0.0805
				50	0.0197	0.1821
Random Forest	100	0.0100	0.0000	5	0.0331	0.0388
	500	0.0120	0.0003	10	0.0320	0.0682
	1000	0.0080	0.0004	20	0.0307	0.1214
				50	0.0305	0.2812

S12.2.8 Baseline Features/Embedding-derived features/Relatedness

Table S34: Entry-ranking evaluation: Precision@k and Recall@k for regression and classification models across Trade and Procurement domains, for the ablation excluding the economic complexity features except relatedness (i.e. using the baseline, relatedness, and embedding-derived features). Global metrics pool all candidates into a single ranking ($k \in \{100, 500, 1000\}$); per-entity metrics are computed within each entity's candidate set and macro-averaged ($k \in \{5, 10, 20, 50\}$). Best precision and recall within each domain × scope × k group (across all models, baselines included) are in bold.

	Global (pooled)			Per-entity (macro-avg)		
Model	k	P@k	R@k	k	P@k	R@k
**Trade**						
Classification						
XGBoost	100	0.1100	0.0003	5	**0.0786**	**0.0133**
	500	0.1180	0.0016	10	**0.0755**	**0.0255**
	1000	0.1080	0.0029	20	**0.0704**	**0.0458**
				50	**0.0653**	**0.1003**
Random Forest	100	**0.1500**	**0.0004**	5	0.0718	0.0108
	500	**0.1220**	**0.0017**	10	0.0682	0.0214
	1000	**0.1130**	**0.0031**	20	0.0654	0.0401
				50	0.0612	0.0877
MLP	100	0.0700	0.0002	5	0.0628	0.0124
	500	0.0900	0.0012	10	0.0613	0.0219
	1000	0.0870	0.0024	20	0.0599	0.0410
				50	0.0545	0.0840
Decision Tree	100	0.0900	0.0002	5	0.0716	0.0113
	500	0.1160	0.0016	10	0.0684	0.0224
	1000	0.1050	0.0028	20	0.0648	0.0416
				50	0.0615	0.0899
Logistic Regression	100	0.0600	0.0002	5	0.0559	0.0069
	500	0.0580	0.0008	10	0.0533	0.0136
	1000	0.0710	0.0019	20	0.0507	0.0254
				50	0.0475	0.0601
Regression						
XGBoost	100	0.0300	0.0001	5	0.0532	0.0090
	500	0.0300	0.0004	10	0.0536	0.0196
	1000	0.0360	0.0010	20	0.0532	0.0355
				50	0.0526	0.0827
Linear Regression	100	0.0100	0.0000	5	0.0410	0.0059
	500	0.0160	0.0002	10	0.0401	0.0115
	1000	0.0180	0.0005	20	0.0400	0.0230
				50	0.0404	0.0542
Ridge	100	0.0100	0.0000	5	0.0410	0.0059
	500	0.0160	0.0002	10	0.0401	0.0115
	1000	0.0180	0.0005	20	0.0400	0.0230

Table S34 – continued

Model	Global (pooled)			Per-entity (macro-avg)		
	k	P@k	R@k	k	P@k	R@k
				50	0.0405	0.0542
Lasso	100	0.0000	0.0000	5	0.0458	0.0063
	500	0.0120	0.0002	10	0.0426	0.0129
	1000	0.0190	0.0005	20	0.0426	0.0240
				50	0.0416	0.0547
MLP	100	0.0100	0.0000	5	0.0401	0.0053
	500	0.0340	0.0005	10	0.0375	0.0096
	1000	0.0330	0.0009	20	0.0357	0.0175
				50	0.0366	0.0446
Decision Tree	100	0.0100	0.0000	5	0.0389	0.0052
	500	0.0380	0.0005	10	0.0366	0.0099
	1000	0.0460	0.0012	20	0.0390	0.0203
				50	0.0386	0.0483
Random Forest	100	0.0300	0.0001	5	0.0450	0.0061
	500	0.0320	0.0004	10	0.0422	0.0112
	1000	0.0350	0.0009	20	0.0415	0.0226
				50	0.0421	0.0557
**Procurement**						
Classification						
XGBoost	100	0.5500	0.0025	5	**0.2714**	0.3051
	500	**0.5420**	**0.0122**	10	**0.2124**	0.4453
	1000	**0.5010**	**0.0226**	20	**0.1540**	**0.6093**
				50	**0.0883**	**0.8207**
Random Forest	100	0.5100	0.0023	5	0.2558	0.2930
	500	0.4400	0.0099	10	0.2027	0.4350
	1000	0.4190	0.0189	20	0.1486	0.5961
				50	0.0871	0.8128
MLP	100	0.5400	0.0024	5	0.2699	**0.3052**
	500	0.4640	0.0105	10	0.2099	**0.4457**
	1000	0.4390	0.0198	20	0.1526	0.6075
				50	0.0876	0.8144
Decision Tree	100	**0.6300**	**0.0028**	5	0.2555	0.2905
	500	0.5120	0.0115	10	0.2028	0.4304
	1000	0.4730	0.0213	20	0.1500	0.5973
				50	0.0878	0.8180
Logistic Regression	100	0.0100	0.0000	5	0.2492	0.2873
	500	0.2060	0.0046	10	0.2003	0.4253
	1000	0.2580	0.0116	20	0.1484	0.5927
				50	0.0865	0.8033
Regression						
XGBoost	100	0.1300	0.0006	5	0.1383	0.1715
	500	0.1620	0.0037	10	0.1212	0.2744

Table S34 – continued

Model	Global (pooled)			Per-entity (macro-avg)		
	k	P@k	R@k	k	P@k	R@k
	1000	0.1360	0.0061	20	0.1032	0.4285
				50	0.0743	0.7093
Linear Regression	100	0.0400	0.0002	5	0.0961	0.1052
	500	0.1400	0.0032	10	0.0862	0.1745
	1000	0.1570	0.0071	20	0.0718	0.2702
				50	0.0518	0.4605
Ridge	100	0.0400	0.0002	5	0.0961	0.1052
	500	0.1400	0.0032	10	0.0862	0.1745
	1000	0.1570	0.0071	20	0.0718	0.2702
				50	0.0518	0.4605
Lasso	100	0.0400	0.0002	5	0.0955	0.1045
	500	0.1400	0.0032	10	0.0860	0.1740
	1000	0.1570	0.0071	20	0.0718	0.2704
				50	0.0518	0.4604
MLP	100	0.0700	0.0003	5	0.1083	0.1193
	500	0.0600	0.0014	10	0.1004	0.2103
	1000	0.0760	0.0034	20	0.0882	0.3450
				50	0.0647	0.5905
Decision Tree	100	0.0100	0.0000	5	0.0722	0.0836
	500	0.0180	0.0004	10	0.0740	0.1615
	1000	0.0440	0.0020	20	0.0679	0.2835
				50	0.0580	0.5656
Random Forest	100	0.0000	0.0000	5	0.0483	0.0587
	500	0.0160	0.0004	10	0.0500	0.1138
	1000	0.0230	0.0010	20	0.0493	0.2089
				50	0.0463	0.4472

S12.2.9 Baseline features/relatedness

Table S35: Entry-ranking evaluation: Precision@k and Recall@k for regression and classification models across Trade and Procurement domains, for the ablation using only the baseline features and the relatedness feature. Global metrics pool all candidates into a single ranking ($k \in \{100, 500, 1000\}$); per-entity metrics are computed within each entity's candidate set and macro-averaged ($k \in \{5, 10, 20, 50\}$). Best precision and recall within each domain × scope × k group (across all models, baselines included) are in bold.

	Global (pooled)			Per-entity (macro-avg)		
Model	k	P@k	R@k	k	P@k	R@k
**Trade**						
Classification						
XGBoost	100	0.1000	0.0003	5	0.0784	**0.0131**
	500	0.1000	0.0014	10	0.0752	**0.0243**
	1000	0.1040	0.0028	20	0.0695	**0.0449**
				50	**0.0660**	**0.1016**
Random Forest	100	**0.1300**	**0.0004**	5	0.0796	0.0122
	500	0.1320	0.0018	10	0.0762	0.0225
	1000	**0.1200**	**0.0032**	20	0.0721	0.0442
				50	0.0652	0.0939
MLP	100	0.1200	0.0003	5	0.0712	0.0115
	500	0.1000	0.0014	10	0.0701	0.0228
	1000	0.0980	0.0027	20	0.0635	0.0380
				50	0.0567	0.0808
Decision Tree	100	**0.1300**	**0.0004**	5	0.0754	0.0125
	500	**0.1440**	**0.0019**	10	0.0706	0.0231
	1000	**0.1200**	**0.0032**	20	0.0666	0.0422
				50	0.0636	0.0919
Logistic Regression	100	0.0800	0.0002	5	**0.0876**	0.0122
	500	0.0560	0.0008	10	**0.0806**	0.0215
	1000	0.0720	0.0019	20	**0.0737**	0.0390
				50	0.0625	0.0813
Regression						
XGBoost	100	0.0500	0.0001	5	0.0546	0.0101
	500	0.0480	0.0006	10	0.0545	0.0196
	1000	0.0570	0.0015	20	0.0557	0.0388
				50	0.0542	0.0858
Linear Regression	100	0.0500	0.0001	5	0.0261	0.0040
	500	0.0240	0.0003	10	0.0262	0.0075
	1000	0.0260	0.0007	20	0.0291	0.0164
				50	0.0298	0.0444
Ridge	100	0.0500	0.0001	5	0.0261	0.0040
	500	0.0240	0.0003	10	0.0262	0.0075
	1000	0.0260	0.0007	20	0.0291	0.0164
				50	0.0298	0.0445

Table S35 – continued

Model	Global (pooled)			Per-entity (macro-avg)		
	k	P@k	R@k	k	P@k	R@k
Lasso	100	0.0500	0.0001	5	0.0261	0.0040
	500	0.0240	0.0003	10	0.0262	0.0075
	1000	0.0260	0.0007	20	0.0290	0.0163
				50	0.0288	0.0415
MLP	100	0.0200	0.0001	5	0.0462	0.0072
	500	0.0280	0.0004	10	0.0491	0.0161
	1000	0.0370	0.0010	20	0.0514	0.0336
				50	0.0497	0.0755
Decision Tree	100	0.0400	0.0001	5	0.0368	0.0048
	500	0.0220	0.0003	10	0.0352	0.0090
	1000	0.0170	0.0005	20	0.0344	0.0169
				50	0.0362	0.0441
Random Forest	100	0.0200	0.0001	5	0.0435	0.0068
	500	0.0220	0.0003	10	0.0449	0.0135
	1000	0.0270	0.0007	20	0.0439	0.0265
				50	0.0451	0.0675
**Procurement**						
Classification						
XGBoost	100	0.5900	0.0027	5	**0.2709**	**0.3049**
	500	**0.5540**	**0.0125**	10	**0.2112**	**0.4440**
	1000	**0.5030**	**0.0227**	20	**0.1538**	**0.6094**
				50	**0.0883**	**0.8211**
Random Forest	100	0.5600	0.0025	5	0.2616	0.2950
	500	0.4740	0.0107	10	0.2057	0.4383
	1000	0.4380	0.0198	20	0.1502	0.6007
				50	0.0875	0.8168
MLP	100	**0.6100**	**0.0028**	5	0.2702	0.3029
	500	0.5160	0.0116	10	0.2104	0.4411
	1000	0.4910	0.0221	20	0.1527	0.6048
				50	0.0876	0.8148
Decision Tree	100	0.5400	0.0024	5	0.2570	0.2916
	500	0.5060	0.0114	10	0.2041	0.4327
	1000	0.4690	0.0212	20	0.1501	0.5971
				50	0.0879	0.8187
Logistic Regression	100	0.0100	0.0000	5	0.2460	0.2838
	500	0.2080	0.0047	10	0.1991	0.4236
	1000	0.2590	0.0117	20	0.1493	0.5968
				50	0.0878	0.8183
Regression						
XGBoost	100	0.3000	0.0014	5	0.1499	0.1851
	500	0.2380	0.0054	10	0.1362	0.3034
	1000	0.2090	0.0094	20	0.1146	0.4720

Table S35 – continued

Model	Global (pooled)			Per-entity (macro-avg)		
	k	P@k	R@k	k	P@k	R@k
				50	0.0787	0.7431
Linear Regression	100	0.0100	0.0000	5	0.1801	0.2063
	500	0.1380	0.0031	10	0.1508	0.3199
	1000	0.1750	0.0079	20	0.1167	0.4650
				50	0.0754	0.6963
Ridge	100	0.0100	0.0000	5	0.1801	0.2063
	500	0.1380	0.0031	10	0.1508	0.3199
	1000	0.1750	0.0079	20	0.1167	0.4650
				50	0.0754	0.6963
Lasso	100	0.0100	0.0000	5	0.1775	0.2038
	500	0.1380	0.0031	10	0.1485	0.3139
	1000	0.1800	0.0081	20	0.1148	0.4550
				50	0.0742	0.6846
MLP	100	0.1900	0.0009	5	0.1303	0.1474
	500	0.1660	0.0037	10	0.1178	0.2486
	1000	0.1300	0.0059	20	0.0994	0.3919
				50	0.0703	0.6517
Decision Tree	100	0.0300	0.0001	5	0.0908	0.1093
	500	0.0760	0.0017	10	0.0875	0.1905
	1000	0.0620	0.0028	20	0.0805	0.3235
				50	0.0624	0.5870
Random Forest	100	0.1600	0.0007	5	0.1408	0.1684
	500	0.1640	0.0037	10	0.1275	0.2763
	1000	0.1690	0.0076	20	0.1078	0.4429
				50	0.0771	0.7287

S12.3 Ranking Ablation Summary

Table S36: Entry-ranking performance by feature configuration, reporting global (pooled) Precision@1000 and Recall@1000 and per-entity (macro-averaged) Precision and Recall at $k \in \{10, 50\}$. The model family is **XGBoost**. Two ablation families are shown per task. *Baseline-augmented*: the reference set (baseline features only, i.e. country- and firm-specific features) augmented with one feature set each. *Baseline* is the unaugmented baseline and *Full* is the complete feature set from Table S17. The EC feature set also includes relatedness and as such, the + EC row also includes the relatedness feature. *Standalone*: a single feature group in isolation, with the baseline features excluded. Bold marks the best configuration per column within each ablation case and task block. The acronym *EDF* denotes the embedding-derived feature set.

	Global (pooled)		Per-entity (macro-avg)			
	P@1000	R@1000	P@10	P@50	R@10	R@50
**Trade — Regression**						
Baseline-augmented						
Baseline	0.0510	0.0014	0.0545	0.0536	0.0201	0.0844
+ EC	**0.0770**	**0.0021**	**0.0864**	0.0793	0.0269	0.1158
+ EDF	0.0370	0.0010	0.0528	0.0553	0.0175	0.0856
+ Relatedness	0.0570	0.0015	0.0545	0.0542	0.0196	0.0858
Full	0.0680	0.0018	0.0860	**0.0803**	**0.0276**	**0.1194**
Standalone						
EC only (incl. relatedness)	**0.0920**	**0.0025**	**0.0884**	**0.0783**	**0.0250**	**0.1050**
Relatedness only	0.0180	0.0005	0.0441	0.0428	0.0112	0.0552
EDF only	0.0320	0.0009	0.0449	0.0425	0.0110	0.0529
**Trade — Classification**						
Baseline-augmented						
Baseline	0.0810	0.0022	0.0784	0.0657	0.0255	0.0996
+ EC	0.2260	0.0061	0.1375	0.1015	0.0455	0.1517
+ EDF	0.0800	0.0022	0.0735	0.0645	0.0253	0.0983
+ Relatedness	0.1040	0.0028	0.0752	0.0660	0.0243	0.1016
Full	**0.2690**	**0.0073**	**0.1474**	**0.1074**	**0.0490**	**0.1601**
Standalone						
EC only (incl. relatedness)	**0.2210**	**0.0060**	**0.1370**	**0.0983**	**0.0443**	**0.1419**
Relatedness only	0.0960	0.0026	0.0396	0.0412	0.0101	0.0522
EDF only	0.1060	0.0029	0.0478	0.0483	0.0121	0.0606
**Procurement — Regression**						
Baseline-augmented						
Baseline	0.0540	0.0024	0.0423	0.0341	0.0906	0.3216
+ EC	**0.2220**	**0.0100**	**0.1613**	**0.0826**	**0.3496**	**0.7798**
+ EDF	0.0140	0.0006	0.0303	0.0269	0.0567	0.2377
+ Relatedness	0.2090	0.0094	0.1362	0.0787	0.3034	0.7431
Full	0.1330	0.0060	0.1223	0.0755	0.2748	0.7194
Standalone						
EC only (incl. relatedness)	0.1280	0.0058	0.1359	0.0775	0.2940	0.7395
Relatedness only	**0.2410**	**0.0109**	**0.1721**	**0.0841**	**0.3653**	**0.7859**

Table S36 – continued

	Global (pooled)		Per-entity (macro-avg)			
	P@1000	R@1000	P@10	P@50	R@10	R@50
EDF only	0.0160	0.0007	0.0328	0.0269	0.0619	0.2387
**Procurement — Classification**						
Baseline-augmented						
Baseline	0.2570	0.0116	0.1018	0.0592	0.2075	0.5480
+ EC	0.5130	0.0231	**0.2147**	**0.0886**	**0.4494**	**0.8224**
+ EDF	0.2480	0.0112	0.1047	0.0599	0.2080	0.5486
+ Relatedness	0.5030	0.0227	0.2112	0.0883	0.4440	0.8211
Full	**0.5290**	**0.0239**	0.2132	0.0885	0.4468	0.8220
Standalone						
EC only (incl. relatedness)	**0.4550**	**0.0205**	**0.1971**	**0.0871**	**0.4172**	**0.8118**
Relatedness only	0.2820	0.0127	0.1729	0.0845	0.3658	0.7926
EDF only	0.1470	0.0066	0.0669	0.0455	0.1300	0.4213

References

[1] Ricardo J. G. B. Campello et al. "Density-based clustering". In: *WIREs Data Mining and Knowledge Discovery* 10.2 (Oct. 2019). ISSN: 1942-4795. DOI: 10.1002/widm.1343.

[2] Tara Chari and Lior Pachter. "The specious art of single-cell genomics". In: *PLOS Computational Biology* 19.8 (Aug. 2023). Ed. by Jason A. Papin, e1011288. ISSN: 1553-7358. DOI: 10.1371/journal.pcbi.1011288.

[3] César A. Hidalgo et al. "The Product Space Conditions the Development of Nations". In: *Science* 317.5837 (July 2007), pp. 482–487. ISSN: 1095-9203. DOI: 10.1126/science.1144581.

[4] John R. Hurley and Raymond B. Cattell. "The procrustes program: Producing direct rotation to test a hypothesized factor structure". In: *Behavioral Science* 7.2 (Jan. 1962), pp. 258–262. ISSN: 1099-1743. DOI: 10.1002/bs.3830070216.

[5] Andrey Kutuzov et al. "Diachronic word embeddings and semantic shifts: a survey". In: *Proceedings of the 27th International Conference on Computational Linguistics*. Ed. by Emily M. Bender, Leon Derczynski, and Pierre Isabelle. Santa Fe, New Mexico, USA: Association for Computational Linguistics, Aug. 2018, pp. 1384–1397. URL: https://aclanthology.org/C18-1117/.

[6] Donald B. Rubin. *Multiple Imputation for Nonresponse in Surveys*. Wiley, June 1987. ISBN: 9780470316696. DOI: 10.1002/9780470316696.

[7] Uriel Singer, Ido Guy, and Kira Radinsky. "Node Embedding over Temporal Graphs". In: *Proceedings of the Twenty-Eighth International Joint Conference on Artificial Intelligence*. IJCAI-2019. International Joint Conferences on Artificial Intelligence Organization, Aug. 2019, pp. 4605–4612. DOI: 10.24963/ijcai.2019/640.
